# Dynamic Electrophoretic Assembly Coupled With Water‐Splitting‐Induced pH Control Enables Precision Ion Sieving Membranes

**DOI:** 10.1002/adma.74934

**Published:** 2026-09-09

**Authors:** Mohammad Hossein Jandaghian, Petric Marc Ruya, Ipek Ozyurt, Ali Atyabi, Samuel Eyley, Wim Thielemans, Alexander Volodin, Riccardo Alessandri, Xing Yang

**Affiliations:** ^1^ Process Engineering for Sustainable Systems (ProcESS) Division, Department of Chemical Engineering KU Leuven Heverlee Belgium; ^2^ Sustainable Materials Lab, Department of Chemical Engineering KU Leuven Kortrijk Belgium; ^3^ Quantum Solid‐State Physics Division, Department of Physics and Astronomy KU Leuven Heverlee Belgium; ^4^ Soft Matter, Rheology and Technology (SMaRT) Division, Department of Chemical Engineering KU Leuven Heverlee Belgium

**Keywords:** covalent organic frameworks, monovalent cation separation, thin‐film composite membranes

## Abstract

Discriminating among monovalent ions of similar charge and size, such as Li^+^, Na^+^, and K^+^, remains one of the most persistent challenges in separation science. Here we present a green, external‐acid‐free route to fabricate defect‐free covalent organic framework (COF)‐based thin‐film composite (TFC) membranes for high precision monovalent‐ion sieving. The method relies on a dynamic electric‐field‐assisted strategy in which an alternating electric field couples electrophoretic monomer assembly at the surface of an ion‐exchange membrane (IEM) substrate with spatiotemporally resolved delivery of hydronium ions generated in situ via water splitting. This localized in situ‐generated acid pulse initiates interfacial condensation into primary COF nuclei, which subsequently undergo continuous growth and fusion into a uniform ultrathin film through a self‐healing mechanism. The proposed membrane formation strategy effectively suppresses non‐selective transport pathways and enables rapid formation of highly selective ion‐transport channels, resulting in a membrane with near‐complete fractionation of Li^+^ from other monovalent ions (e.g., K^+^ and Na^+^), while preserving fast ion permeation that exceeds that of previously reported membranes. The governing ion separation mechanism is elucidated as hydration‐shell restructuring under electrostatic confinement. The work establishes a scalable and energy‐efficient platform for precision ion separation and opens new opportunities for membrane‐based resource recovery and molecular purification.

## Graphical abstract

A green, external‐catalyst‐free route grows defect‐free covalent organic framework membranes directly on ion‐exchange supports using an alternating electric field and in situ‐generated acid. Through a self‐regulating, self‐healing mechanism, the membranes achieve near‐complete, ultra‐fast separation of monovalent ions such as Li^+^, Na^+^, and K^+^, offering a new paradigm for precision ion separation.

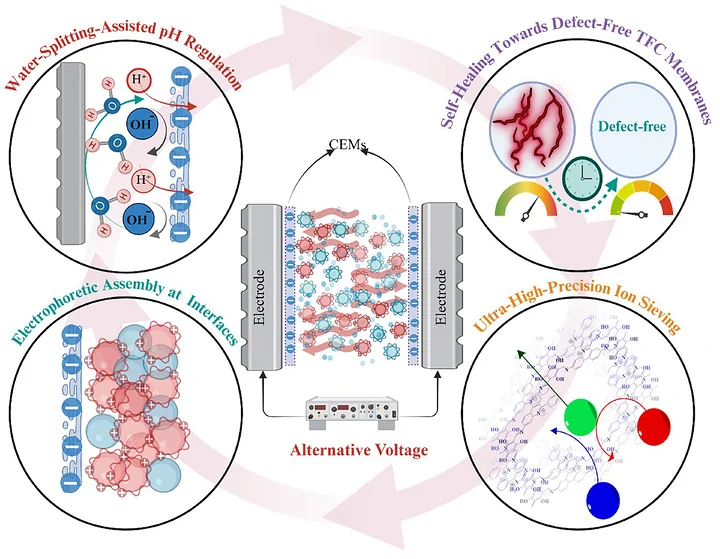

## 1 Introduction

Effective monovalent‐monovalent cation separation has remained one of the most unresolved challenges in separation science and engineering, a defining bottleneck for next‐generation ion‐selective membranes [1, 2, 3, 4]. These ions share identical charge and differ only subtly in size, by a few angstroms, rendering separation based solely on charge or size exclusion intrinsically difficult [5]. The challenge is further exacerbated in realistic feed streams; such as salt lakes, seawater, and battery‐recycling effluents; where target ions are typically present at trace levels [4, 6]. Covalent organic frameworks (COFs) offer a solution through their extended, covalently cross‐linked polymer networks, which provide long‐range continuity and through‐plane connectivity defined by chemical bonds rather than physical stacking or particle packing [4, 7]. Although COF‐based membranes have consequently demonstrated outstanding performance in monovalent‐divalent separations, there are only limited reports claiming monovalent‐monovalent selectivity, and mostly by introducing ion‐recognition functionalities into well‐defined COF channels (typically 1–5 nm) [4, 8, 9]. This persistent ceiling reveals that, for monovalent ions, intrinsic channel selectivity alone is insufficient; suppression of defects and preservation of structural homogeneity at the molecular scale are decisive.

The structural limitations are closely coupled with membrane fabrication methods [10, 11]. For example, the rapid formation of an initial COF layer in conventional interfacial polymerization (IP), which relies heavily on toxic organic solvents, suppresses further monomer transport across immiscible phases, leading to under‐linked networks, incomplete film growth, and mechanically fragile membranes [12]. Such defects are particularly detrimental for monovalent‐monovalent separations, where even a small fraction of non‐selective pathways can undermine selectivity [13].

Recently, electric‐field‐assisted fabrication has emerged as a promising strategy to overcome the diffusion‐limited growth of COF films by accelerating reaction kinetics and redistributing charged species [14, 15, 16]. However, current approaches; including capacitive assembly at interfaces [15], pure electrophoretic deposition [16], and electrochemical interfacial polymerization [14]; still suffer from important limitations. For example, in pure electrophoretic deposition, pre‐synthesized COF nanosheets are physically deposited onto a support under an applied electric field [16]. In such systems, the electric field mainly acts as a driving force for the electrophoretic migration of already‐formed particles dispersed in solution, while no interfacial condensation reaction occurs during the deposition process itself. As a result, the strategy relies solely on the physical accumulation of charged nanosheets on the substrate surface, which does not address the formation of interparticle ion‐transport shortcuts. In contrast, electrochemical interfacial polymerization methods involve an electrochemical polymerization process in which charged monomers; either intrinsically charged species (e.g., ethidium bromide [17]) or temporarily charged through externally added organic acids (e.g., para‐diaminobenzene [14]); are driven by the electric field to accumulate on the surface of an electroconductive substrate. In these systems, electrons directly participate in the reaction environment, making conductive supports essential for electron transfer, neutralization of charged intermediates, and sustaining polymerization [14, 17].

As a matter of fact, capacitive assembly at interfaces typically produces brittle free‐standing films, whereas electrochemical growth at electrode/support interfaces requires electroconductive supports [14, 15, 16, 17, 18]. Moreover, both previously reported methods are dependent on external catalysts (e.g., organic acids). Collectively, these factors severely restrict film homogeneity, fabrication scalability, and applicability across different COF chemistries.

To address the above‐mentioned limitations, here we introduce a new paradigm for fabricating COF‐based thin‐film composite (TFC) membranes under a dynamic electric field, without the need for external catalysts or conductive substrates. This approach features a unique combined mechanism: (i) a locally enriched micrometer‐thick layer of COF‐forming monomers at the ion‐exchange substrate surface, and (ii) a transient and spatiotemporally directed delivery of in situ‐generated hydronium ions induced by water splitting to facilitate localized formation of COF primary particles. The mechanism decouples COF growth from static interfaces, enabling controlled nucleation and continuous film formation through a self‐healing mechanism that suppresses non‐selective transport pathways and promotes ultra‐fast single‐ion selectivity.

## 2 Results and Discussion

### 2.1 Electrophoretic Assembly at Interface

A custom‐built membrane fabrication setup (Figure 1a,b) was employed in this work, and the underlying mechanism of COF‐based TFC membrane formation on the commercial cation‐exchange membrane (CEM) surfaces within this configuration is schematically depicted in Figure 1c. Upon applying an electric potential between the electrodes, charged monomeric and oligomeric species migrate toward oppositely charged poles under an electric field; a process analogous to electrophoresis. The migration of these monomers leads to their accumulation near the membrane surface. The CEMs are dense, non‐porous ion‐exchange membranes whose free‐volume elements, on the order of a few angstroms [19], are far smaller than the migrating condensation products (the DFT‐calculated dimer and trimer measure approximately 0.7 and 1.2 nm end‐to‐end, respectively), so that the oligomers cannot traverse the support and instead concentrate at the interface; the sulfonated species are additionally subject to Donnan exclusion by the negatively charged sulfonate matrix. This confinement also prevents their direct electrochemical oxidation or reduction at the electrode surfaces, yielding a deposition mechanism fundamentally different from existing electrochemical IP methods [14, 20].

**FIGURE 1:**
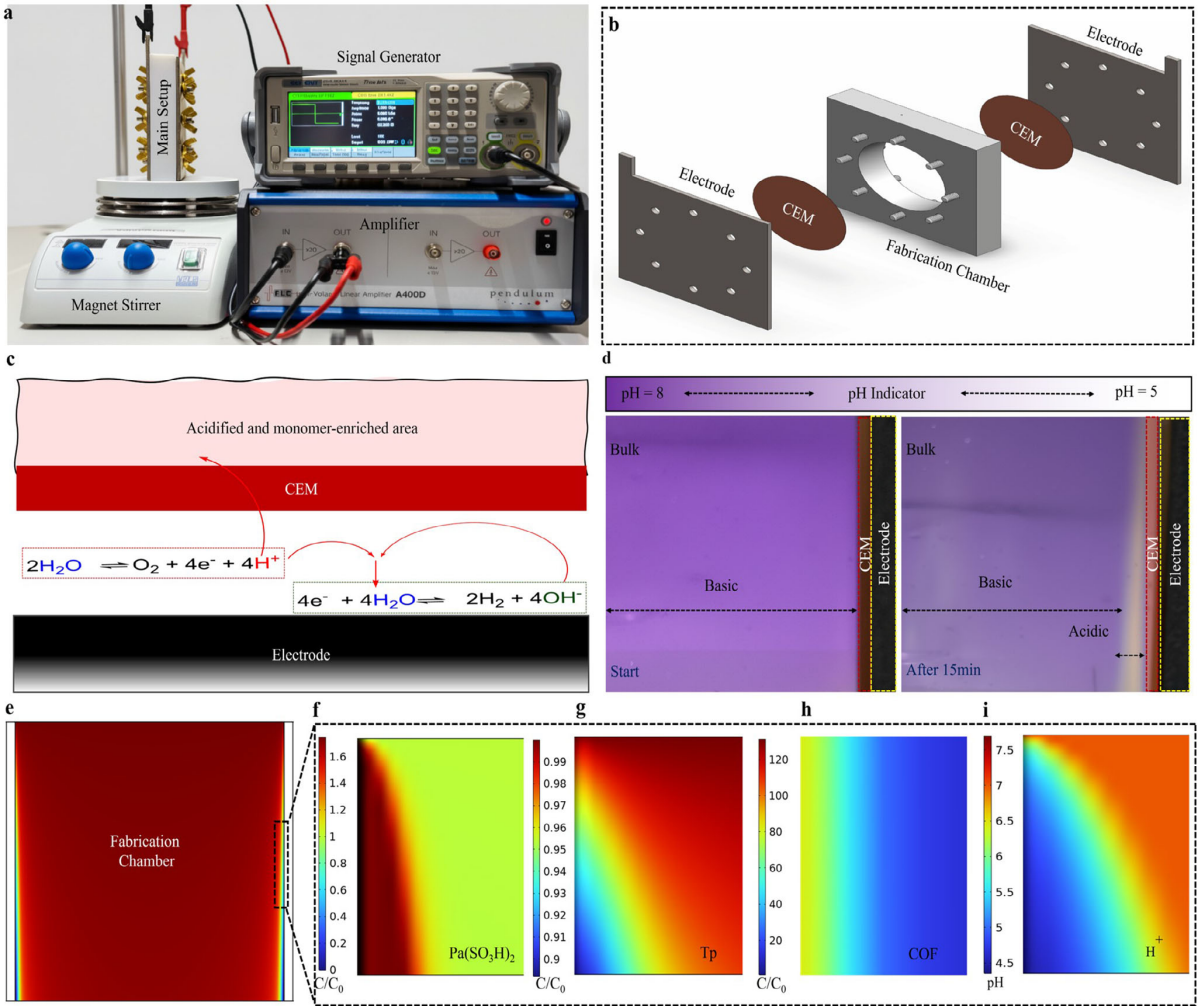
Dynamic electrophoretic assembly and pH control. (a) Photograph of the custom‐built apparatus used for fabricating COF‐coated TFC membranes. (b) Exploded schematic of the laboratory‐designed fabrication chamber highlighting its main components. (c) Schematic illustration of the proposed mechanism of COF‐coated TFC membrane formation; water splitting at the electrode interfaces generates hydronium ions that migrate through the CEM support. (d) Photographs showing pH changes near the CEM surfaces resulting from hydronium ion migration (Figure S1). (e–i) Computational fluid dynamics (CFD) simulation results showing (e) the simulated fabrication chamber and spatial distributions of (f) the charged monomer Pa(SO_3_H)_2_ (2,5‐diaminobenzene‐1,4‐disulfonic acid), (g) the uncharged monomer Tp (2,4,6‐trihydroxybenzene‐1,3,5‐tricarbaldehyde), (h) the COF product, and (i) pH near the CEM surfaces.

This electrophoretic accumulation results in the formation of monomer‐enriched zones adjacent to the CEM interfaces, where the local monomer (e.g., Pa) concentration exceeds that of the bulk solution (Figure 1f). Concurrently, as the IP reaction continues, a directional diffusive flux of the uncharged monomer (e.g., Tp) occurs from the bulk toward the reaction zone to sustain polymerization (Figure 1g). The combined effects of electrophoretic monomer migration and diffusion‐driven replenishment lead to a progressive increase in the interfacial concentration of primary COF nuclei (Figure 1h), which subsequently coalesce and adhere to the CEM surface, forming a uniform COF‐based TFC membrane.

### 2.2 Water‐Splitting‐Assisted pH Regulation at Interfaces

The polycondensation reaction between amine and aldehyde monomers to form COFs typically proceeds slowly and often requires prolonged reaction times (72–120 h), elevated temperatures (100°C–120°C), or the use of acidic or basic catalysts to enhance the rate of imine bond formation [17]. Results from DFT calculations (Figure 2c; Figures S9 and SS10) reveal that the transition states associated with C─N bond formation and dehydration of the carbinolamine intermediate can be stabilized in the presence of H^+^ by approximately 6.6 and 34 kcal mol^−1^, respectively. However, the addition of external acids or bases; or increasing the reaction temperature; tends to promote homogeneous nucleation of COF particles in the bulk solution [21, 22]. This premature particle formation significantly reduces their electrophoretic mobility toward the membrane surface and may even lead to particle sedimentation within the fabrication chamber. Moreover, uncontrolled bulk nucleation increases the likelihood of non‐uniform deposition and coarse particle aggregation on the CEM surfaces, which can detrimentally affect the ion‐selective transport performance of the final membrane (Figure S12).

**FIGURE 2:**
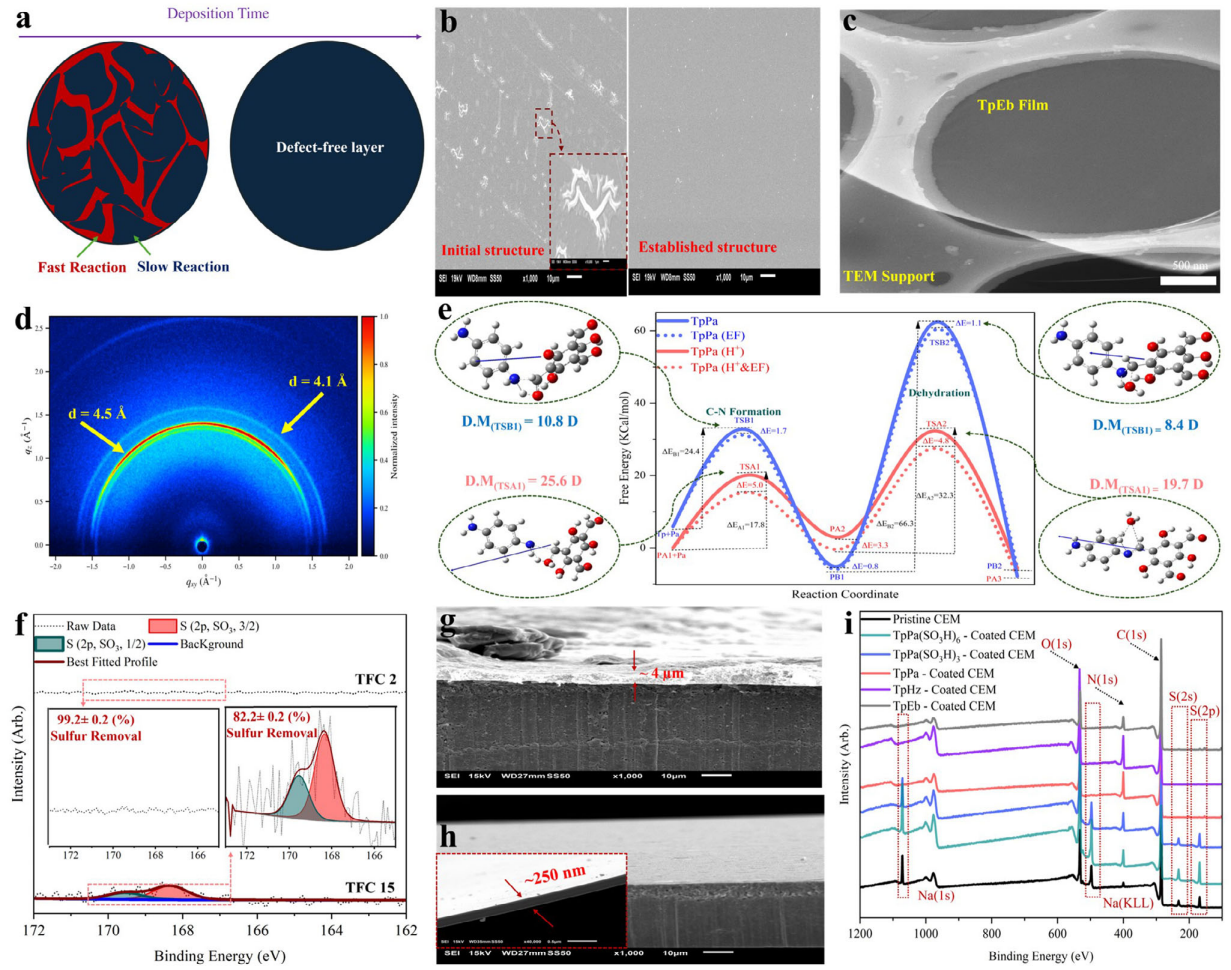
Defect‐free TFC membranes. (a) Schematic illustration of the proposed self‐healing and self‐regulating mechanism for the fabrication of defect‐free COF‐coated TFC membranes. (b) SEM micrographs of the membrane surface after 2.0 h (TFC 1, left) and 8.0 h (TFC 2, right) of deposition; the inset shows the residual uncovered regions at 2.0 h at higher magnification. (c) TEM micrograph of the TpEb coating after 8.0 h. (d) GIWAXS pattern of the TpEb coating after 8 h; the labeled reflections correspond to interlayer spacings of 4.5 and 4.1 Å. (e) Free‐energy profiles for the TpPa COF formation pathway obtained from DFT calculations, in the absence and presence of H^+^ and of an applied electric field (EF); D.M. denotes dipole moment. (f) Deconvoluted XPS spectra of the sulfur (S 2p) region arising from the CEM support beneath TpEb layers deposited under DC (TFC 15) and AC (TFC 2) electric fields. (g, h) Cross sectional SEM images of TpEb‐based TFC membranes fabricated under DC (g, TFC 15) and AC (h, TFC 2) electric fields; the inset in (h) shows the ∼250 nm selective layer at higher magnification. (i) XPS survey spectra of the COF‐coated membranes prepared in this study.

To overcome these challenges, the proposed fabrication approach relies on a self‐regulated local pH adjustment mechanism achieved through water‐splitting reactions at the electrode interfaces. A controlled amount of water is introduced as a co‐solvent, enabling the in situ generation of hydronium ions directly adjacent to the CEMs. The applied voltage, which is set above the thermodynamic threshold for water splitting, induces the electrolysis of water, generating H_3_O^+^ and OH^−^ ions at the anode and cathode, respectively (Figure 1c). The CEMs are selectively permeable to hydronium ions, a fraction of which migrates through the membrane under the applied electric field, enriching the monomer‐accumulated zone at the membrane‐solution interface. This process establishes a localized boundary layer, approximately 100 µm thick (Figure S13), with a pH lower than that of the bulk solution (Figure 1d–i). Within this acidic microenvironment, hydronium ions act as an in situ‐generated and green catalyst, accelerating the interfacial condensation between monomers and facilitating COF nucleation directly at the CEM surface rather than in the bulk solution. This water‐splitting‐assisted pH control mechanism thereby ensures spatial confinement of the polymerization reaction, promoting uniform COF film growth at the membrane surface; while suppressing unwanted particle formation in the bulk reaction medium.

### 2.3 Dynamic Electric‐Field‐Driven Self‐Healing of COF Layers

It has been well established that the local electric field intensity within the electrical double layer (EDL) can reach values as high as 0.1–1.0 V nm^−1^, thereby exerting a strong influence on reaction energetics and interfacial kinetics [23, 24, 25]. In the present system, the initial accumulation of charged COF‐forming monomers at the surface of the sulfonated CEMs can generate localized interfacial electric fields substantially higher than the externally applied macroscopic field. Consequently, the COF formation process is governed not only by the electrophoretic migration of charged monomers but also by the field‐induced modulation of reaction energetics within the monomer‐enriched interfacial zone. Such strong local fields are known to alter the stability of reaction intermediates and transition states, thereby affecting the overall kinetics of interfacial condensation reactions [26]. DFT simulations (Figure 2e) for a representative imine condensation between Tp and Pa monomers indicate that the presence of an electric field stabilizes both key transition states along the reaction pathway. Notably, this stabilization is more pronounced in the hydronium‐assisted pathway (of about 5.0 kcal mol^−1^ in the catalyzed path versus 1.7 kcal mol^−1^ in the non‐catalyzed path), which can be attributed to the larger dipole moments of the charged intermediates that experience greater polarization under the electric field. This finding supports the experimental observation of accelerated COF formation in the pH‐regulated, monomer‐enriched region adjacent to the CEM surface; conditions that are highly favorable for interfacial polymerization [21, 22].

In addition, the application of an electric field promotes the formation of defect‐free COF layers through a self‐healing and self‐regulating feedback mechanism (Figures 2a and S37; Discussion S2.6). As the reaction proceeds, the partial coverage of the CEM surface leads to two distinct regions with markedly different local reaction rates of COF nuclei: the newly formed patches act as a resistive barrier to both the electric field and hydronium ion transport, thus slowing further condensation reaction. Conversely, at grain boundaries, microcracks, and uncovered sites, the local field intensity and hydronium ion concentration remain elevated, driving faster reaction rates in these regions. This dynamic contrast establishes a self‐regulating feedback loop in which COF growth is selectively promoted at defective sites until a uniform and continuous film is achieved, as evidenced by the comparison of an incomplete coverage of the substrate membrane in 2.0 h and complete coverage in 8.0 h (Figure 2b,c). Equally important, the grazing‐incidence wide‐angle x‐ray scattering (GIWAXS) pattern (Figure 2d) shows a set of discrete, well‐defined diffraction rings, mainly at *q* ≈ 1.40 and 1.55 Å^−1^ (*d* = 4.5 and 4.1 Å, respectively), whose appearance as narrow rings of near‐uniform azimuthal intensity indicates polycrystalline domains of ordered material rather than an amorphous network, consistent with the crystalline stacking reflections reported for Tp‐based COF membranes [4, 27, 28]. As a result, the system naturally evolves toward homogeneous, crystalline, and defect‐free coverage of the CEM surface without the need for external correction or post‐treatment [14].

A key feature of the proposed fabrication method for COF‐based TFC membranes is the use of an alternating current (AC) electric field instead of direct current (DC). The AC field offers advantages that address several challenges: first, it significantly reduces the likelihood of hydrogen and oxygen gas bubble formation, and prevents the buildup of extreme acidic or basic conditions near the electrode surfaces. In contrast, under DC operation, water‐splitting products (H_2_, O_2_, and OH^−^ ions) tend to accumulate between the electrode and the adjacent CEM, as the membrane selectively allows hydronium ions passage but restricts the other water‐splitting products. This accumulation produces trapped gas bubbles, which compromise film formation in two ways: first, by exerting mechanical stress on the forming coating, increasing the likelihood of crack initiation and propagation; and second, by locally obstructing the transport of hydronium ions to the monomer‐enriched region, since the non‐conductive gas phase interrupts ionic delivery at the sites it occupies. By periodically reversing the field polarity, continuous single‐species accumulation at either interface is interrupted, allowing previously formed bubbles to detach and disperse and thereby limiting the peak gas hold‐up that drives crack formation under DC operation (Discussion S2.7 and Figure S15).

Second, AC operation enhances the structural quality of the resulting COF films, as it promotes redispersion or detachment of weakly bound monomeric (or oligomeric) aggregates and imperfect COF networks that may have prematurely precipitated onto the membrane surface [29]. These transient structures are then redistributed in the bulk solution and can reorganize during subsequent half‐cycles, yielding denser, more homogeneous, and defect‐free COF coatings. The improved coating quality under AC is supported by XPS, SEM, and AFM analyses. The sulfur signal originating from the underlying CEM support becomes nearly undetectable in the AC‐deposited membrane (Figure 2f,i, TFC 2 in Table S7), corresponding to a 99.2% ± 0.2% reduction in the detectable sulfur content, indicating a continuous, near‐complete coverage of the support. In contrast, the membrane prepared under DC conditions exhibits only an 82.2% ± 0.2% reduction in the sulfur content, indicating incomplete COF coverage. Complementary cross sectional SEM and AFM (Figures 2g,h andS11) further show that DC‐deposited films are substantially thicker (∼4.0 µm), rougher (*R*_q_∼ 60.0 nm), and contain microdefects. By comparison, AC‐deposited membranes are markedly thinner (∼0.25 µm), smoother (*R*_q_∼2.5 nm), and demonstrate uniform surface coverage.

Further comparison of AC‐ and DC‐deposited membranes (Figures S14 and S15a) and the versatility of the method, demonstrated with five monomers of distinct charge characteristics under different fabrication conditions, are presented in Figures S18–S22 and S26–S34. The underlying formation mechanism was elucidated through a combined experimental and computational approach and is discussed in Discussions 2.1–2.4 and 2.6–2.7 (Figures S6–S13 and Movies S1–S3).

### 2.4 Precise Monovalent‐Ion Sieving

The ion permeation performance of the prepared membranes was evaluated by combining electrochemical measurements with molecular dynamics (MD) simulations. Comparing the current–voltage (*I*–*V*) characteristics of the pristine CEM and TpEb‐coated CEM (TFC 2) (Figure 3a,b) reveals that introduction of an ultrathin, defect‐free, positively charged COF layer fundamentally alters ion transport behavior. While the K^+^ permeation current and effective conductance remain largely unchanged upon COF coating, Na^+^ transport is moderately suppressed. In sharp contrast, the currents of Li^+^ and Mg^2+^ are nearly completely eliminated across the applied voltage window, demonstrating pronounced ion‐discriminating transport governed by a combination of ion valence, charge density, and hydration characteristics rather than hydrated size alone.

**FIGURE 3:**
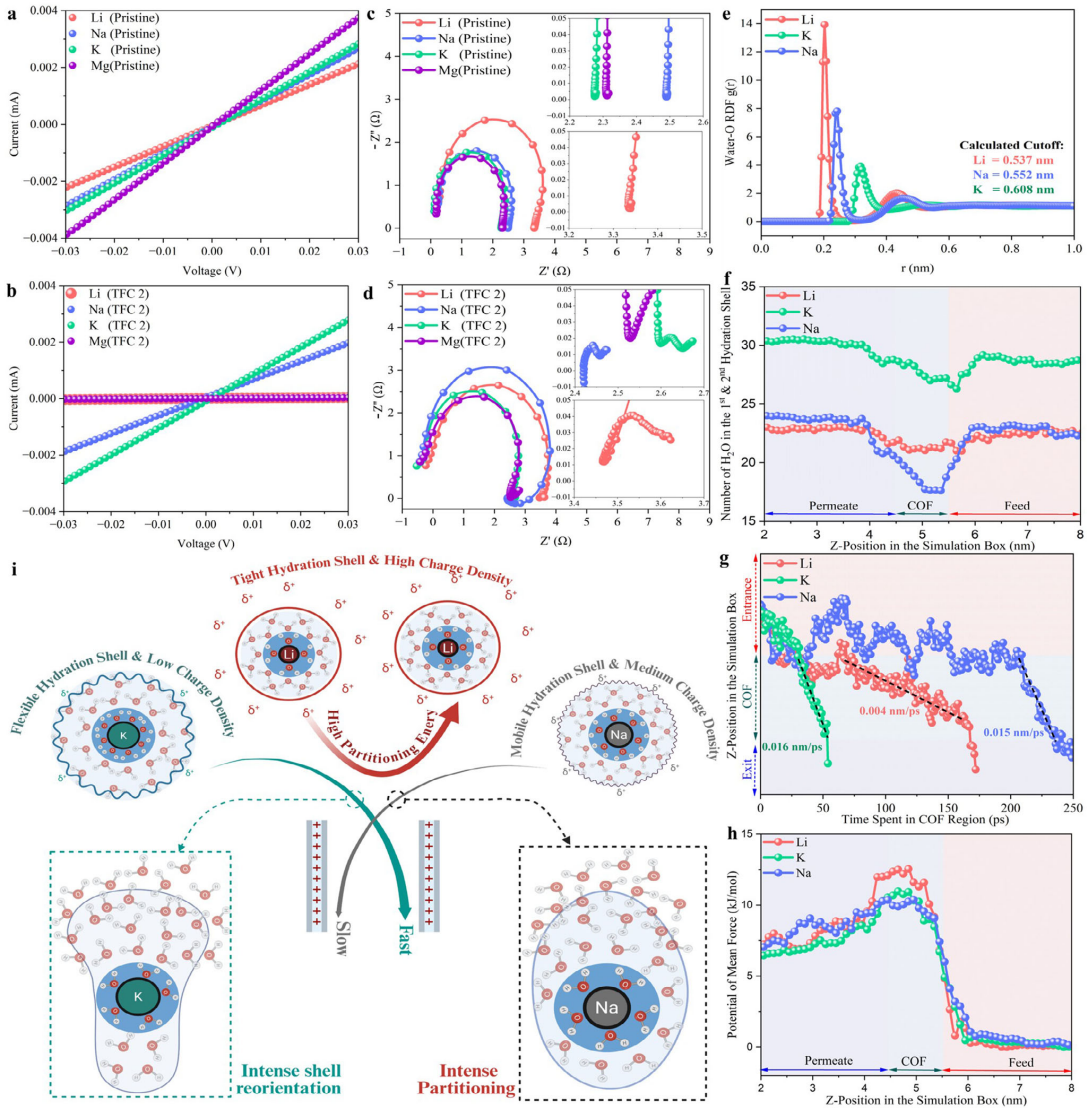
Ion separation mechanism. (a, b) *I*–*V* characteristics of the pristine and TpEb‐coated CEM (TFC 2). (c, d) Nyquist plots obtained from electrochemical impedance spectroscopy (EIS) for the pristine and TpEb‐coated (TFC 2) CEMs; inset panels show magnified views of the low‐frequency tails. (e–h) Molecular dynamics (MD) simulation results showing (e) radial distribution functions (RDFs) of the investigated ions, (f) the number of water molecules in the ions’ hydration shells as a function of their position in the simulation box, (g) ion positions along the z‐direction as a function of simulation time (with slopes indicating ion velocities in the z‐direction), and (h) potentials of mean force (PMFs) of the ions as a function of their position in the simulation box. (i) Schematic illustration of the proposed ion‐discrimination mechanism through the COF channel (created with BioRender.com).

Electrochemical impedance spectroscopy (EIS) provides further insight into the origin of this selectivity. Nyquist plots of the pristine and TFC 2 membranes (Figure 3c,d) display distinct impedance signatures corresponding to ion‐dependent transport mechanisms. Upon COF deposition, an additional resistance component emerges due to the electrostatic repulsion between the positively charged layer and the permeating ions. The high‐ and intermediate‐frequency semicircles are attributed to interfacial charge‐transfer resistance, while features appearing at low frequencies reflect mass‐transport and diffusional processes within the confined COF pores [30, 31, 32]. For Mg^2+^, a pronounced low‐frequency tail with an approximately 45° slope emerges, characteristic of a Warburg‐type diffusion impedance, indicating that ion transport is dominated by diffusion‐controlled resistance under strong electrostatic exclusion (Figure S23) at both the COF entrance and within the pore interior [33, 34]. In contrast, monovalent ions exhibit qualitatively different low‐frequency behavior. For Na^+^, the appearance of a positive imaginary impedance at low frequencies (inset graph in Figure 3d) corresponds to an inductive loop, commonly associated with delayed diffusional relaxation and ion accumulation‐depletion dynamics in confined and charged systems [35, 36]. Meanwhile, Li^+^ displays a prominent second semicircle at low frequencies, indicative of an additional transport time constant arising from severe diffusion resistance within the COF layer itself, a phenomenon frequently reported for ion transport through coated or nanoporous membranes where dehydration and confinement effects are the transport‐limiting factors [37, 38, 39].

MD simulations provide a molecular‐level explanation for these experimental observations by correlating ion hydration structure, dehydration energetics, and dynamics along the permeation pathway (Figure 3e–h). The ion‐water radial distribution functions (RDFs) show that K^+^ is coordinated by approximately 30.0 water molecules in the bulk region and loses only 2.0–3.0 water molecules upon entering the COF pore. In contrast, Na^+^ undergoes substantial dehydration, losing more than 7.0 water molecules near the pore entrance, while Li^+^ retains nearly its full hydration shell. The extracted hydration cutoffs (Li^+^ ≈ 0.537 nm, Na^+^ ≈ 0.552 nm, K^+^ ≈ 0.608 nm) further confirm that K^+^ possesses the most weakly bound and spatially diffuse hydration shell, evidenced by its broader RDF peaks [40]. Consequently, despite having the largest hydrated radius, K^+^ exhibits a highly labile hydration shell that can readily reorganize and deform under nanoscale confinement [41], resulting in a comparatively low energetic penalty for pore entry and transport (Figure 3i). By contrast, the hydration shells of Na^+^ and especially Li^+^ are significantly more rigid, reflected by sharper RDF peaks and stronger ion‐water interactions. Consequently, Na^+^ experiences a substantial dehydration penalty at the pore entrance, leading to delayed entry into the COF pores (inductive loop in Figure 3d). Li^+^ faces an even more severe limitation: partial dehydration is energetically unfavorable (Δ*G*_dehydration_ (kJ mol^−1^) ∼ Li^+^(−519.0)> Na^+^(−406.0)> K^+^(−322.0)) due to its tightly bound hydration shell, while simultaneous transport of this rigid shell through the positively charged COF pore results in enhanced frictional resistance and electrostatic repulsion arising from the high charge density of Li^+^ [42, 43]. These hydration‐controlled effects are directly reflected in the ion dynamics and free‐energy landscape. Trajectory analysis (Figure 3g) reveals significantly reduced axial mobility for Li^+^ within the COF region (0.004 nm ps^−1^) compared to Na^+^ and K^+^ (0.015 and 0.016 nm ps^−1^, respectively), while potential of mean force (PMF) profiles (Figure 3h) show the highest free‐energy barrier for Li^+^ permeation within the COF layers, resulting in a very low commitment probability at the pore entrance (Figure S24) and severely hindered transport within COF, whereas Na^+^ transport is primarily limited by dehydration at the entrance. In contrast, K^+^ traverses the pore efficiently due to facile hydration‐shell reorganization and lower transport resistance. These results demonstrate that ion selectivity is governed by the rigidity and deformability of the hydration shell of ions, and energetic cost of hydration‐shell restructuring under electrostatic confinement, also supported by experimental trends [44, 45]. The presence of defective structures creates shortcut transport pathways that eliminate both the dehydration‐induced transport delay and the hydration‐shell reorientation required at the pore entrance, thereby suppressing the ion selectivity mechanism (see Discussion S2.5 and Figure S36).

The ion separation performance of the membranes was further evaluated in electrodialysis (ED) with simulated mixed‐ion feeds. First, binary mixtures consisting of Mg^2+^ and a monovalent cation M^+^ (M = Li, Na, or K) were investigated independently. For the optimized TFC membrane (TFC 2), the concentration of Mg^2+^ in the receiving compartment remained below the ICP‐OES detection limit (1.0–2.0 ppb) throughout, corresponding to a permselectivity lower bound of > 10^5^ for the separation of Li^+^, Na^+^, and K^+^ from Mg^2+^‐rich feeds (Figure 4a–c), representing an unprecedented level of ion discrimination approaching ultra‐precision separation. Essentially all of the M^+^ was extracted from the feed within 2 h while Mg^2+^ transport remained undetectable, with high ion fluxes of approximately 0.04 ± 0.01, 0.12 ± 0.02, and 0.14 ± 0.02 mol m^−2^ h^−1^ for Li^+^, Na^+^, and K^+^, respectively, which are substantially higher than those of previously reported COF‐based, polymeric or mixed‐matrix membranes (Figure 4i).

**FIGURE 4:**
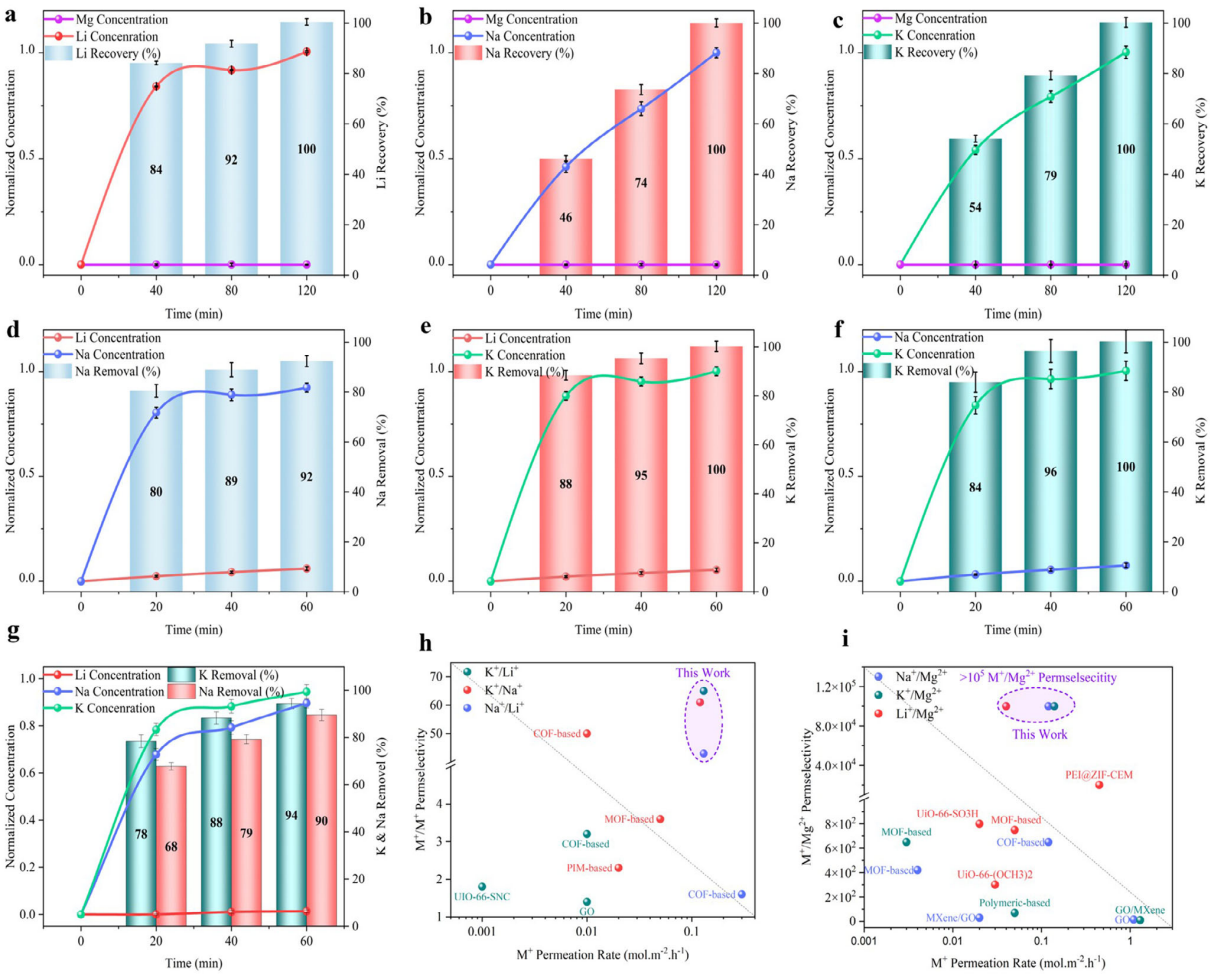
Ultra‐precise monovalent ion sieving. (a–c) Electrodialysis (ED) separation performance of the TpEb‐coated CEM (TFC 2) for the recovery of Li^+^, Na^+^, and K^+^ from LiCl/MgCl_2_, NaCl/MgCl_2_, and KCl/MgCl_2_ feed solutions, respectively. Solid lines indicate ion concentration in concentrate compartment. Feed compositions of MgCl_2_ (45.0 mM)/MCl (5.0 mM), where M = Li^+^, Na^+^, or K^+^. ED operating conditions: constant applied voltage of 1.5 V, initial current of approximately 2.0 mA, and operation time of 120.0 min. (d–f) ED separation performance of TFC 2 for the removal of trace amounts of Na^+^ and K^+^ from LiCl/NaCl, LiCl/KCl, and KCl/NaCl binary feed solutions, respectively. Feed compositions of M_1_Cl (45 mM)/M_2_Cl (5 mM), where M_1_ = Li^+^ or Na^+^ and M_2_ = Na^+^ or K^+^. (g) ED separation performance of TFC 2 for the removal of trace Na^+^ and K^+^ ions from a ternary LiCl (45 mM)/NaCl(5 mM)/KCl(5 mM) feed solution. ED operating conditions for d‐g: constant applied voltage of 1.0 V, initial current of approximately 1.0 mA, and operation time of 60.0 min. (h) Comparison of permselectivity and permeation rates of monovalent ions obtained using TFC 2 with literature‐reported values. (i) Comparison of permselectivity and permeation rates of monovalent ions (Li^+^, Na^+^, and K^+^) over Mg^2+^ for TFC 2 with values reported in the literature.

Particularly, TFC 2 also exhibits outstanding separation performance for mono/monovalent ion mixtures. Near‐complete removal of K^+^ was achieved from both Li^+^/K^+^ and Na^+^/K^+^ feeds, while 92.4 ± 1.6 mol% of Na^+^ was removed from a Li^+^/Na^+^ feed in only 1 h (Figure 4d–f). The permselectivity values are approximately 65.0 ± 3.2 for K^+^/Li^+^, 61.0 ± 2.6 for K^+^/Na^+^, and 43.0 ± 3.6 for Na^+^/Li^+^, with target‐ion fluxes of ∼0.13 ± 0.01 mol m^−2^ h^−1^ for K^+^ and ∼0.12 ± 0.01 mol m^−2^ h^−1^ for Na^+^, significantly exceeding those of reported polymeric and mixed‐matrix membranes (Figures 4h and S25). Similar performance (K^+^/Li^+^ and Na^+^/Li^+^ permselectivity of about 52.0 ± 3.3 and 41 ± 3.5, respectively) was reached with a ternary feed (Figure 4g) confirming that the durable and defect‐free TpEb‐coated TFC membrane enables high precision and ultra‐fast ion separation, even from feeds with extreme concentration asymmetry and in repeated tests (Figure S35).

## 3 Conclusions

Precise and rapid sieving of monovalent ions from mixed solutions remains a ubiquitous challenge in membrane science, owing to the difficulty of achieving defect‐free, ultrathin selective layers at the molecular level. We have introduced a green fabrication paradigm for defect‐free, durable COF‐based TFC membranes that combines dynamic electrophoretic assembly with in situ, water‐splitting‐induced pH regulation at the film‐forming interface. The method eliminates the need for an externally added acid catalyst, for toxic organic solvents, and for a conductive substrate, all of which are required by existing approaches, thereby removing key process constraints and providing a versatile platform for the reproducible fabrication of high‐performance films.

Beyond breaking the classical permeability‐selectivity trade‐off in monovalent ion sieving, the approach offers a route toward scalable manufacturing that is not readily accessible to conventional fabrication methods, once the reaction kinetics, support membrane, and fabrication device are optimized. Moreover, by tuning the applied field parameters and monomer composition, the method can be extended to other TFC membrane chemistries and adapted to construct thin films with multilayer architectures, paving the way toward single‐solute‐selective membranes for a broad range of applications.

## 4 Experimental Section

### 4.1 Materials and Methods

2,5‐Diaminobenzene‐1,4‐disulfonic acid (Pa(SO_3_H)_2_; ≈97.0%), p‐phenylenediamine (Pa; ≈98.0%), hydrazine solution (Hz; 35.0 wt% in water), ethidium bromide (Eb; ≈95%), 2,4,6‐trihydroxybenzene‐1,3,5‐tricarbaldehyde (Tp; ≈95.0%), octanoic acid (≥98.0%), potassium chloride (KCl; ≥99.0%), lithium chloride (LiCl; ≥99.0%), sodium chloride (NaCl; ≥99.0%), magnesium chloride (MgCl_2_; ≥98.0%), and sodium sulfate (Na_2_SO_4_; ≥99.0%) were purchased from Sigma‐Aldrich and used as received.

COF‐based TFC membranes were fabricated in a custom‐built cell in which a PTFE reaction chamber was sandwiched between two platinum‐coated titanium electrodes, each faced with a pre‐washed cation‐exchange membrane. For a typical batch, 0.15 mmol of the diamine (Pa; Pa(SO_3_H), Pa(SO_3_H)_2_, Hz, and Eb) monomer (in 7.5 mL DI water, diluted to 50 mL with ethanol) and 0.1 mmol of Tp (in 50 mL ethanol) were introduced into the chamber. An alternating potential of defined amplitude (2.0, 10.0, or 20.0 V peak‐to‐peak), waveform (square, sinusoidal, or ramp), and frequency (0.001, 1.0, or 1000 Hz) was applied for a set duration (2.0, 8.0, or 24.0 h) to drive interfacial COF formation. Full parameters and the complete TFC 1–18 series are given in the Supporting Information.

### 4.2 Characterization

Membrane chemistry, morphology, and structure were characterized by FTIR (PerkinElmer 100), XPS (Kratos Axis Supra, Al K_α_), SEM (Philips XL30 FEG), AFM (Bruker Dimension 3100D), solid‐state ^13^C CP/MAS NMR (Bruker Avance, 11.7 T), zeta‐potential analysis (Anton Paar SurPASS‐3), GIWAXS (Xenocs Xeuss 2.0, Cu K_α_), and TEM (JEOL JEM‐F200, 200 kV). For TEM, the delaminated TpEb layer was floated onto water, lifted onto a carbon‐coated copper grid, and dried under vacuum. Full instrument settings and sample‐preparation protocols are provided in the Supporting Information.

### 4.3 Ion Separation Performance

Ion transport was assessed by *I*–*V* and electrochemical impedance spectroscopy (EIS) measurements (Autolab PGSTAT302N; 100 kHz–0.1 Hz, 0.1 V perturbation) using 0.1 M LiCl, NaCl, or KCl feeds. Separation performance was evaluated in a custom four‐compartment electrodialysis (ED) cell (effective membrane area ≈ 19.6 cm^2^) with binary and ternary mixed‐ion feeds. Ion concentrations in the feed and receiving compartments were quantified by inductively coupled plasma optical emission spectrometry (ICP‐OES; PerkinElmer Optima 8300) using beryllium as an internal standard, with the instrument calibrated for each batch; the detection limit for the analyte ions was 1.0–2.0 ppb. Full cell configuration, ED protocols, and the uncertainty analysis are detailed in the Supporting Information.

### 4.4 Simulations

DFT calculations were performed with Dmol^3^ (Materials Studio 2024) using the PBE/GGA functional, a DNP basis set, and Grimme DFT‐D3 dispersion; transition states were located by LST/QST and confirmed by frequency analysis, with single‐point energies refined at the ωB97X‐D/def2‐TZVPP level. Molecular dynamics simulations (GROMACS 2023, GAFF2 force field, RESP charges) modeled ion transport through a three‐layer TpEb framework in 1.0 M LiCl, NaCl, or KCl. Computational fluid dynamics simulations (COMSOL Multiphysics 6.3) coupled the Navier‐Stokes, Nernst‐Planck, and charge‐conservation equations under DC (steady‐state) and AC (transient) conditions. Full computational details are given in the Supporting Information.

## Author contributions

Conceptualization: M.H.J. and X.Y.; Methodology: M.H.J., X.Y., and R.A.; Investigation: all authors; Visualization: M.H.J., X.Y., and R.A.; Funding acquisition and project administration: X.Y.; Supervision: X.Y.; Writing (original draft): M.H.J.; Writing (review & editing): all authors.

## Conflicts of Interest

A patent application related to this work has been filed (applicant: KU Leuven; inventors: Mohammad Hossein Jandaghian and Xing Yang; PCT application number: PCT/EP2026/073606; status: priority application filed). The subject matter concerns methods for the fabrication of thin‐film composite (TFC) membranes and their application in monovalent/monovalent and monovalent/divalent ion separations.

## Disclaimer

Views and opinions expressed are those of the authors only and do not necessarily reflect those of the European Union or the European Research Council Executive Agency. Neither the European Union nor the granting authority can be held responsible for them.

## Supporting Information

The Supporting Information file includes materials and methods, supporting discussions, Figures S1–S37, Tables S1–S8, and Movies S1–S3.

## Supporting information




**Supporting file 1**: adma74934‐sup‐0001‐SuppMat.pdf.

**Supporting file 2**: adma74934‐sup‐0002‐MovieS1.mp4.

**Supporting file 3**: adma74934‐sup‐0003‐MovieS2.mp4.

**Supporting file 4**: adma74934‐sup‐0004‐MovieS3.mp4.

## Data Availability

The data that support the findings of this study are available from the corresponding author upon reasonable request.
