# Supplementary material for: Dynamic Electrophoretic Assembly Coupled With Water‐Splitting‐Induced pH Control Enables Precision Ion Sieving Membranes
Source: Adv Mater. 2026 Sep 9;38(63):e74934. doi: 10.1002/adma.74934 (PMC13755646; doi:10.1002/adma.74934)
Supplement: Supplementary file 1 [file ADMA-38-e74934-s001.pdf]

Supplementary Information for

Dynamic Electrophoretic Assembly Coupled with Water-Splitting-Induced pH Control Enables Precision Ion Sieving Membranes

Mohammad Hossein Jandaghian[1], Petric Marc Ruya[1], Ipek Ozyurt[1], Ali Atyabi[1], Samuel Eyley[2], Wim Thielemans[2], Alexander Volodin[3], Riccardo Alessandri[4], and Xing Yang[1,*]

Contents

1. Materials and Methods 5
1.1. Materials 5
1.2. Methods 5
1.2.1. Fabrication Chamber 5
1.2.2. Synthetic Procedure 6
1.2.3. Characterization 7
1.2.4. Electrochemical impedance spectroscopy (EIS) and current-voltage (I-V) Measurements 10
1.2.5. Electrodialysis (ED) experiments 11
1.3. Simulations 13
1.3.1. Density Functional Theory (DFT) 13
1.3.2. Molecular Dynamics (MD) Simulations 14
1.3.3. Computational Fluid Dynamics (CFD) simulation 18
2. Supplementary Discussions 23
2.1. Effect of the voltage type on coating adhesion quality 23
2.2. Versatility of the developed fabrication method 24
2.3. Mechanistic Insight into the Electrophoretic Deposition of Neutral COFs 27
2.4. Effect of fabrication parameters on the ion separation performance 28
2.5. Effect of Defects on Ion Permeation Behavior Investigated by MD Simulations 29
2.6. The self-healing and self-regulating growth mechanism 30
2.7. Gas evolution, bubble dynamics, hydronium generation, the balance between acid supply and condensation kinetics, and the validity of the single-phase model under AC operation 32
3. Supplementary Figures 38
Figure S1. Custom-built transparent fabrication chamber. 38
Figure S2. Synthesized COFs. 39
Figure S3. Electrochemical cell. 40
Figure S4. Electrodialysis cell. 41
Figure S5. Simulated diffusion coefficients. 42
Figure S6. Definition of parameters used in CFD simulations. 43
Figure S7. Velocity distribution in the fabrication chamber. 44
Figure S8. Effect of electric field on the deprotonation of monomers. 45
Figure S9. Mechanism of imine-link formation catalyzed with protons. 46
Figure S10. Mechanism of imine-link formation without protons. 47
Figure S11. Effect of the type of electric field on the surface morphology of TFC membranes. 48

Figure S12. Effect of the addition of external organic acids. 49

Figure S13. Calculated pH profiles near the CEM in the steady-state and transient CFD simulations. 50

Figure S14. Effect of the type of electric field on the adhesion quality of COF films. 51

Figure S15. Effect of the type of electric field on the adhesion quality of COF films and bubble formation. 52

Figure S16. Effect of the duration of deposition on the surface morphology of TFC membranes. 53

Figure S17. Effect of fabrication parameters on the structural integrity of the TFC membranes. 54

Figure S18. Effect of the waveform on the surface morphology of TFC membranes. 55

Figure S19. Effect of the electric-field frequency on the surface morphology of TFC membranes. 56

Figure S20. Effect of the electric-field intensity on the surface morphology of TFC membranes. 57

Figure S21. FTIR evidence for COF formation and for complete surface coverage of the optimized membrane. 58

Figure S22. Presence of tautomeric equilibrium in the synthesized COFs. 59

Figure S23. Surface charge of synthesized COFs. 60

Figure S24. Feasibility of monovalent cation transport in the TpEb COF layers. 61

Figure S25. Comparison of separation performance. 62

Figure S26. Surface morphology and ED performance evaluation of the pristine CEM used as the support in this study. 63

Figure S27. Effect of the duration of deposition on the ionic conductance. 64

Figure S28. Effect of the duration of deposition on the separation performance. 65

Figure S29. Effect of the voltage intensity on the ionic conductance. 66

Figure S30. Effect of the voltage intensity on the separation performance. 67

Figure S31. Effect of the voltage frequency on the ionic conductance. 68

Figure S32. Effect of the voltage frequency on the separation performance. 69

Figure S33. Effect of the waveform on the ionic conductance. 70

Figure S34. Effect of the waveform on separation performance. 71

Figure S35. Stability assessment of the prepared membranes. 72

Figure S36. Molecular dynamics (MD) simulation results for the defective COF membrane. 73

Figure S37. Evolution of COF coating thickness with deposition time. 74

4. Supplementary Tables 75

Table S1. Specifications of the IEMs used in this work. 75

Table S2. Synthesized COF structures and the corresponding monomers. 76

Table S3. Synthesis parameters for the COF-deposited TFC membranes. 77
Table S4. Initial and boundary conditions used in CFD simulations. 78
Table S5. Dimensional and material properties used in CFD simulations................................ 79
Table S6. Applied electrical waveforms on positive and negative electrodes for AC CFD simulations (20 V case). 80
Table S7. Quantitative XPS analysis showing the surface sulfur signal attenuation of COF-based TFC membranes. 81
Table S8. The results of ion separation performance of all COF-based TFC membranes synthesized in this work. 82
5. Supplementary Movies 83
Movie 1. CFD results for ramp potential 83
Movie 2. CFD results for sinusoidal potential 83
Movie 3. CFD results for square potential 83
6. References 84

1. Materials and Methods

1.1. Materials

2,5-Diaminobenzenesulfonic acid (≥97.0%), 2,5-Diaminobenzene-1,4-disulfonic acid (≈97%), p-phenylenediamine (≈98%), hydrazine solution (35 wt% in water), ethidium bromide (≈95%), 2,4,6-trihydroxybenzene-1,3,5-tricarbaldehyde (≈95%), octanoic acid (≥98%), potassium chloride (≥99.0%), lithium chloride (≥99.0%), sodium chloride (≥99.0%), magnesium chloride (≥98%), and sodium sulfate (≥99.0%) were purchased from Sigma-Aldrich and used as received without further purification. Ethanol (≥99.8%) was obtained from Acros Organics. The specifications of the cation-exchange membranes (CEMs) used in both the synthesis and electrochemical performance experiments, and anion-exchange membranes (AEMs) used in ED experiments are summarized in Table S1.

1.2. Methods

1.2.1. Fabrication Chamber

A custom-built membrane fabrication setup was employed in this work, and its essential components are illustrated in Figures 1 a-b. The core of the system consisted of a PTFE block containing a circular cavity that served as the reaction chamber. This block, equipped with a magnetic stir bar, was sandwiched between two flat platinum-coated titanium electrodes and positioned on a magnetic stirrer. The applied voltage, of a desired waveform, frequency, and amplitude, was generated using a laboratory signal generator, adjusted to the required intensity through a high-voltage amplifier, and subsequently delivered to the electrodes. In addition to these main components, two commercially available cation-exchange membranes (CEMs) were placed directly on the surfaces of the electrodes and sandwiched between the PTFE block and the

electrodes. These membranes served as structural supports for the formation of the COF-based TFC membranes.

The electrodes used in the system were flat corrosion-resistant platinum-coated titanium plates (12 × 12 cm), fabricated by Umicore® and employed without further surface treatment. A Siglent SDG2042X® function generator, capable of producing various waveform signals over a wide range of voltages and frequencies, was used as the electrical signal source. The generator output was connected to an A400 High Voltage Linear Amplifier, made by Pendulum®, to achieve the desired potential range. The amplifier output was then directly connected to the electrodes using shielded conductive wires.

To enable real-time optical monitoring of the processes occurring inside the fabrication chamber, an identical chamber was constructed using polymethyl methacrylate (PMMA) in place of PTFE. The use of PMMA made the chamber optically transparent, allowing in situ visualization and photography of the ongoing phenomena during membrane fabrication without altering the chamber geometry or operating conditions (Figure S1).

1.2.2. Synthetic Procedure

All chemicals were used as received without any further purification. Chemicals that were sensitive to air or moisture were handled under a dry nitrogen atmosphere. All glassware, electrodes, and fabrication chambers were thoroughly cleaned by rinsing three times with deionized (DI) water and subsequently dried before use. In a typical fabrication procedure, two CEMs were cut into square pieces ($7.5 \times 7.5\ cm^2$) and repeatedly washed with DI water to remove any surface-adsorbed impurities. Each CEM was then carefully attached to the surface of a platinum-coated titanium electrode while both were immersed in DI water. Performing this step under water ensured complete removal of air bubbles between the electrode surface and the CEM, providing uniform interfacial contact. After both CEMs were properly positioned, the membrane-

electrode assemblies were sandwiched between the PTFE chamber blocks and secured using butterfly bolts, forming a leak-free and stable electrochemical cell for the subsequent fabrication process.

For synthesizing COF-based TFC membranes, a required amount of the diamine-based monomer was dissolved in 7.5 mL of deionized (DI) water and subsequently diluted with absolute ethanol to a final volume of 50 mL. In a separate reagent bottle, 50 mL of absolute ethanol was used to dissolve the aldehyde-based monomer, and the resulting mixture was stirred overnight to ensure complete dissolution. Following complete preparation of both monomer solutions, they were simultaneously introduced into the interelectrode space of the assembled fabrication chamber. An external potential with a predefined voltage, waveform, and frequency was then applied between the electrodes to initiate the COF formation reaction. The deposition was allowed to proceed for a specified reaction duration, depending on the experimental design. Upon completion of the reaction, the signal generator was turned off and the system was disassembled by releasing the butterfly bolts. The coated CEMs were then carefully detached from the electrodes and immediately immersed in DI water. To remove any unreacted monomers or weakly adsorbed species, the resulting COF-based TFC membranes were rinsed repeatedly with copious amounts of DI water. The cleaned membranes were stored in DI water until further characterization or performance testing. The monomers, corresponding COF structures, and synthesis parameters used in this study are summarized in Tables S2 and S3 and Figure S2.

1.2.3. Characterization

Fourier Transform Infrared (FTIR): FTIR spectra of the membranes were recorded using a PerkinElmer 100 FTIR spectrometer to analyze the chemical composition of the uncoated and COF-based TFC membranes. Spectra were collected over the range 4000-650 cm^{-1} with a resolution of 4.0 cm^{-1}.

X-ray Photoelectron Spectroscopy (XPS): The surface elemental composition was analyzed by XPS using a Kratos Axis Supra spectrometer equipped with a monochromated Al K_α X-ray source (1486.7 eV). Measurements were performed at an X-ray acceleration voltage of 15.0 kV and an emission current of 8.0-10.0 mA. The instrument was operated in hybrid magnetic/electrostatic lens mode. Survey spectra were acquired at a pass energy of 160.0 eV, while high-resolution spectra were recorded at 20.0 eV. A low energy electron gun was used for charge neutralization during acquisition. All spectra were collected at normal emission and processed using CasaXPS software. The binding energy scale was referenced to aromatic carbon at 284.7 eV for high-resolution spectra. Background subtraction was carried out using a two-parameter Tougaard function, and elemental compositions were determined from peak areas corrected by Scofield sensitivity factors, with further adjustments for photoelectron angular distribution and effective attenuation length[1]. The instrumental transmission was corrected using the NPL transmission function[2]. The reported atomic percentages represent equivalent homogeneous surface compositions and do not account for nanoscale or depth-dependent chemical inhomogeneity.

Scanning Electron Microscopy (SEM): Surface and cross-sectional morphologies were examined by SEM using a Philips XL30 FEG (Netherlands) operated at 15.0 and 19.0 kV. Membrane cross-sections were prepared by fracturing the samples in liquid nitrogen, followed by gold sputter-coating to enhance conductivity.

Atomic Force Microscopy (AFM): AFM measurements were performed under ambient conditions using a Dimension 3100D AFM (Bruker, USA) over both 5.0 × 5.0 and 1.0 × 1.0 μm^2 area to characterize surface topography. AFM images were flattened using a first-order polynomial, and the root-mean-square (RMS) roughness was calculated in accordance with ISO 25178-2. The deposited film thickness was measured using the AFM scratch-and-scan method: the film was

scratched with a hard plastic knife, then a scan was performed through the scratch, which was used to determine the thickness of the film via a cross-section profile.

Solid Nuclear Magnetic Resonance: ^{13}C solid-state CP/MAS experiments were performed on an 11.7 T magnet (500 MHz proton Larmor frequency) using a 4.0 mm Chemagnetics probe and a Bruker Avance I spectrometer. Samples were spun at 15.0 kHz. The proton 90.0° pulse length was 6.8 µs, and the cross-polarization contact time was 3.0 ms. The spectral width was set to 40.0 kHz, and 3072 points were acquired in the free-induction decay. A repetition delay of 8.0 s was used, and 4096 scans were accumulated before Fourier transformation, with 100 Hz linear broadening and zero-filling to 32,768 points. Chemical shifts were referenced to adamantane. The total acquisition time for each sample was approximately 9.0 hours.

Zeta-potential Measurements: Zeta potential (ζ) measurements were performed using a SurPASS-3 electrokinetic analyzer (Anton Paar GmbH, Graz, Austria). Prior to each experiment, the samples were rinsed five times with 0.001 M KCl (Sigma Aldrich) to stabilize surface ion adsorption and ensure complete wetting. ζ-potential values were recorded as a function of pH in 0.001 M KCl, with pH adjusted by the instrument's automatic titration unit using 0.05 M HCl or 0.05 M NaOH. Streaming current data were collected and converted to ζ-values using the Helmholtz-Smoluchowski equation. The applied pressure settings were: upper pressure 400.0 mbar, lower pressure 150.0 mbar, and cutoff pressure 300.0 mbar. All measurements were performed in triplicate, and the reported values represent the averaged results. Electrolyte solutions were prepared using deionized water.

Grazing-incidence wide-angle X-ray scattering (GIWAXS): GIWAXS measurements were performed on a Xeuss 2.0 laboratory system (Xenocs, France) equipped with a Cu K_α microfocus source (λ=1.54189 Å) operated at 30.0 W. Samples were measured at a fixed incidence angle of 0.2 degree, and 2D patterns were collected on a Pilatus 3R 300K hybrid-pixel detector at a sample-

to-detector distance of 137.0 mm with an exposure time of 300.0 s. The detector images were calibrated in reciprocal space using the known beam center and sample-to-detector distance, and pixel coordinates were converted to the scattering vector q. 2D maps were reconstructed in (q_{xy}, q_z) coordinates and 1D intensity profiles extracted by azimuthal integration using a custom Python script.

Transmission Electron Microscopy (TEM): The morphology and continuity of the COF selective layer were examined by transmission electron microscopy using a JEOL JEM-F200 microscope operated at an accelerating voltage of 200.0 kV. For TEM analysis, the TpEb COF layer was transferred to a carbon-coated copper grid by floating the delaminated film onto the water surface and lifting it onto the grid, and dried under vacuum conditions prior to imaging.

1.2.4. Electrochemical impedance spectroscopy (EIS) and current-voltage (I-V) Measurements

The working principle of EIS involves the sequential application of sinusoidal alternating currents of known frequencies to an electrochemical system. The resulting voltage response, including the phase shift between current and voltage, is measured digitally at each frequency. From these data, the capacitance and conductance of the system can be determined. The overall electrical impedance (Z) is expressed as a complex quantity ($Z=Z_{real} - jZ_{imag}$), where Z_{real} represents the resistive component and Z_{imag} corresponds to the capacitive component of the membrane response[3–5].

EIS experiments in this work were conducted using a four-compartment, lab-fabricated electrochemical cell equipped with two working electrodes and two reference electrodes, as illustrated in Figure S3. Measurements were carried out using an Autolab PGSTAT302N potentiostat/galvanostat equipped with a Frequency Response Analyzer (FRA) module. The

impedance spectra were recorded over the frequency range of 100.0 kHz to 0.1 Hz, employing a sinusoidal perturbation amplitude of 0.1 V.

Similarly, the ionic transport properties of the fabricated membranes were evaluated by recording current-voltage (I-V) characteristics using the same lab-fabricated four-compartment electrochemical cell employed for EIS measurements (Figure S3). The I-V measurements were performed to compare the ion transport behavior across different membrane samples under identical experimental conditions.

Prior to each measurement, the assembled cell was equilibrated for 10.0 minutes to ensure steady-state conditions and to minimize transient effects related to ion redistribution or double-layer charging. The applied potential was incrementally varied within the desired range, and the corresponding current was recorded after stabilization at each voltage step [6]. The resulting I-V curves were subsequently analyzed to determine the ionic conductivity and transport resistance of the membranes.

For both EIS and I-V measurements, 0.1 M aqueous solutions of LiCl, NaCl, and KCl were used as the feed electrolytes, while a 0.2 M Na_2SO_4 solution served as the electrode rinsing solution to maintain ionic balance and minimize electrode polarization.

1.2.5. Electrodialysis (ED) experiments

ED experiments were conducted using a custom-built four-compartment electrochemical cell equipped with two electrodes, as illustrated in Figure S4. The effective surface area of each COF-based thin-film composite (TFC) membrane was ~ 19.6 cm^2, and the width of the working compartments was maintained at 5.0 cm. The outermost compartments served as electrode rinse chambers, each containing a recirculating 0.2 M Na_2SO_4 solution to maintain ionic balance and minimize electrode polarization.

The feed and receiving compartments were continuously stirred using IKA Nanostar overhead stirrers fitted with PTFE shafts to ensure homogeneous mixing. A 0.1 M HCl solution was used in the receiving compartment to promote ion migration and maintain charge balance. Unless stated otherwise, the feed solution consisted of 10.0 mol% of the target contaminant ion to be removed and 90.0 mol% of the ion intended for purification. Each compartment contained 100.0 mL of solution. During operation, samples (100.0 μL) were collected from both the feed and receiving compartments every 20.0 minutes from the start of the experiment.

Cation concentrations in the feed and receiving compartments were determined by the inductively coupled plasma optical emission spectrometry (ICP-OES) using a PerkinElmer Optima 8300 spectrometer. Where necessary, samples were diluted with distilled water to bring the maximum cation concentration within the calibrated range, and nitric acid was added to a final concentration of approximately 2.0 wt% to match the acid matrix of the standards. Beryllium was used as an internal standard to correct for instrumental drift and matrix effects. The instrument was calibrated for each batch of measurements.

All the separation experiments were performed using three independently fabricated membranes tested under identical conditions, and the permselectivity and ion-flux values are given as the mean of these three independent replicates ± one standard deviation.

The detection limit of the instrument for the ions relevant to the reported separations was 1.0-2.0 ppb. For the most selective membranes (TFC 2), the concentration of the excluded ion in the receiving compartment remained below this limit throughout the experiment. Such a measurement does not establish that the permeating flux is strictly zero; it establishes an upper bound on that flux, and correspondingly a lower bound on the permselectivity. Accordingly, where the excluded-ion concentration fell below the detection limit, the permeating flux was evaluated at the detection-limit concentration and the resulting permselectivity is reported as a lower bound (>) with the

governing detection limit stated, rather than as an infinite or exact value. The efficiency of contaminant removal from the feed solutions was calculated using the following equations:

$$\text{Permselectivity} = \frac{(C_{t,r} - C_{t,r0})/C_{t,f0}}{(C_{c,r} - C_{c,r0})/C_{c,f0}}$$

$$\text{Ion Removal/Recovery Percentage} = \frac{(C_{t,r} - C_{t,r0})}{C_{t,f0}} * 100$$

$$\text{Flux} = \frac{(C_{t,r} - C_{t,r0})}{s \,.\, t}$$

In these equations, $C_{t,r}$, $C_{t,r0}$ denote the concentrations of the targeted ion (to be removed) in the receiving chamber at the end and at the beginning of the ED experiment, respectively, while $C_{c,r}$, $C_{c,r0}$ correspond to the concentrations of the competing ion in the receiving chamber at the end and at the beginning of the experiment. Moreover, $C_{t,f0}$, $C_{c,f0}$ represent the initial concentrations of the targeted and competing ions in the feed chamber, respectively, while parameters s and t denote the effective membrane area and the duration of the ED experiment.

1.3. Simulations

1.3.1. Density Functional Theory (DFT)

DFT calculations were performed to obtain the optimized geometries, transition states (TS), and harmonic vibrational frequencies of all structures. These calculations were carried out using the DMol^3 module of the Materials Studio 2024 suite. The Perdew-Burke-Ernzerhof (PBE) functional within the generalized gradient approximation (GGA) framework was adopted to describe exchange-correlation effects[7]. A double numerical basis set with polarization functions (DNP) was employed for all atoms to ensure adequate accuracy. The core electrons were represented by effective core pseudopotentials (ECPs) developed by Dolg and Bergner, and long-range dispersion interactions were accounted for through Grimme's DFT-D3 semiempirical correction[8–10]. Solvent effects were included using the conductor-like screening model (COSMO) with a dielectric constant of 58.0, representing a mixture of 7.5% (v/v) water and 92.5% (v/v) ethanol[11].

Transition states between adjacent minima were initially located using the linear synchronous transit (LST) approach and subsequently refined by quadratic synchronous transit (QST) and conjugate gradient optimization. Frequency analysis was performed to verify that all optimized structures corresponded to local minima, and that TS structures contained a single imaginary mode along the reaction coordinate[12]. Finally, to refine the computed electronic energies, single-point calculations were performed using Gaussian 16 package with B3LYP hybrid functional and the def2-TZVPP basis set[13,14]. To investigate the effect of the electric field, a field of desired intensity was applied in all three X, Y, and Z directions using the F=(intensity ± direction) keyword in the Gaussian input command line.

1.3.2. Molecular Dynamics (MD) Simulations

For calculating diffusion coefficients of the COF-forming monomers, MD simulations were performed with the GROMACS 2023 package. The MD simulations were done to determine the diffusion coefficients (D_i) of the two monomer species, which were subsequently used as input parameters for the CFD simulations described in the following section. The two monomers studied were $Pa(SO_3H)_2$ (2,5-diaminobenzene-1,4-disulfonic acid), which carries a net negative charge, and Tp (2,4,6-trihydroxy-1,3,5-benzenetricarbaldehyde), which is neutral under the simulated conditions. The optimized geometries of both monomers, obtained from the DFT calculations described in the previous section, were used as the basis for constructing the molecular models. All bonded and non-bonded interactions (including bonds, angles, and dihedral terms) were parameterized using the General AMBER Force Field (GAFF2). Because atomic charges play a critical role in determining electrophoretic mobility under an applied electric field, accurate partial charges were obtained using the two-point restrained electrostatic potential (RESP) fitting procedure implemented in Multiwfn[15,16]. The resulting charge distributions were incorporated into the molecular topologies for subsequent MD simulations.

Prior to equilibration, each system underwent energy minimization using the conjugate gradient algorithm to remove steric clashes and optimize initial configurations. The minimization was terminated when the maximum force fell below 100.0 Kj mol^{-1} nm^{-1}, with an initial step size of 0.01 nm. The Verlet cutoff scheme was used, and periodic boundary conditions (PBCs) were applied in all three spatial directions. Electrostatic interactions were treated using Particle Mesh Ewald (PME) method with a 1.0 nm real-space cutoff, fourth-order interpolation, and a Fourier grid spacing of 0.12 nm. Lennard-Jones (LJ) interactions were truncated at 1.0 nm, and long-range dispersion corrections were applied by setting DispCorr = EnerPres, as required for GAFF-based simulations.

Following minimization, an annealing protocol was employed to thermally relax the systems and eliminate local energy traps. The V-rescale thermostat was used for temperature coupling ($\tau_t = 0.1$ ps) under NVT conditions, with the temperature cycled between 298.0 K and 600.0 K in a series of ten segments over a 2.0 ns trajectory[17]. The annealing schedule included three heating-cooling cycles to promote conformational sampling. The time step was 1.0 fs, and all bonds involving hydrogen atoms were constrained using the LINCS algorithm (order = 4).

After annealing, the systems were equilibrated under isothermal-isobaric (NPT) conditions for 25.0 ns using the C-rescale barostat ($\tau_p = 1.0$ ps, reference pressure = 1.0 bar, compressibility = 4.5×10^{-5} bar^{-1})[18]. Temperature was maintained at 298.15 K with the V-rescale thermostat. The Verlet neighbor search scheme was applied with a 1.0 nm cutoff and neighbor list updates every 10 steps.

Subsequent production simulations were performed under NVT conditions for 40.0 ns with a 1.0 fs integration time step, continuing from the equilibrated configurations to ensure smooth continuation. The Leapfrog algorithm was used to integrate the equations of motion. The system temperature was kept constant at 298.15 K using the V-rescale thermostat. Periodic boundary

conditions were applied in all three directions, and center-of-mass motion was removed every step. Long-range electrostatics were computed via the PME method (cutoff = 1.0 nm, grid spacing = 0.12 nm, PME order = 4, relative tolerance = 10^{-5}), while van der Waals interactions used a 1.0 nm cutoff with energy and pressure dispersion corrections applied. Output frequencies were set to 1.0 ps for log and energy files and 2.0 ps for trajectory frames.

To evaluate the influence of the applied electric field on monomer diffusion, a series of production simulations was performed under varying field intensities. In each case, a uniform electric field was applied along the z-axis. The diffusion coefficients (D_i) of both monomers were determined from the mean square displacement (MSD) as a function of time, using the Einstein relation[19]. MSD values were averaged over all equivalent monomer molecules, and the linear regime of the MSD curve was identified and fitted using a custom Python script to ensure accurate sampling of diffusive motion. The resulting diffusion coefficients were then plotted as a function of the applied electric field intensity (Figure S5). The relationship between D and E was fitted to a continuous function, and the corresponding diffusion coefficient at the target voltage level was subsequently used as input for the CFD simulations described in the following section.

MD simulations for evaluating the ion-selective transport behavior of the TpEb COF were performed for aqueous LiCl, NaCl, and KCl (1.0 M) electrolytes. These simulations were carried out using GROMACS 2023, following the same general workflow and numerical settings described in the previous subsection, except where noted below.

The TpEb COF membrane was modeled as a three-layer stack positioned at the center of the simulation cell. The space between the edges of the COF layers and the boundaries of the simulation box was filled with dummy atoms to block ion transport through peripheral shortcut pathways and thereby force the ions to pass exclusively through the intrinsic membrane pores. To investigate the effect of defects on ion separation performance, these dummy atoms were removed,

allowing ions to permeate through both the intrinsic membrane pores and the defective edge regions (see Supplementary Discussion 2.5 and Figure S36). All framework atoms were frozen during the simulations to maintain structural integrity while allowing ions and solvent molecules to move freely through the pores. For the alkali-metal cations (Li^+, Na^+, and K^+), Lennard-Jones parameters were adopted from literature sources [20–22]. Each system was first subjected to energy minimization using the steepest-descent algorithm, with convergence achieved when the maximum force dropped below 100.0 kJ mol^{-1} nm^{-1}. A 9-3 wall potential was defined at the top and bottom of the box to confine the electrolyte in the z direction, with periodic boundaries in the x and y directions only. Following minimization, the systems were equilibrated in the NPT ensemble for 10.0 ns with a 0.25 fs integration time step. Temperature was maintained at 298.15 K using the V-rescale thermostat (τ_t = 0.1 ps), and semi-isotropic pressure coupling was applied with the C-rescale barostat (τ_p = 2.0 ps, reference pressure = 1.0 bar in the plane of the membrane). Bond constraints were imposed on all hydrogen-containing bonds using the LINCS algorithm (order = 4).

Production simulations were then conducted under NVT conditions for 40.0 ns with a 0.5 fs time step, continuing from the equilibrated configurations. The temperature was held at 298.15 K using the V-rescale thermostat. Periodic boundary conditions were maintained in the x-y plane, and two soft repulsive walls (9-3 potential, density = 10.0 atoms nm^{-3}) were retained along the z direction to preserve the confinement geometry. van der Waals interactions were truncated at 1.0 nm, and long-range dispersion corrections were applied to energy and pressure. An external electric field of 1.0 V nm^{-1} was applied along the z-axis to drive ion transport through the COF pores. The Leap-frog integrator was used to integrate equations of motion, and center-of-mass motion was removed at every step. System coordinates were recorded every 1.0 ps, and energies were saved at the same frequency.

The trajectories obtained from the production MD simulations were analyzed using Python scripts to quantify the transport behavior of Li^+, Na^+, and K^+ ions through the TpEb COF. The analysis focused on identifying ions that successfully permeated the COF region, determining their permeation velocities within the pore, evaluating changes in the number of water molecules in their first hydration shell, computing radial distribution functions (RDFs), and finally, calculating the potential of mean force (PMF) profiles. Potential of mean force profiles along the membrane normal were computed from the steady-state ion number density. Ion positions were histogrammed along the z-direction over the production trajectory, yielding a normalized probability distribution P(z). An effective potential (PMF) was then defined as $PMF=-k_BT\ln[P(z)/P_{bulk}]$ where P_{bulk} is the average probability density in bulk regions. It is worth mentioning that with an external electric field applied, this quantity does not represent an "equilibrium PMF" but rather a density-derived steady-state profile referenced to bulk.

1.3.3. Computational Fluid Dynamics (CFD) simulation

Figure S6 presents the schematic of the numerical model used to simulate the electrochemical system for COF-based TFC membrane fabrication. The setup consists of a single reaction chamber, two cation-exchange membranes (CEMs), and two electrodes (anode and cathode). The CEMs are arranged in a zero-gap configuration adjacent to both electrodes.

The modeled system comprises a central reaction chamber bounded by two parallel electrodes and separated from the anodic and cathodic compartments by ion-selective membranes. The feed solution within the chamber contains both a neutral monomer species (Tp) and a negatively charged monomer ($Pa(SO_3H)_2$). An external electric potential difference (ΔV) is applied across the electrodes, generating an electric field that drives ion transport and promotes electrochemical reactions at the electrode surfaces. In this study, two operational modes were considered: (i) DC mode in which the simulations were performed under stationary (steady-state) conditions, and (ii)

AC mode in which the simulations were carried out under transient conditions to capture time-dependent behavior.

Model assumptions: To simplify the modelling process, the following assumptions are made:

1) The process is isothermal throughout operation.
2) Fluid flow within the chamber is laminar (Re = 1983.0) and incompressible.
3) The influence of pressure on the Donnan equilibrium and on ion transport is neglected.
4) Explicit two-phase transport of H_2 and O_2 gas is not resolved. As shown in Supplementary Discussion 2.7, the interfacial gas volume fraction remains below approximately 0.25%, well beneath the ≈1.0% threshold at which multiphase effects govern bulk transport, so that the single-phase treatment of ion transport is not materially affected.
5) Bubble penetration into the ion-exchange membranes is neglected. The Young-Laplace analysis in Supplementary Discussion 2.7 shows that the capillary entry pressure for the hydrated membrane channels (≥116.0 bar) exceeds the pressure available in the open chamber by two orders of magnitude, precluding bubble entry.
6) Bubble adhesion and coverage at the electrode surfaces are neglected; the associated three-dimensional coalescence and departure behavior, and its limitations, are discussed in Supplementary Discussion 2.7.
7) H^+ and OH^- production at the anode and cathode, respectively, is computed from Faraday's law.
8) The ion-exchange membranes are assumed to be ideally selective, and co-ion diffusion is neglected. Dissolved-gas crossover, evaluated in Supplementary Discussion 2.7 at approximately 1.1% of the H_2 generation rate, is charge-neutral and small relative to the ionic transport resolved by the model.

The coupled fluid flow, ionic transport, and electrochemical reaction processes were modeled using the Navier-Stokes, Nernst- Planck, and charge conservation equations, along with appropriate kinetic and thermodynamic relationships.

Fluid Flow: The incompressible Navier-Stokes equations describe the flow of the liquid phase in the reaction chamber under laminar conditions:

$$\nabla\cdot\mathbf{u}=0 \quad \text{(Equation 1)}$$

$$\rho(\mathbf{u}\cdot\nabla\mathbf{u})=-\nabla P+\mu\nabla^2\mathbf{u} \quad \text{(Equation 2)}$$

Where ρ is the solution density (kg m^{-3}), u is the velocity vector (m s^{-1}), P is the pressure (Pa), and μ is the dynamic viscosity of the solution (Pa s). Figure S7 shows the obtained velocity contour.

Ionic Transport: The Nernst-Planck equation governs the transport of ionic species, accounting for diffusion, migration, and convection:

$$N_i= - D_i\nabla c_i - Z_i u_i F c_i \nabla\phi + c_i u \quad \text{(Equation 3)}$$

Where c_i is the concentration of species i (mol m^{-3}), D_i is the diffusion coefficient (m^2 s^{-1}), Z_i is the ionic charge number, u_i is the ionic mobility (m^2 V^{-1} s^{-1}), F is Faraday's constant (96,485 C mol^{-1}), and ϕ is the electric potential (V).

The current density is determined from the sum of all ionic fluxes:

$$I_d = \sum_{i=1}^{n} F Z_i N_i \quad \text{(Equation 4)}$$

The ionic mobility is related to the diffusion coefficient via the Nernst-Einstein relation:

$$u_i=\frac{Z_i F D_i}{RT} \quad \text{(Equation 5)}$$

where R is the universal gas constant (8.314 J mol^{-1} K^{-1}) and T is the absolute temperature (K).

Within the ion-exchange membranes, effective diffusivities of molecules (e.g., ions) were corrected using the Bruggeman relation:

$$D_{i,eff}=\varepsilon^{1.5} D_i \qquad \text{(Equation 6)}$$

where ε is the membrane porosity. Consequently, the effective ionic mobility is given by:

$$u_{i,eff}=\frac{Z_i D_{i,eff}}{RT} \qquad \text{(Equation 7)}$$

Donnan Potential: At the membrane–solution interfaces, the Donnan potential (ϕ_{Don}) arises due to the unequal distribution of ions between the charged membrane and the adjacent electrolyte. It is expressed as:

$$\varphi_{Don} = \varphi_m - \varphi_s = -\frac{RT}{Z_i F}\ln\frac{C_{i,m}}{C_{i,s}} \qquad \text{(Equation 8)}$$

where ϕ_m and ϕ_s are the electric potentials in the membrane and the solution, respectively, and $C_{i,m}$ and $C_{i,s}$ denote the concentrations of ion i in the membrane and solution phases. A higher fixed charge density within the membrane corresponds to lower co-ion sorption, while increasing the external electrolyte concentration enhances co-ion penetration into the membrane phase.

Electrochemical reaction: Electrode reactions were modeled according to water oxidation and reduction processes occurring at the anode and cathode, respectively:

$H_2O \rightarrow 1/2\ O_2 + 2H^+ + 2e^-$ Anode

$H_2O + 2e^- \rightarrow 1/2\ H_2 + 2OH^-$ Cathode

The generation rate of these ions is estimated using Faraday's law of electrolysis:

$$n_{H^+} = \frac{I}{ZF} \qquad \text{Anode (Equation 9)}$$

$$n_{OH^-} = \frac{I}{ZF} \qquad \text{Cathode (Equation 10)}$$

Chemical Reactions in the Chamber: Within the reaction chamber, the neutral monomer and the negatively charged monomer undergo acid-assisted polymerization to form the COF network. The

local enrichment of protons influences the rate and direction of these reactions; leading to spatial variations near the CEM interfaces; such that regions adjacent to the CEM exhibit distinct chemical behavior. The general rate expression for the bulk reactions is expressed as:

Tp + Pa ⇒ COF (s)

The rate of COF formation follows second-order kinetics:

$$r_{COF} = k.C_{T_p}.C_{P_a} \quad \text{(Equation 11)}$$

where k = 0.3 $[\frac{mol}{m^3.s}]$ is the rate constant for COF formation.

<u>Boundary and Initial Conditions:</u> The computational domain was defined with appropriate hydrodynamic and electrochemical boundary conditions to accurately represent the physical behavior within the electrochemical system (Tables S4 and S5).

The channel walls of the reaction chamber were modeled using a no-slip boundary condition, ensuring that the fluid velocity at the wall is zero. This condition captures the correct velocity profile development and enforces physical consistency at solid-fluid interfaces. The electrodes were assigned fixed electric potentials corresponding to the applied direct current (DC) voltage specified in Table S4. For the DC case, a constant potential difference of 20.0 V was imposed across the electrodes to drive ionic migration and initiate electrochemical reactions at the electrode surfaces. For alternating current (AC) simulations, time-dependent potential waveforms (0.001 Hz) ; either square-wave or sinusoidal functions; were applied to the positive and negative electrodes, as illustrated in Table S6. The instantaneous polarity of the electrodes determines the spatial location of H^+ and OH^- generation resulting from the water-splitting reaction. During polarity reversal, the sites of H^+ and OH^- production alternate accordingly, leading to a dynamic redistribution of ionic concentrations and electrochemical activity within the chamber. Finally, the initial conditions assumed a quiescent solution with uniform ion and monomer concentrations throughout the computational domain.

The computational model was implemented in COMSOL Multiphysics® 6.3 using the finite element method (FEM). A structured map mesh (105,200 elements) with localized refinement near the membrane-solution interfaces was employed to accurately capture steep gradients in pH, ion concentration, and electric potential. Away from these interfaces, the mesh became progressively coarser to reduce computational cost while maintaining accuracy. Mesh independence tests were performed to confirm that the simulation results were insensitive to further refinement. The coupled nonlinear partial differential equations were solved using the PARDISO direct solver, with a convergence tolerance set to residual norms below 10^{-6}, ensuring both numerical stability and solution accuracy.

For alternating current (AC) simulations, a progressive initialization (continuation) strategy was adopted to improve convergence. The simulation was initially run with zero ion-exchange capacity, representing a non-charged membrane, and the ion exchange capacity was gradually increased in successive steps until the target value was reached. This gradual ramping minimizes numerical instabilities arising from abrupt changes in electrochemical source terms and establishes a physically consistent initial condition for the subsequent time-dependent AC analysis.

2. Supplementary Discussions

2.1. Effect of the voltage type on coating adhesion quality

Cross-sectional SEM images of different COF-modified membranes (Figure S14) demonstrate that DC-deposited layers are more prone to delamination and exhibit weaker adhesion to the CEM substrate, as evidenced by unstable performance and film fragmentation during testing (Figure S15). In contrast, the AC-assisted films remain mechanically stable and well-adhered during separation testing. More importantly, the use of an alternating electric field enables simultaneous COF deposition on both electrodes, effectively producing two identical TFC membranes within a

single fabrication batch. While DC operation restricts COF growth to the electrode of opposite polarity to the charged monomers, AC cycling periodically reverses the field direction, leading to symmetrical film formation on both CEMs (Figure 1b). This versatile fabrication strategy greatly improves membrane production efficiency by offering a wide range of parameters that could be tuned for a specific separation task.

2.2. Versatility of the developed fabrication method

The application of an AC field for fabricating COF-based coatings on the surface of CEMs offers broad tunability of key parameters; such as waveform shape, field intensity, and frequency; to achieve uniform and defect-free coatings tailored to specific applications.

In principle, any AC waveform can be employed within the proposed method. CFD simulations conducted under various voltage types confirmed the formation of the monomer-enriched and pH-adjusted regions adjacent to the CEM surface in all cases (Supplementary Movies 1-3). However, the surface morphology and uniformity of the resulting COF layers differed significantly depending on the waveform applied (Figure S18). Among the tested modes, i.e., sinusoidal, square, and ramp, the square waveform produced the most homogeneous and smooth COF films. This superior performance can be attributed to the more effective delivery of hydronium ions and COF-forming monomers to the interfacial reaction zone during each voltage cycle. In a square waveform, the potential instantaneously reaches the water-splitting threshold, enabling simultaneous electrophoretic monomer migration and hydronium generation. Conversely, in sinusoidal and ramp waveforms, the electric field gradually increases, allowing monomer migration to begin early in the cycle while hydronium production only occurs once the water-splitting potential is reached (Figure S13). This temporal mismatch reduces the reaction efficiency

and promotes non-uniform COF growth, emphasizing that a stable square waveform is the optimal choice for achieving uniform and continuous coatings.

The frequency of the applied AC field also plays a critical role in determining the coating quality (Figure S19). At very high frequencies (1000.0 Hz), even when water splitting occurs, the generated hydronium ions lack sufficient time to migrate through the CEM, diffuse within the membrane, and reach the monomer-enriched layer before the field polarity reverses. As a result, these ions remain trapped near the electrode-membrane interface and cannot effectively participate in catalyzing COF formation. In contrast, low-frequency operation (1.0 mHz) provides adequate time for hydronium mass transport processes to occur, thereby facilitating efficient interfacial catalysis and uniform COF layer growth.

The intensity of the electric field must be carefully optimized (Figure S20). While stronger fields accelerate the electrophoretic migration of the charged monomers, they also enhance hydrogen and oxygen gas evolution reactions via excessive water splitting. Moreover, an overly acidic microenvironment resulting from excessive hydronium generation promotes coarse COF particle formation and surface roughening, compromising the film integrity. Conversely, too weak an electric field fails to drive sufficient ionic migration or water splitting. Therefore, the optimal voltage amplitude should be slightly above the threshold for water splitting, ensuring steady hydronium ion generation without inducing gas accumulation or harsh reaction conditions. Collectively, these results highlight that square-wave AC voltages, applied at low frequencies (1.0 mHz) and moderate intensities (10.0 V), provide the ideal electrochemical conditions for controlled interfacial COF growth, enabling defect-free, well-adhered, and uniform TFC membranes.

The duration of the reaction also plays an important role due to the described self-healing and self-regulating feedback mechanism (Figure 2a). As the reaction progresses, the CEM surface becomes

heterogeneously covered, with COF nuclei forming in some regions while other areas remain exposed. This partial coverage gives rise to two distinct domains characterized by markedly different local reaction kinetics. In regions already coated with COF, the growing film functions as a resistive layer that attenuates both the local electric field and hydronium ion transport, thereby suppressing further condensation. In contrast, grain boundaries, microcracks, and uncovered regions maintain higher field strengths and hydronium ion concentrations, which sustain accelerated reaction rates. This spatial disparity creates a self-regulating feedback mechanism whereby COF growth is preferentially enhanced at defect sites until uniform coverage is achieved. This behavior is corroborated by the transition from incomplete membrane coverage after 2.0 h to a fully continuous film after 8.0 h of fabrication (Figure 2b). Consequently, the system intrinsically evolves toward homogeneous, defect-free coverage of the CEM surface without requiring external intervention or post-treatment. However, extending the reaction time beyond the optimal range (8.0 hr) leads to the deposition of coarse COF particles, which not only increases surface roughness but also promotes crack formation within the film. At prolonged reaction times (24.0 hr), bulk nucleation and particle growth become the dominant pathways, resulting in non-uniform and defective membrane structure that should be avoided (Figures S16 and S17; Table S7).

To demonstrate the versatility and general applicability of the newly developed electro-assisted fabrication method, five representative diamine monomers with distinct charge characteristics were employed: anionic ($TpPa(SO_3H)_3$,and $TpPa(SO_3H)_6$), cationic (TpEb), and neutral (TpPa, and TpHz) species (Figures S2, S21 and S22). Using these monomers, a series of defect-free COF-based TFC membranes were successfully fabricated (Figure 2h), demonstrating the versatility of the method to accommodate diverse chemical functionalities and charge states.

For the case of anionic monomers, dissolution of $Pa(SO_3H)$ and $Pa(SO_3H)_2$ monomers in water partially generates $Pa(SO_3)^-$ and $Pa(SO_3)_2^{2-}$ respectively, together with hydronium ions. To

quantify the contribution of hydronium ions in the pH of solution, the pH of systems was measured before dilution with ethanol using a calibrated glass electrode. At the quantities employed here (0.15 mmol), dissolution shifts the pH only marginally: from 7.00 to approximately 6.83 for Pa(SO_3H) and to 6.63-6.65 for Pa(SO_3H)$_2$. These excursions are an order of magnitude smaller than those achieved by electrochemical pH adjustment via water splitting (7.0 to 5.0-6.0).

Remarkably, the results revealed that even nominally neutral COF-forming monomers can undergo directed electrophoretic migration and controlled interfacial condensation under the applied electric field. This unexpected behavior is attributed to field-induced protonation and deprotonation equilibria that transiently impart charge to otherwise neutral monomers, enabling their movement toward the reaction interface. The underlying mechanism was elucidated through a combined experimental and computational investigation, confirming that electric-field-assisted charge polarization governs the migration and assembly behavior of these monomers toward the membrane-solution interface.

2.3. Mechanistic Insight into the Electrophoretic Deposition of Neutral COFs

While electrophoretic migration of anionic and cationic monomers toward oppositely charged electrodes under an applied electric field was anticipated, the method developed in this work also successfully produced homogeneous films of nonionic COFs, namely TpPa (TFC 13 in Table S3) and TpHz (TFC 14 in Table S3). This observation was unexpected, as these frameworks are formed from electrically neutral monomers in their pristine state.

A possible explanation initially considered was the protonation of amine groups in the diamine monomers within the ethanol/water solvent mixture, rendering them positively charged and thus capable of electrophoretic migration. However, this hypothesis was rejected by conducting experiments under a direct current (DC) electric field, during which TpPa and TpHz COF films

were consistently deposited on the surface of the cation-exchange membranes (CEMs) attached to the positive electrode (TFC 15-16). This behavior indicates that the migrating intermediate species must possess a net negative charge, inconsistent with the proposed protonated (cationic) amine species.

To further investigate this phenomenon, the aldehyde monomer (Tp) was identified as the key species and replaced by benzene-1,3,5-tricarboxaldehyde, which is a structural analogue lacking the hydroxyl substituents, under identical synthesis conditions used for TFC 13 and TFC 14. Remarkably, no COF film formation was observed in these control experiments. This contrast strongly implicates the critical role of the three hydroxyl groups in 2,4,6-trihydroxy-1,3,5-benzenetricarbaldehyde (Tp) in the electrophoretic process. The most likely mechanism involves partial deprotonation of the phenolic groups of Tp in the mixed ethanol/water medium, generating negatively charged Tp species capable of migrating toward the positive electrode under the applied field (Figure S23). This migration enriches the local monomer concentration near the CEM interface, thereby facilitating COF nucleation and growth. Furthermore, DFT calculations (Figure S8) revealed that the thermodynamic favorability of hydroxyl deprotonation and charge stabilization is significantly enhanced in the presence of an external electric field, supporting the proposed hypothesis and deposition mechanism of nonionic COFs.

2.4. Effect of fabrication parameters on the ion separation performance

Correlating the I-V characteristics and ED separation efficiencies of COF-based TFC membranes synthesized under varying voltage waveform, intensity, frequency, and reaction time (Figures S26-S34) confirmed that, in addition to the chemistry of the investigated COF types (Table S8), membrane integrity and defect-free nature are the dominant factors governing selectivity. Incomplete or heterogeneous COF coverage resulted in partial ion leakage and reduced selectivity,

whereas membranes fabricated under optimized AC square-voltage conditions exhibited uniform, defect-free COF layers (e.g., TpEb) that enabled precise monovalent-monovalent ion separation.

2.5. Effect of Defects on Ion Permeation Behavior Investigated by MD Simulations

To evaluate the effect of intrinsic structural confinement and defects on monovalent ion permeation behavior in the TpEb COF membrane, an additional set of MD simulations was performed using a defective TpEb COF model, and the results are presented in Figure S36. Comparison of the MD simulation results for the defect-free (Figures 3e-3h) and defective (Figure S36 b-d) membranes clearly demonstrates the critical role of defect blocking in controlling ion transport behavior.

In the case of K ions, the presence of defects had little influence on the hydration behavior during permeation. In both the defect-free and defective membranes, K ions lost approximately 2.0-3.0 water molecules while passing through the COF region. In contrast, Li and Na ions exhibited dramatically different behaviors upon the introduction of defects. For example, in the defect-free membrane, Na ions were required to lose approximately seven water molecules to enter the intrinsic COF pores. However, in the defective membrane, Na ions lost only around two water molecules on average, indicating a strong preference for transport through defective pathways rather than the intrinsic pores. A similar trend was observed for Li ions, which largely preserved their full hydration shell while traversing the defective membrane, further confirming preferential transport through the defective edge regions.

In addition, transport through these defective pathways effectively removes the dehydration-induced energy penalty and transport delay associated with entry into the confined intrinsic pores. As a result, the ions pass through the COF region with substantially lower energy barriers (around 3.0-5.0 kJ mol^{-1}; Figure 3h and Figure S36d) and significantly faster transport kinetics.

Comparison of the ion trajectories in Figure 3g and Figure S36c further highlights this effect. In the defect-free membrane, the ion transport velocities differed substantially (Li = 0.004 nm ps^{-1} Na = 0.015 nm ps^{-1}, and K = 0.016 nm ps^{-1}), reflecting the strong influence of dehydration constraints and hydration shell orientation within the intrinsic pores. In contrast, in the defective membrane, the transport velocities of all three ions became nearly identical (~0.015 nm ps^{-1}), demonstrating that the defective pathways bypass the confinement-induced selectivity mechanism and facilitate rapid ion transport through shortcut routes.

Collectively, these results highlight the critical importance of eliminating non-selective shortcut pathways, under-linked networks, and edge defects for achieving effective monovalent-monovalent ion separation in highly confined COF membrane environments.

2.6. The self-healing and self-regulating growth mechanism

The formation of a continuous, defect-free COF layer under the alternating field is governed by a self-regulating feedback loop arising from the spatial dependence of hydronium delivery at the CEM surface. Because interfacial condensation requires the coincidence of three conditions, an enriched monomer population, a sufficiently acidified local environment, and an available growth site, the local rate of film formation is set by whichever of these is limiting at a given location. In the present system it is the delivery of hydronium, generated by water splitting and transported across the support, that provides the spatial control.

Once the deposited COF begins to cover the surface, the two regions of the growing film are no longer equivalent. The deposited layer is electrically resistive relative to the hydrated ion-exchange support, and where the surface is already covered, the potential drop across the film attenuates both the local interfacial field and the flux of hydronium emerging into the reaction zone. The covered regions therefore experience a progressively less acidic local environment and a

correspondingly reduced condensation rate. Conversely, at grain boundaries, microcracks, and sites that remain uncovered, the local resistance is lower, the field and hydronium flux remain elevated, and condensation continues at a higher rate. Growth is thus preferentially directed toward the locations where the film is incomplete, so that the process is self-healing rather than merely self-limiting: defects are the sites of fastest repair, and the film evolves toward uniform coverage without external intervention or post-treatment. The self-healing mechanism proposed in this work can be analogous to a previously reported two-regime growth mechanism for COF films[23]. It is noted that the driving force for the self-healing in our work remains distinctly different, which is the ionic current and its associated hydronium flux through a non-conductive substrate; while the previous study utilized electron transfer to heal cracks on a conductive substrate. Nevertheless, the analysis of how the self-healing behavior would influence the film quality is considered similar. . This mechanism predicts a characteristic kinetic signature: rapid initial growth while a substantial fraction of the surface remains uncovered, a slower intermediate stage during which the remaining defects are infilled, and a markedly reduced growth rate once continuous coverage is established and hydronium delivery to the surface is attenuated everywhere. The evolution of coating thickness with deposition time, shown in Figure S37, exhibits precisely these three regimes.

During the first four hours (growth zone), the thickness increases rapidly, from approximately 68.0 nm at 2.0 h to 198.0 nm at 4.0 h, as COF nuclei form and spread laterally across the CEM surface. The corresponding cross-sectional images show a discontinuous, island-like morphology at this stage, consistent with partial coverage. Between approximately 4.0 and 8.0 hours (healing zone) the growth rate falls sharply, with the thickness increasing by only about 50.0 nm over the interval; over this period deposition is redirected into the cracks and gaps between the previously formed islands, progressively infilling them to produce a continuous layer. The surface morphology at 8.0 h is uniform and free of the discontinuities visible at earlier times. Beyond 8.0 hours (coarse

deposition zone) the thickness increases only marginally, reaching approximately 272.0 nm at 24.0 h. In this final regime, the attenuation of hydronium flux by the now-continuous resistive coating suppresses interfacial catalysis, and the residual growth is attributable to the slower, uncatalyzed condensation of monomers from the bulk solution depositing onto the existing film. This coarse deposition thickens the layer without improving its coverage or separation performance, consistent with the observation that membranes deposited for 24.0 h (TFC 3) show no enhancement relative to the 8.0 h optimum (TFC 2).

The convergence of the film toward uniform coverage during the healing zone, the pronounced deceleration of growth once continuity is reached, and the transition to slow bulk-limited deposition thereafter together provide kinetic evidence for the self-regulating mechanism, complementing the morphological evidence from surface and cross-sectional imaging.

2.7. Gas evolution, bubble dynamics, hydronium generation, the balance between acid supply and condensation kinetics, and the validity of the single-phase model under AC operation

Water splitting at the electrodes generates H_2, O_2, H_3O^+, and OH^- within the confined region between the electrode and the CEM, under both direct-current and alternating-field operation. Because the total quantity of each species produced is fixed by the charge passed (Faraday's law), it is identical for AC and DC operation at equal charge; the alternating waveform does not reduce the amount generated. The advantage of AC operation lies instead in the interruption of continuous single-species accumulation at a fixed location, and in the partial in-situ consumption of the products generated in successive half-cycles. This section sets out both the mechanism and the quantitative analysis establishing that the neglect of two-phase transport in the CFD model is physically justified.

A) Compensation of ionic products: Because the CEMs are selectively permeable to hydronium, a fraction of the H_3O^+ generated during a given half-cycle traverses the membrane and reaches the monomer-enriched region, where it performs the catalytic function central to the fabrication mechanism. During the subsequent half-cycle OH^- is generated at the same interface and neutralizes the residual H_3O^+, regenerating water. As both are mobile solution-phase species produced within the same confined volume, this neutralization is rapid and effectively diffusion-limited, so that the acid and base generated in successive half-cycles are substantially mutually consumed. Under DC operation, by contrast, one membrane face is held at sustained low pH and the other at sustained high pH throughout the fabrication period, with no compensating process.

B) Compensation of gaseous products: An analogous compensation applies to the gases. Oxygen is evolved at a given electrode during one half-cycle and hydrogen at the same electrode during the next. Platinum, the electrode coating used here, is a well-established catalyst for the recombination of hydrogen and oxygen to water, a property exploited in proton-exchange-membrane and alkaline water electrolysis to mitigate gas crossover. Pt-based recombination layers have been reported to convert in excess of 99.0% of the hydrogen present in an H_2/O_2 mixture back to water[24], to maintain anodic hydrogen content below 1.0 vol% under pressurized operation[25], and to reduce hydrogen crossover by 95.0% over more than 1,000 hours of continuous operation[26]. Recombination has moreover been shown to proceed on the working platinum catalyst itself rather than requiring a dedicated recombination layer, and to do so sufficiently rapidly that the associated gas consumption is difficult to resolve experimentally[27]. Partial recombination of the alternately evolved H_2 and O_2 at the Pt/Ti surfaces is therefore a plausible contributing process under our alternating waveform, although we note that recombination kinetics under strong polarization differ from those in the unpolarized

recombination layers of the cited studies, and we do not attribute the observed behavior to this process alone.

C) Interrupted accumulation: The principal effect, independent of recombination, is the interruption of continuous single-species evolution. Under DC operation a single gas species is evolved continuously at the same interface throughout fabrication, and in the zero-gap configuration employed here this uninterrupted accumulation produces trapped bubbles with insufficient volume to dissipate, exerting mechanical stress on the forming coating and promoting crack initiation (Figure S15b). Under AC operation, generation at any given interface ceases every half-cycle, allowing previously formed bubbles to detach and disperse before further gas is evolved at that location. The peak local gas hold-up at either interface is thereby reduced even though the total gas generated is unchanged, and the balance between accumulation and dispersal can be tuned through the field intensity and frequency.

D) Gas volume fraction in the interfacial region: The cell draws a current density of approximately 1 mA cm^{-2}, corresponding to a total current of 5.63×10^{-2} A. From Faraday's law, assuming unit Faradaic efficiency, the molar generation rates are $\dot{n}(H_2) = 2.92 \times 10^{-7}$ mol s^{-1}, and $\dot{n}(O_2) = 1.46 \times 10^{-7}$ mol s^{-1}, a combined volumetric rate of approximately 10.7 µL s^{-1} at 298.0 K and 1.0 atm. Equilibrium solubilities from the Young-Battino Henry's-law correlation give $C_{sat(H_2)} = 0.782$ mol m^{-3} and $C_{sat(O_2)} = 1.288$ mol m^{-3}. Applying film theory across the electrode-membrane gap ($\delta = 5$ µm), with $D_{H2} = 6.28 \times 10^{-9}$ m^2 s^{-1} and $D_{O_2} = 2.53 \times 10^{-9}$ m^2 s^{-1}, yields local dissolved concentrations of $C_{local(H_2)} \approx 0.823$ mol m^{-3} and $C_{local(O_2)} \approx 1.339$ mol m^{-3}, i.e. 1.05 and 1.04 times saturation, corresponding to equivalent gas volume fractions of $\varphi_{H_2} \approx 0.10\%$ and $\varphi_{O_2} \approx 0.13\%$. Because the molar flux is independent of δ while the mass-transfer coefficient scales inversely with it, the excess concentration scales linearly with the assumed film thickness; at $\delta = 10.0$ µm the fractions rise to $\varphi_{H_2} \approx 0.20\%$ and $\varphi_{O_2} \approx 0.25\%$. In both cases the combined gas fraction remains

below the 1.0% threshold, below which the multiphase effects are conventionally taken to govern bulk transport[28,29], and the interfacial gap occupies only 0.028% of the total chamber volume. Two-phase Eulerian formulations are standard for water electrolysis[29], where gas fractions exceed 10.0% and produce substantial electrode coverage and flow recirculation. Therefore, since the present system does not approach that regime, the approximation of bulk transport based on the single-phase treatment is justified.

E) Bubbles cannot mechanically enter the CEM: Whether nucleated bubbles can penetrate the membrane bears directly on the defect-formation mechanism. The CMX-fg support is a dense, gel-type homogeneous ion-exchange membrane in which transport occurs through hydrated ion pathways of approximately 0.5-2.0 nm, whereas electrolytic bubbles nucleate from sub-micrometer nuclei and grow to several micrometers before detachment. The Young-Laplace capillary entry pressure ($PC = 2\gamma\cos\theta/r$), evaluated with $\gamma \approx 23.0\text{-}25.0$ mN m^{-1} for the ethanol/water medium[30], and $\theta \approx 61.0^{\circ}$ for a hydrated CMX-type surface[31], gives entry pressures of about ≈ 465.0 bar at r = 0.5 nm, ≈233.0 bar at 1.0 nm, and ≈116.0 bar at 2.0 nm. Even at the upper bound of the hydrated ion pathways, the required entry pressure exceeds the pressure (≈1.0 bar) in the open chamber by two orders of magnitude. Bubble penetration into the membrane is therefore not possible, and its omission from the CFD model is a physically justified simplification.

F) Dissolved-gas crossover: The dissolved H_2 and O_2 can, unlike bubbles, diffuse through the hydrated ion pathways that conduct hydronium, which is the established transport mechanism in the membranes of PEM and AEM electrolysis. Applying Fick's law with a Bruggeman correction ($D_{eff} = \varepsilon^{1.5} D$) for $\varepsilon = 0.07$ and a thickness of 160.0 μm gives $D_{eff(H_2)} \approx 1.16 \times 10^{-10}$ m^2 s^{-1} and $D_{eff(O_2)} \approx 4.69 \times 10^{-11}$ m^2 s^{-1}, and crossover fluxes of 3.2×10^{-9} and 2.1×10^{-9} mol s^{-1}, the former corresponding to approximately 1.1% of the H_2 generation rate. This transport is physically real, but as a charge-neutral species it carries no current and contributes negligible mass flux relative to

the ionic transport resolved by the model, and therefore does not measurably affect the predicted pH or ion-concentration fields.

G) Sufficiency of the supersaturation for nucleation: Expressed as a dimensionless supersaturation ratio $\zeta = (C_{local} - C_{sat})/C_{sat}$, the film-theory estimate gives $\zeta \approx$ 0.04-0.05 at δ = 5.0 µm, rising to $\zeta \approx$ 0.08-0.11 at δ = 10.0 µm. Experimental studies of gas-evolving electrodes report heterogeneous nucleation only once ζ reaches approximately 5.0-10.0[32], which is roughly two orders of magnitude higher than that in this current work. This also clarifies the visible gas evolution observed under DC operation (Figure S15b). The film-theory value is a spatial average across the gap, whereas nucleation is governed by the local concentration immediately at the electrode surface, which is higher than the gap-averaged value and is further amplified at real surfaces by roughness, pits, and microcavities that lower the nucleation barrier at discrete sites. Thus the estimation in this current work is considered as a lower bound of the local supersaturation, and the discrepancy indicates that nucleation is localized at surface features rather than distributed uniformly.

(H) Faradaic efficiency and hydronium generation rate: Under the optimized conditions the cell draws a current density of approximately 1.0 mA cm^{-2}, corresponding to a total current of 5.63×10^{-2} A. Applying Faraday's law to the anodic half-reaction ($2H_2O \rightarrow O_2 + 4H^+ + 4e^-$) the hydronium generation rate is $\dot{n}(H_3O^+) = 5.83 \times 10^{-7}$ mol s^{-1}, with the corresponding gas-generation rates $\dot{n}(H_2)$ and $\dot{n}(O_2)$; set out above (subsection D). These rates are fixed by the charge passed and are therefore identical under AC and DC operation at equal charge.

(I) Balance between acid supply and condensation kinetics: Over the 8.0-hour deposition, the total hydronium generated is about 1.7×10^{-2} mol. This is to be compared with the monomer introduced into the chamber, 1.0-1.5 $\times 10^{-4}$ mol (0.1 mmol aldehyde and 0.15 mmol diamine). Hydronium is therefore supplied in approximately hundred-fold stoichiometric excess relative to the

condensation reaction. Thus, the rate of film formation is consequently not limited by the quantity of acid generated.

J) Basis for the single-phase treatment: The CFD model used in this work, resolves single-phase ion transport, interfacial pH evolution, and the Donnan equilibrium at the interface of the membrane, rather than macroscopic bubble motion, electrode coverage, or gas crossover. The analyses above establish that the quantities the model does resolve are not materially perturbed by the phenomena it omits: the bulk gas fraction lies below the multiphase threshold, bubbles cannot enter the membrane, and dissolved-gas crossover is charge-neutral and negligible. The bubble effect invoked in the manuscript is accordingly a mechanical phenomenon evidenced experimentally (Figure S15), rather than one that the model is intended to reproduce.

3. Supplementary Figures

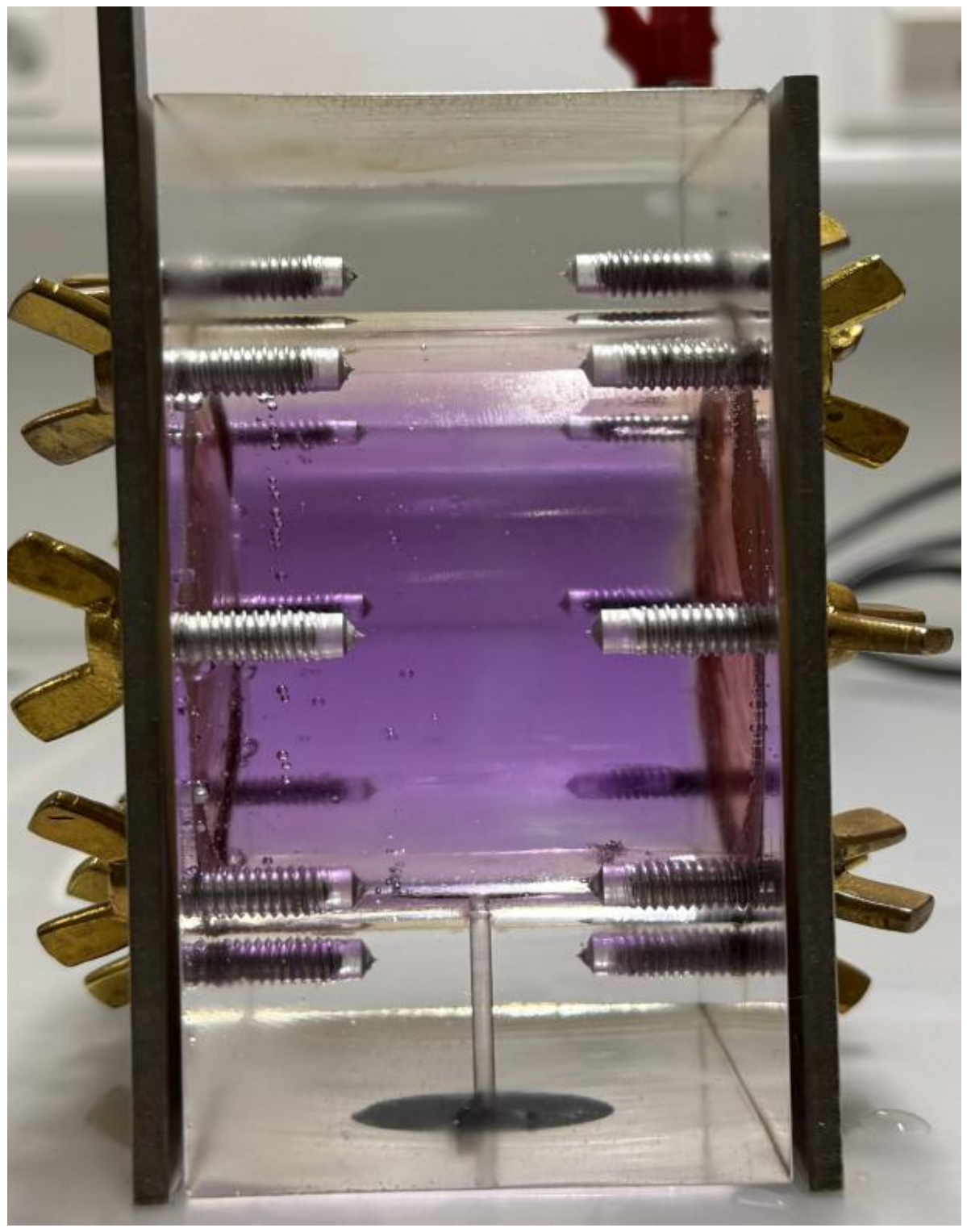

Figure S1. Custom-built transparent fabrication chamber.

Side-view photograph of the custom-built transparent fabrication chamber made of PMMA, used for real-time optical monitoring of the membrane fabrication process.

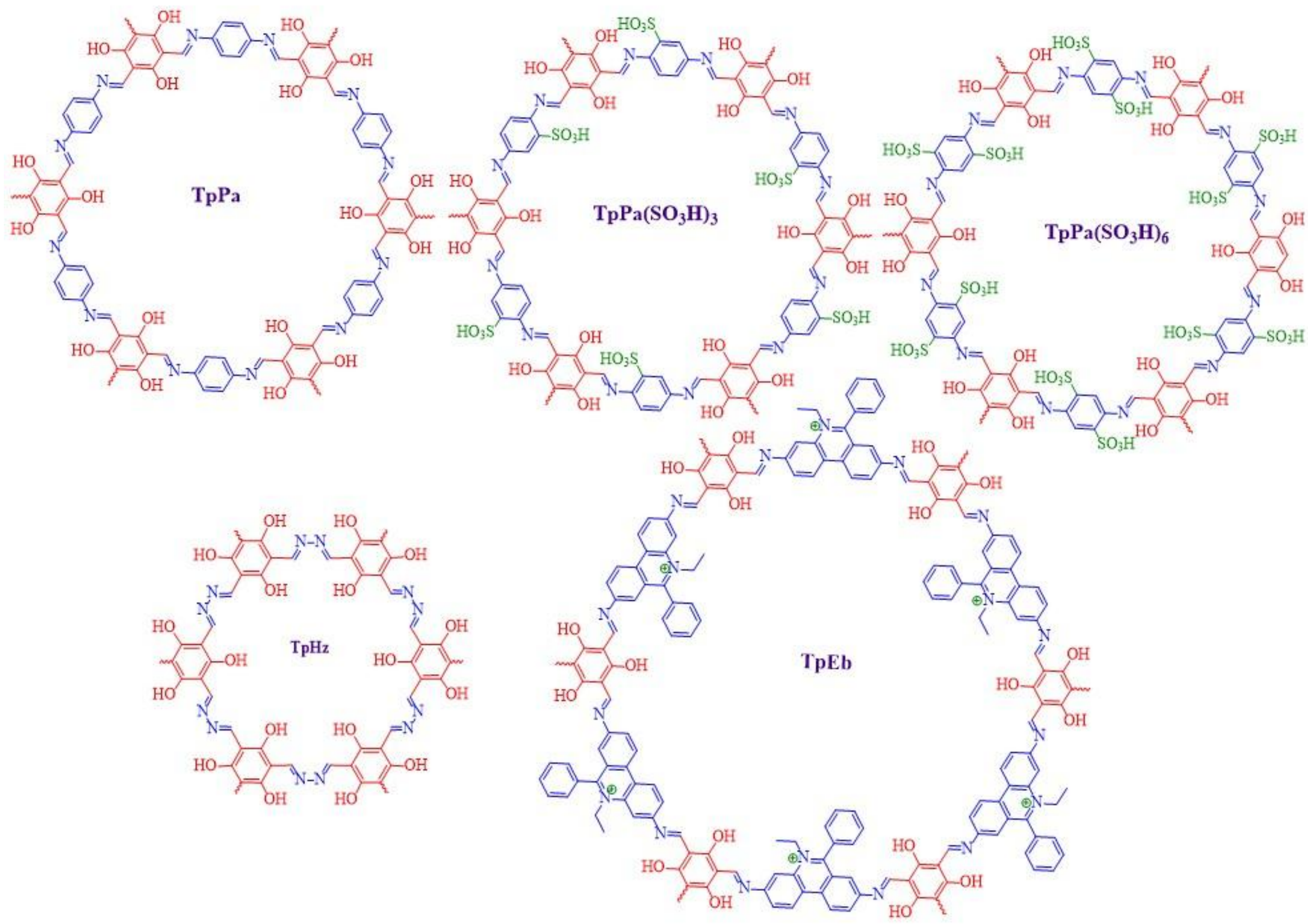

Figure S2. Synthesized COFs.

Chemical structures of the covalent organic framework (COF) materials synthesized in this work.

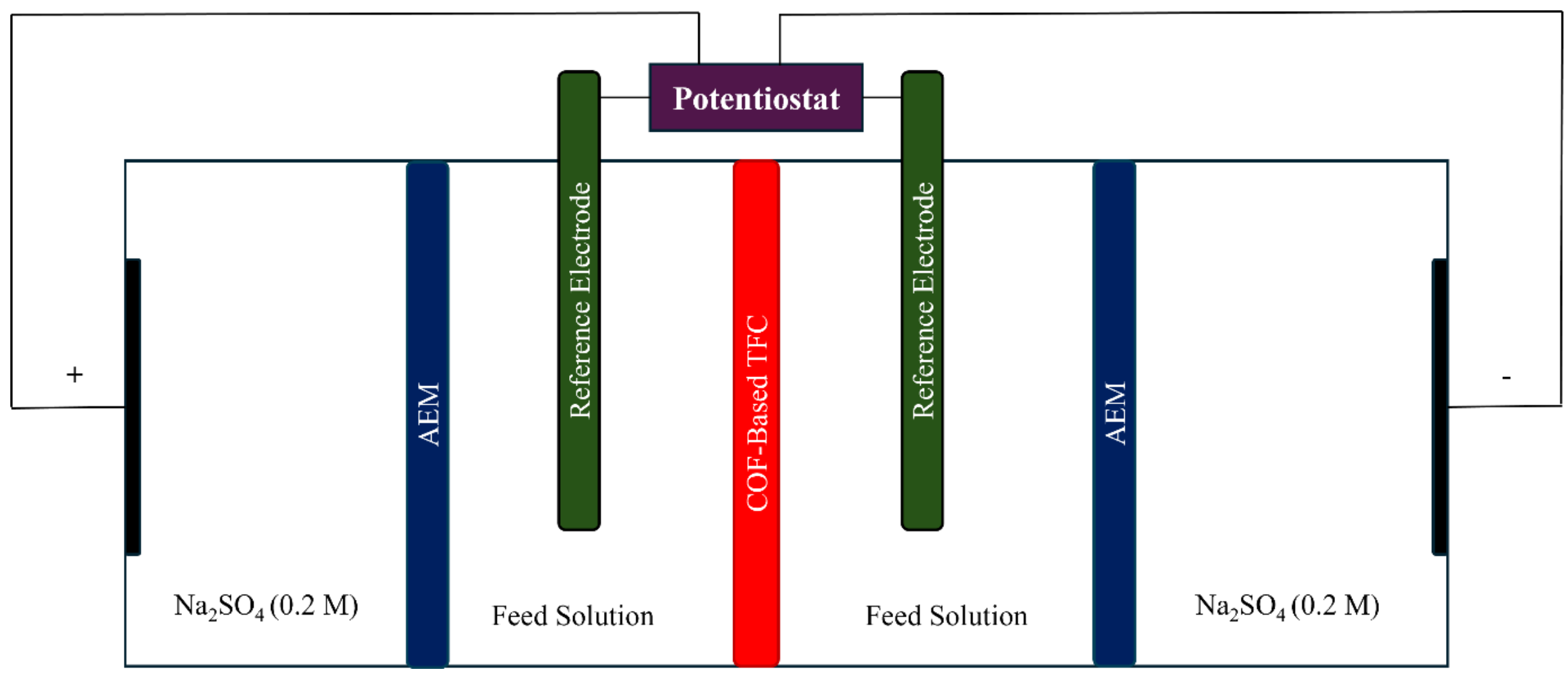

Figure S3. Electrochemical cell.

Schematic illustration of the lab-fabricated four-compartment electrochemical cell used for EIS and I-V measurements.

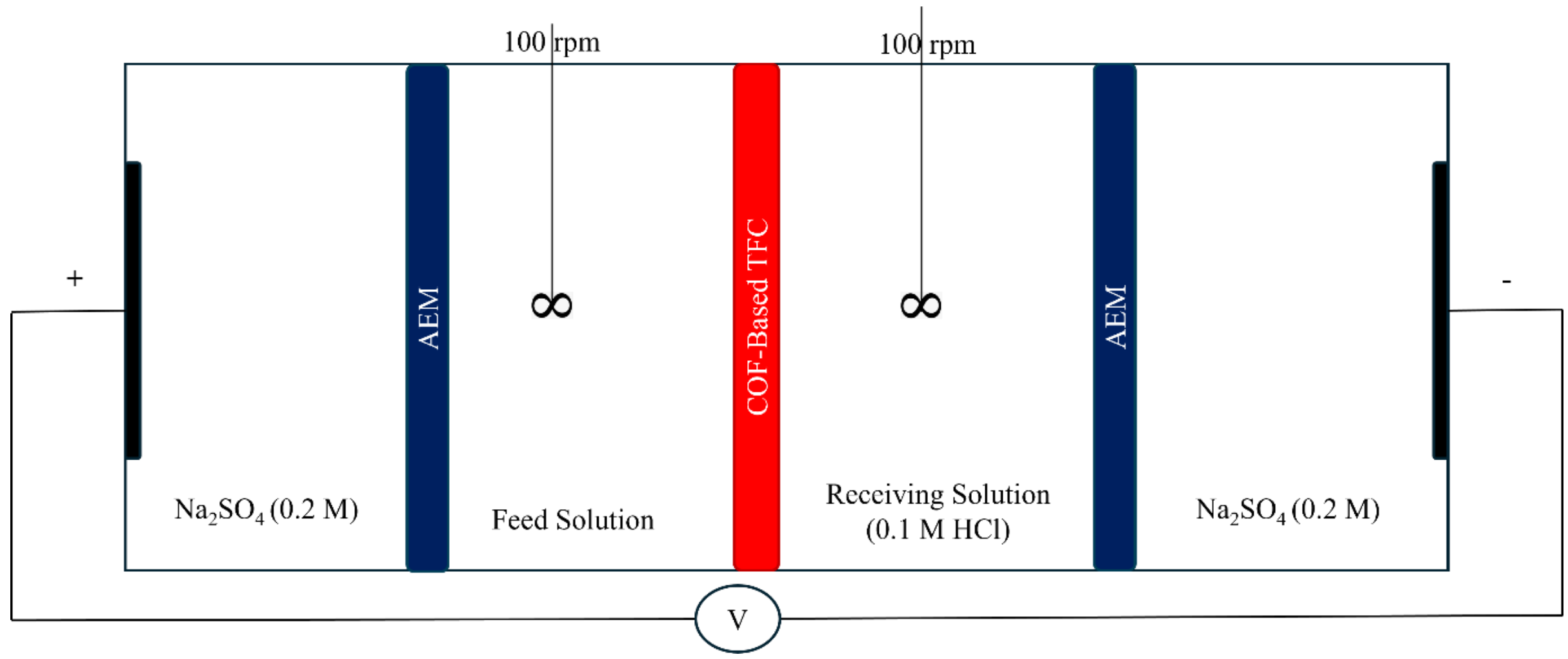

Figure S4. Electrodialysis cell.

Schematic illustration of the lab-fabricated four-compartment electrochemical cell used for ED tests.

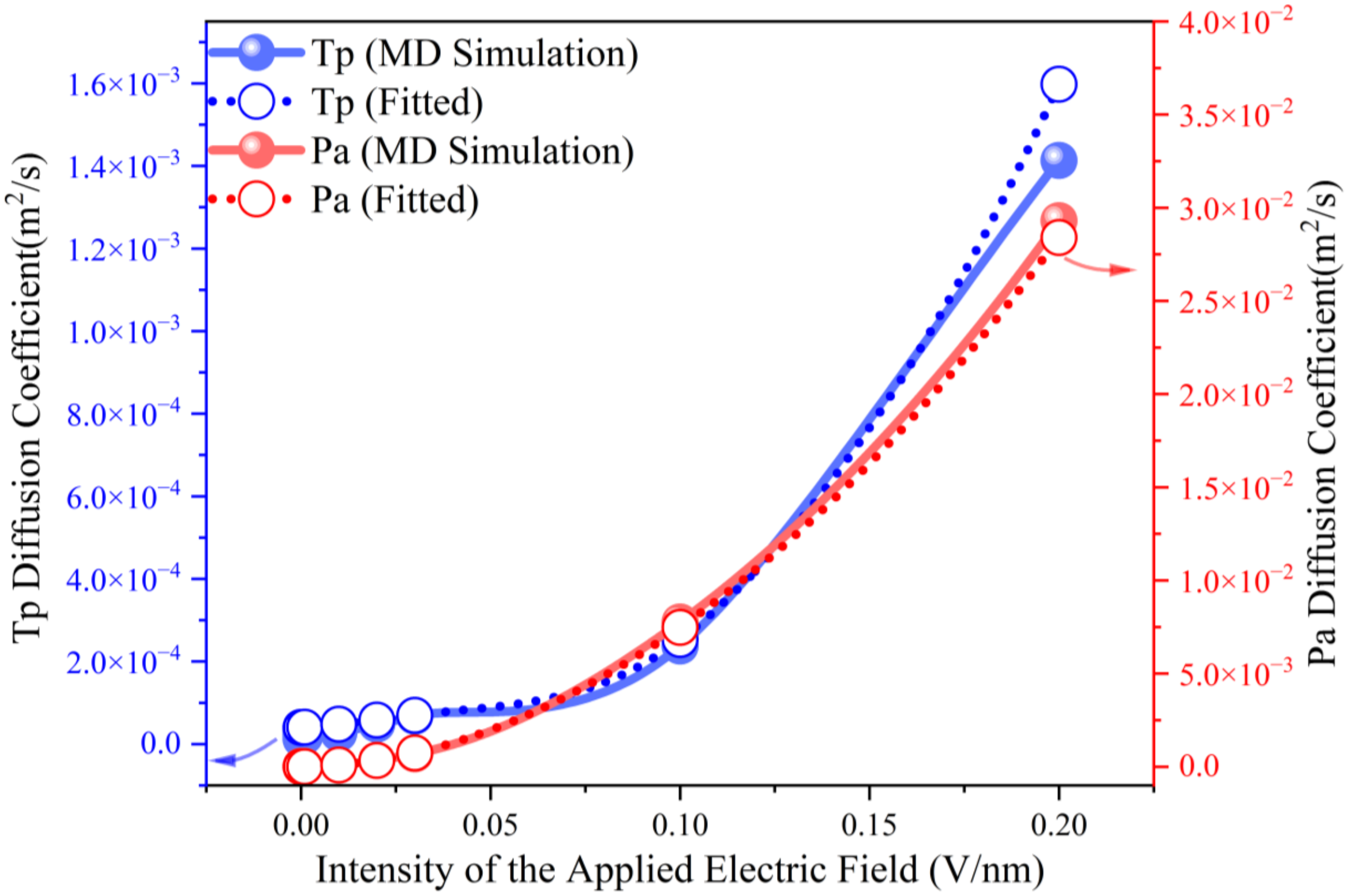

Figure S5. Simulated diffusion coefficients.

Dependence of diffusion coefficients on electric field intensity for Pa and Tp monomers, as obtained from MD simulations.

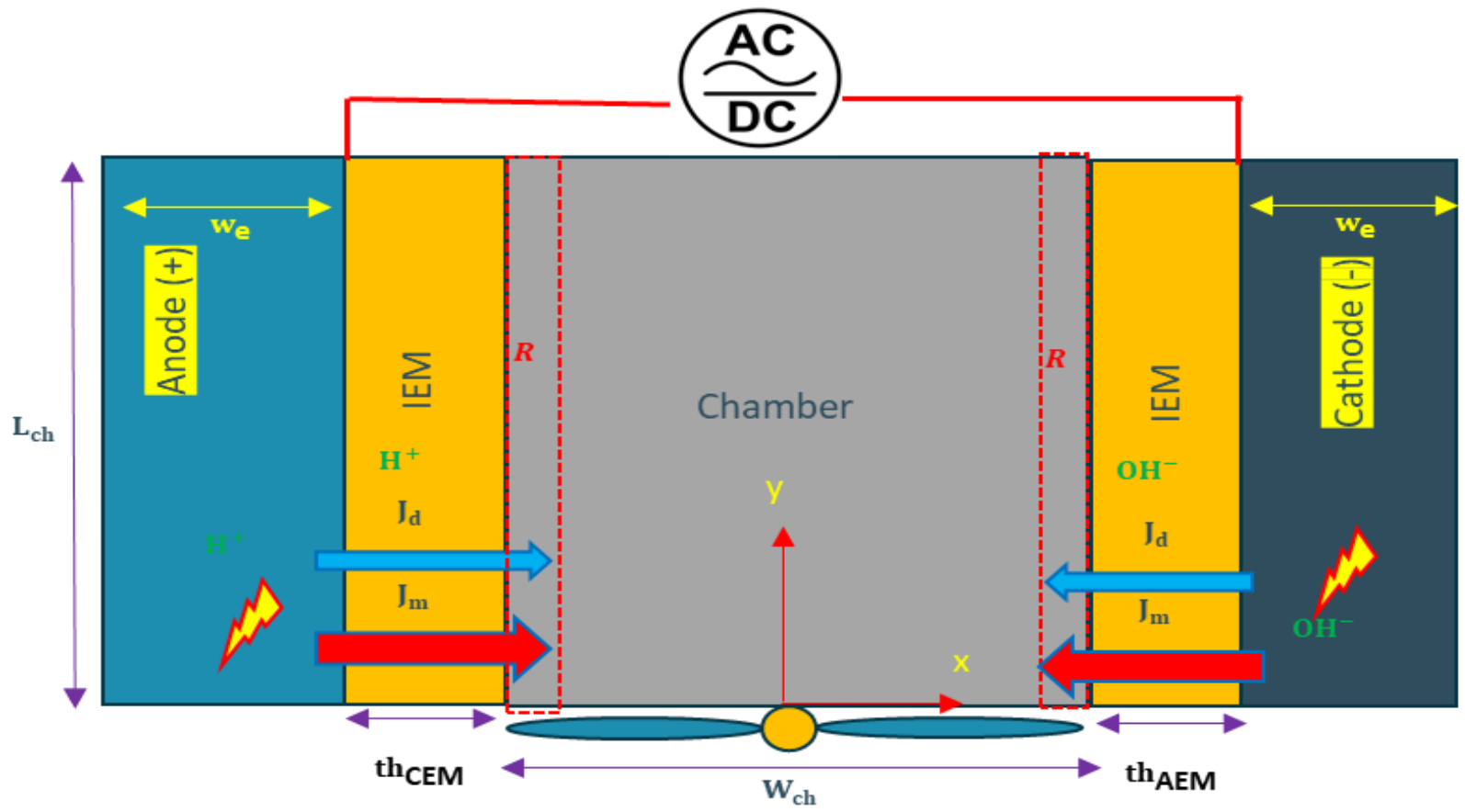

Figure S6. Definition of parameters used in CFD simulations.

Schematic view of electrochemical setup used for CFD simulations. The computational domain consists of a chamber (W_ch) separated by a CEM. A rotating flow (ω = 1500.0 rpm, Re = 1983.0) drives convective transport in the chamber. Mass transport incorporates ion diffusion coefficients. All simulations were performed at ambient temperature (T = 298.15 K) (See table S5 for the definition of the parameters).

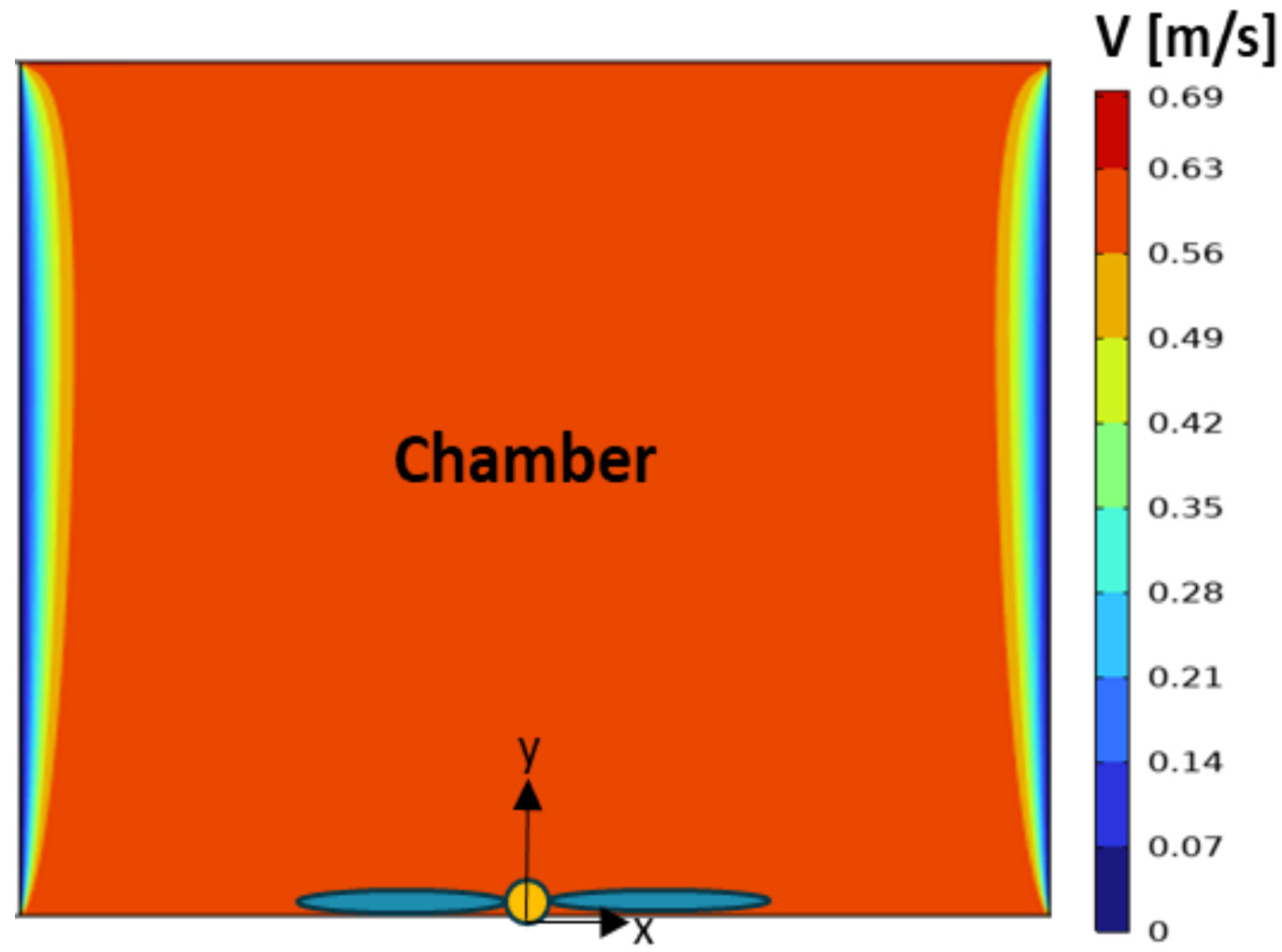

Figure S7. Velocity distribution in the fabrication chamber.

Simulated velocity-magnitude contour in the fabrication chamber. The contour exhibits boundary-layer behavior, where the balance between viscous and inertial forces results in reduced flow velocities near the CEM-chamber interface.

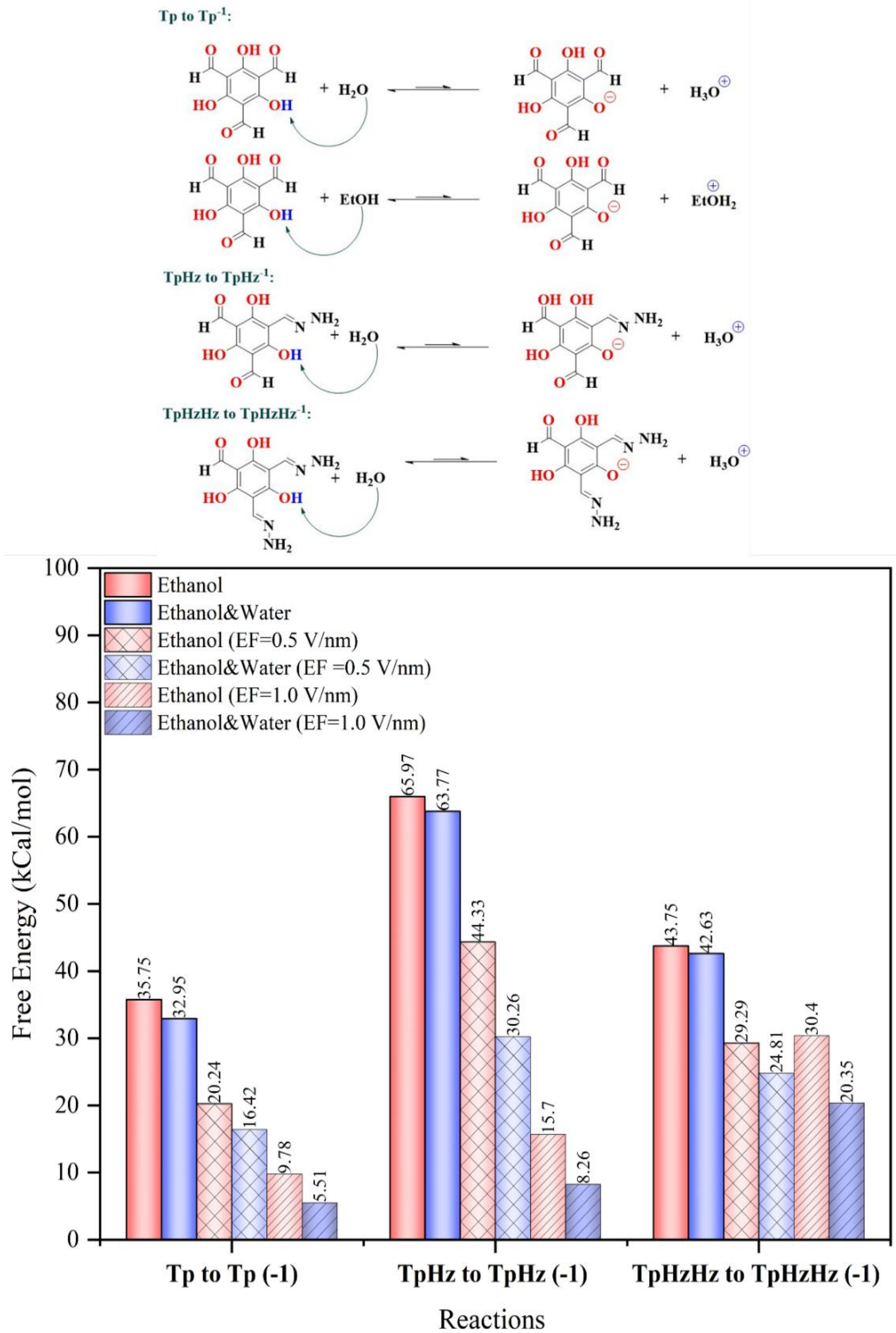

Figure S8. Effect of electric field on the deprotonation of monomers.

Top: Schematic representation of the partial deprotonation reactions of the phenolic -OH groups in the Tp monomer and the formation of their corresponding intermediates in ethanol and water media. Bottom: Free-energy profiles of the deprotonation reactions obtained from DFT calculations, illustrating the enhanced thermodynamic favorability at higher electric field intensities and upon addition of water as a co-solvent.

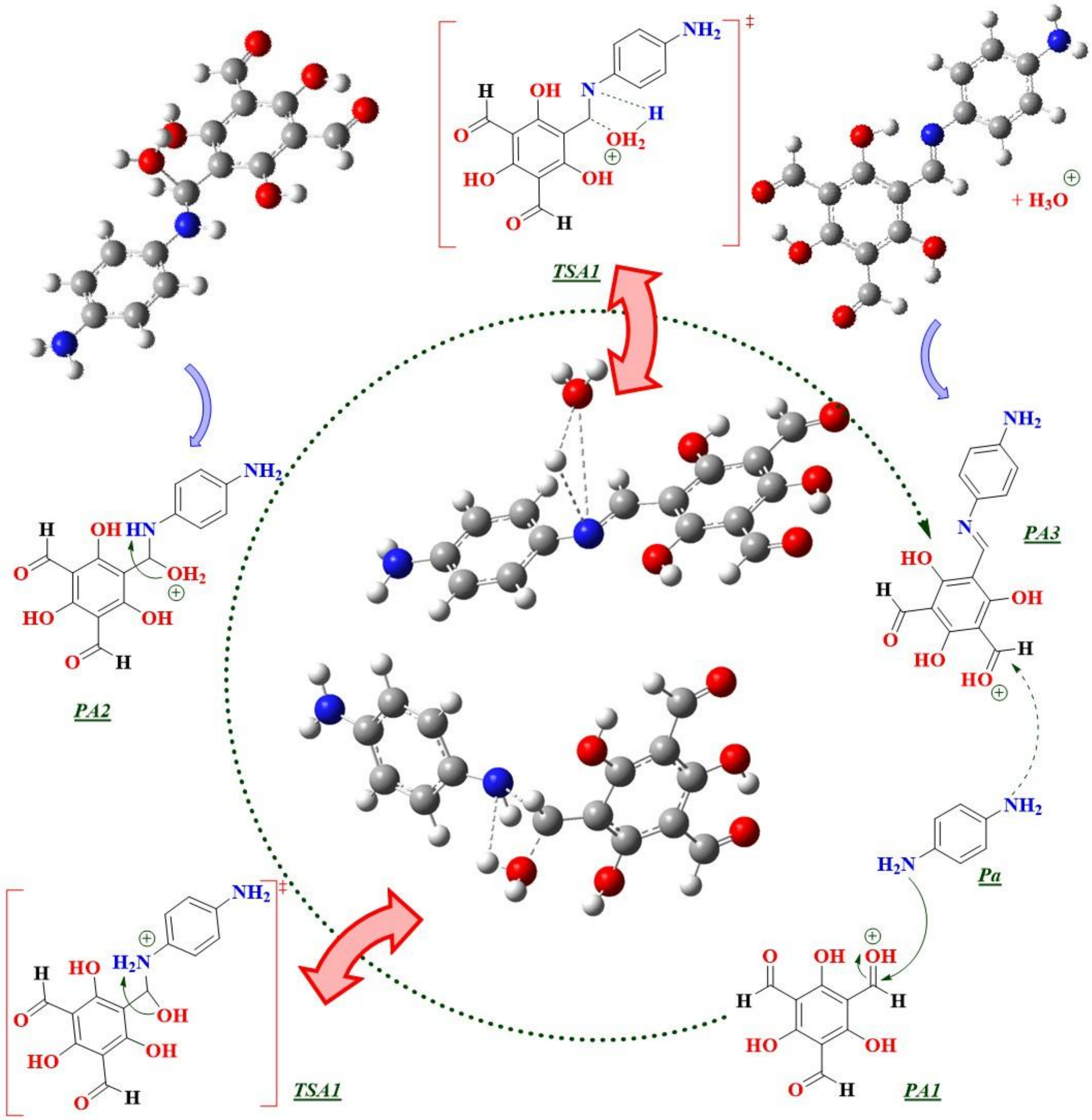

Figure S9. Mechanism of imine-link formation catalyzed with protons.

Proposed reaction mechanism between Tp (2,4,6-trihydroxy-1,3,5-benzenetricarbaldehyde) and Pa (p-phenylenediamine) leading to the formation of the imine-linked TpPa COF in the presence of an acid catalyst. The scheme illustrates the stepwise condensation between the aldehyde and amine functional groups, resulting in the formation of C=N linkages and extended conjugated COF structures.

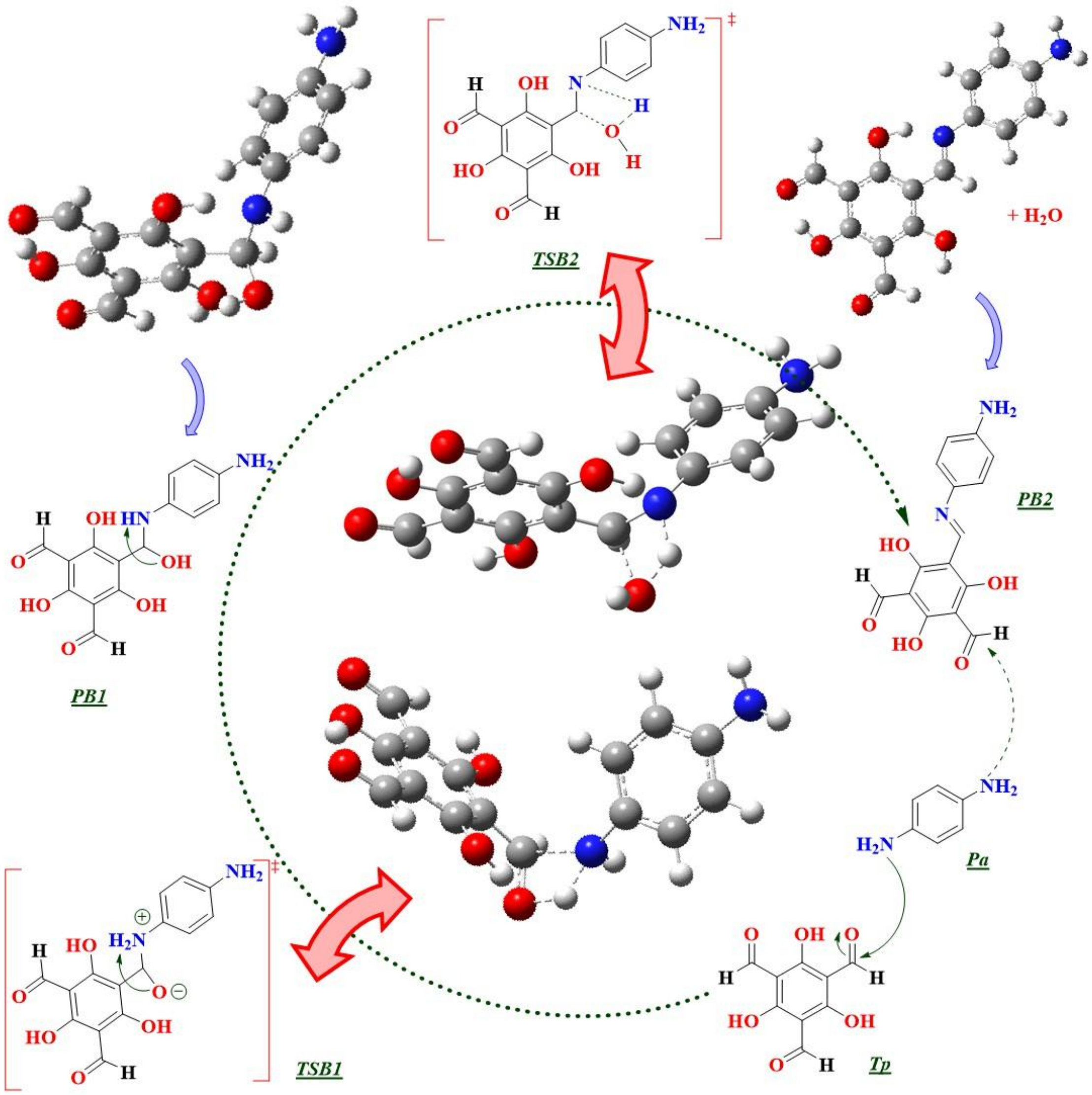

Figure S10. Mechanism of imine-link formation without protons.

Proposed reaction mechanism between Tp (2,4,6-trihydroxy-1,3,5-benzenetricarbaldehyde) and Pa (p-phenylenediamine) leading to the formation of the imine-linked TpPa COF in the absence of an acid catalyst. The scheme illustrates the stepwise condensation between the aldehyde and amine functional groups, resulting in the formation of C=N linkages and extended conjugated COF structures.

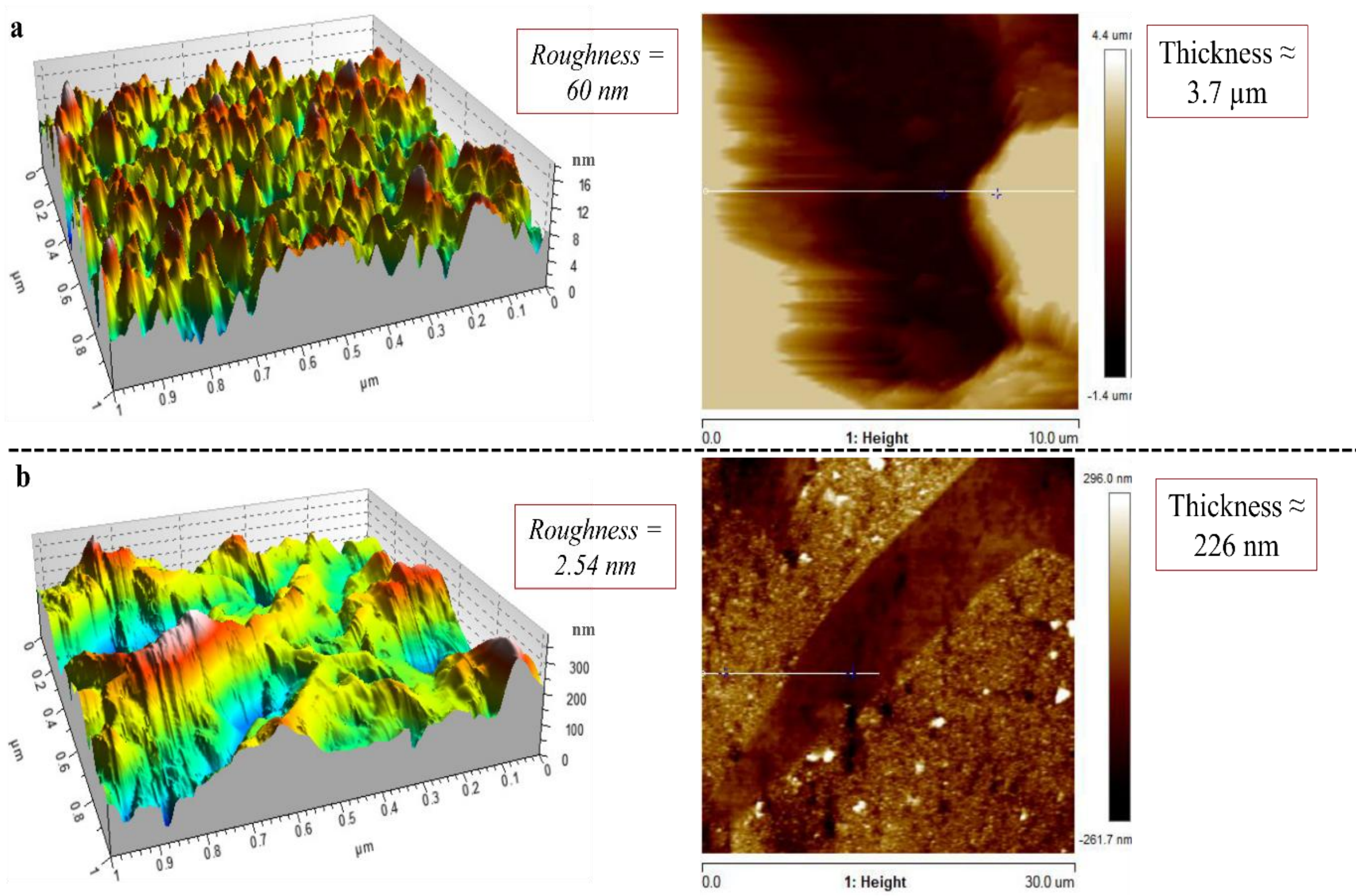

Figure S11. Effect of the type of electric field on the surface morphology of TFC membranes. Evaluation of the surface roughness and coating thickness of TpEb-coated TFC membranes fabricated under DC (a, TFC 15) and AC (b, TFC 2) electric-field-assisted deposition. Under otherwise identical deposition conditions, the AC-deposited COF film exhibits an approximately 15-fold reduction in thickness and an approximately ~23-fold decrease in surface roughness compared with the DC-deposited film.

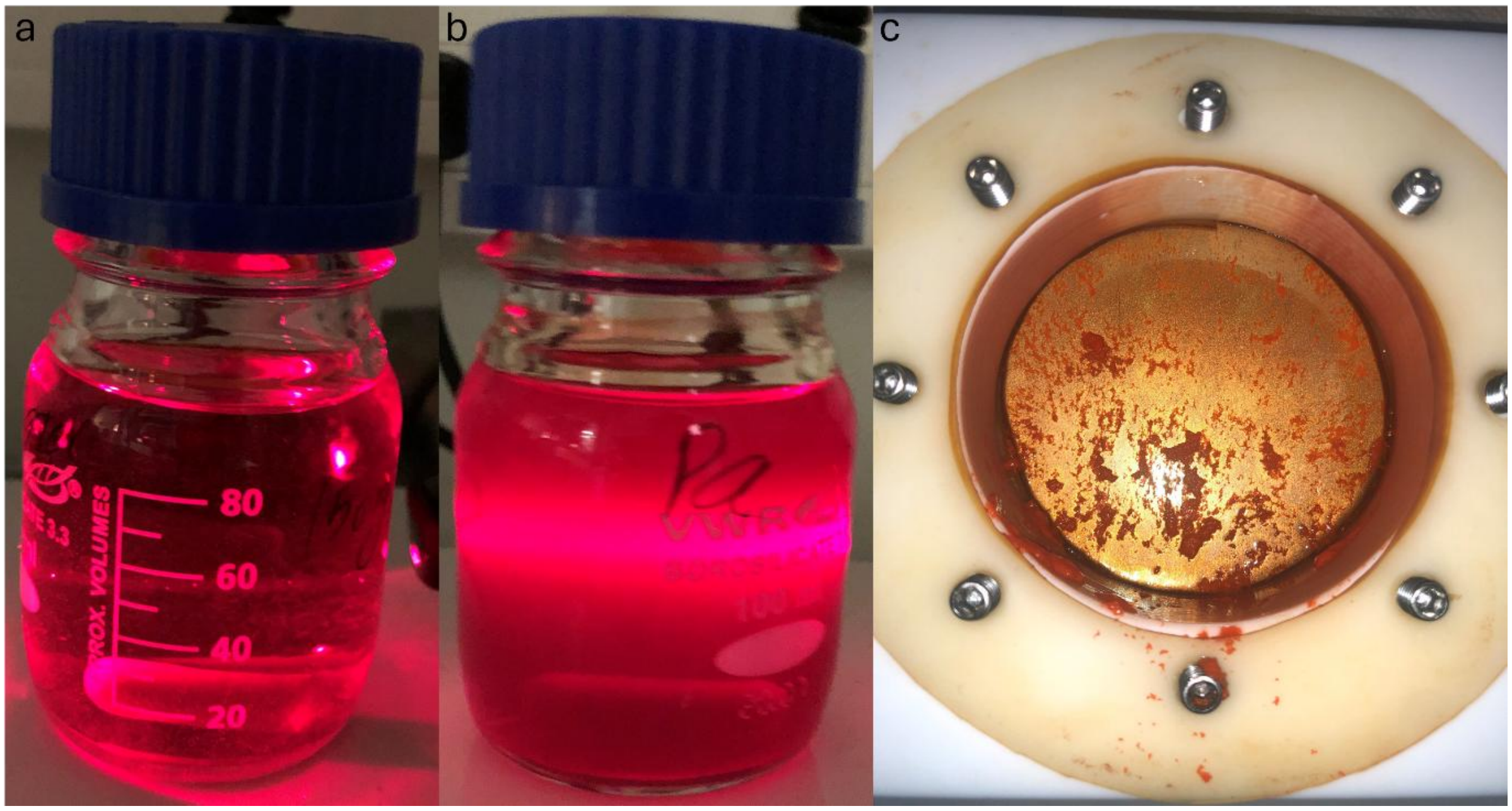

Figure S12. Effect of the addition of external organic acids.

(a) Tyndall effect captured 15.0 min after adding the $Pa(SO_3H)$ monomer to the Tp monomer for $TpPa(SO_3H)_3$ COF formation. The absence of a visible light path indicates that coarse, heavy particles have not yet formed. (b) Tyndall effect captured 15.0 min after adding the $Pa(SO_3H)_1$ monomer to the Tp monomer together with 300.0 μL of external octanoic acid. The clearly visible light path confirms the formation of coarse and heavy particles, which lack sufficient electrophoretic mobility to migrate toward the membrane surface and form a defect-free coating. (c) Photograph of the opened fabrication chamber after the addition of 300.0 μL of octanoic acid. The presence of the external weak organic acid promotes COF particle formation in the bulk solution rather than at the membrane interface. This observation highlights the critical role of localized pH regulation near the CEM surface; arising from water-splitting reactions; in directing COF growth and enabling selective film formation at the membrane interface.

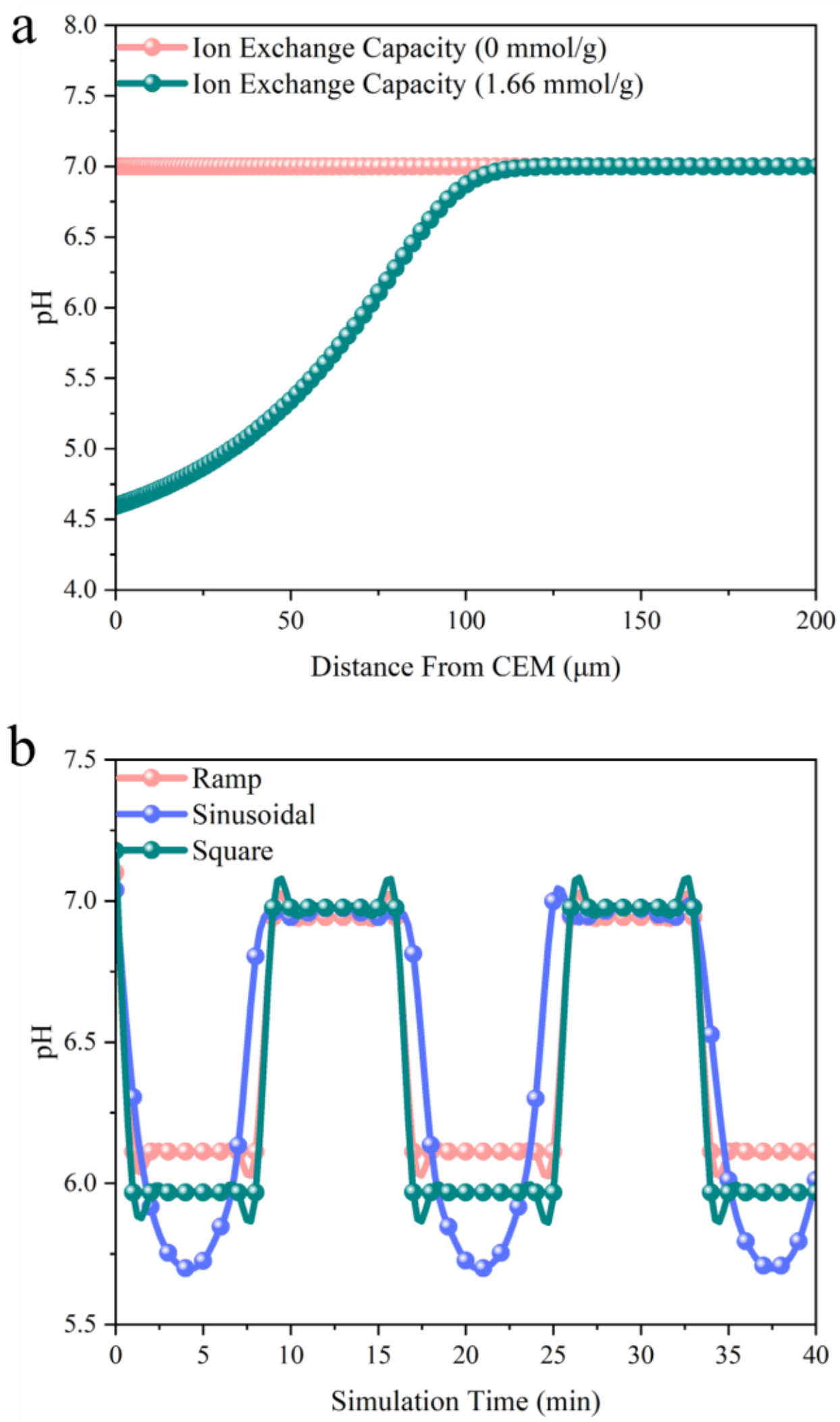

Figure S13. Calculated pH profiles near the CEM in the steady-state and transient CFD simulations.

a) Steady-state pH profile as a function of distance normal to the CEM-chamber interface, where the horizontal axis represents the distance from the membrane surface (0.0 μm) extending up to 200.0 μm into the chamber. b) Transient pH evolution as a function of simulation time (0.001 Hz) for three different applied voltage waveforms, where pH values are extracted as the average along a line located 1.0 μm from the CEM-chamber interface (Supplementary Movies 1-3). The simulations reveal the formation of a localized interfacial boundary layer approximately 100 μm thick, within which the pH remains significantly lower than that of the bulk solution due to the accumulation of hydronium ions generated by water-splitting reactions. Moreover, the applied voltage waveform exerts a deterministic influence on the local pH within this hydronium-enriched interfacial region, highlighting the critical role of waveform shape in regulating interfacial acidity.

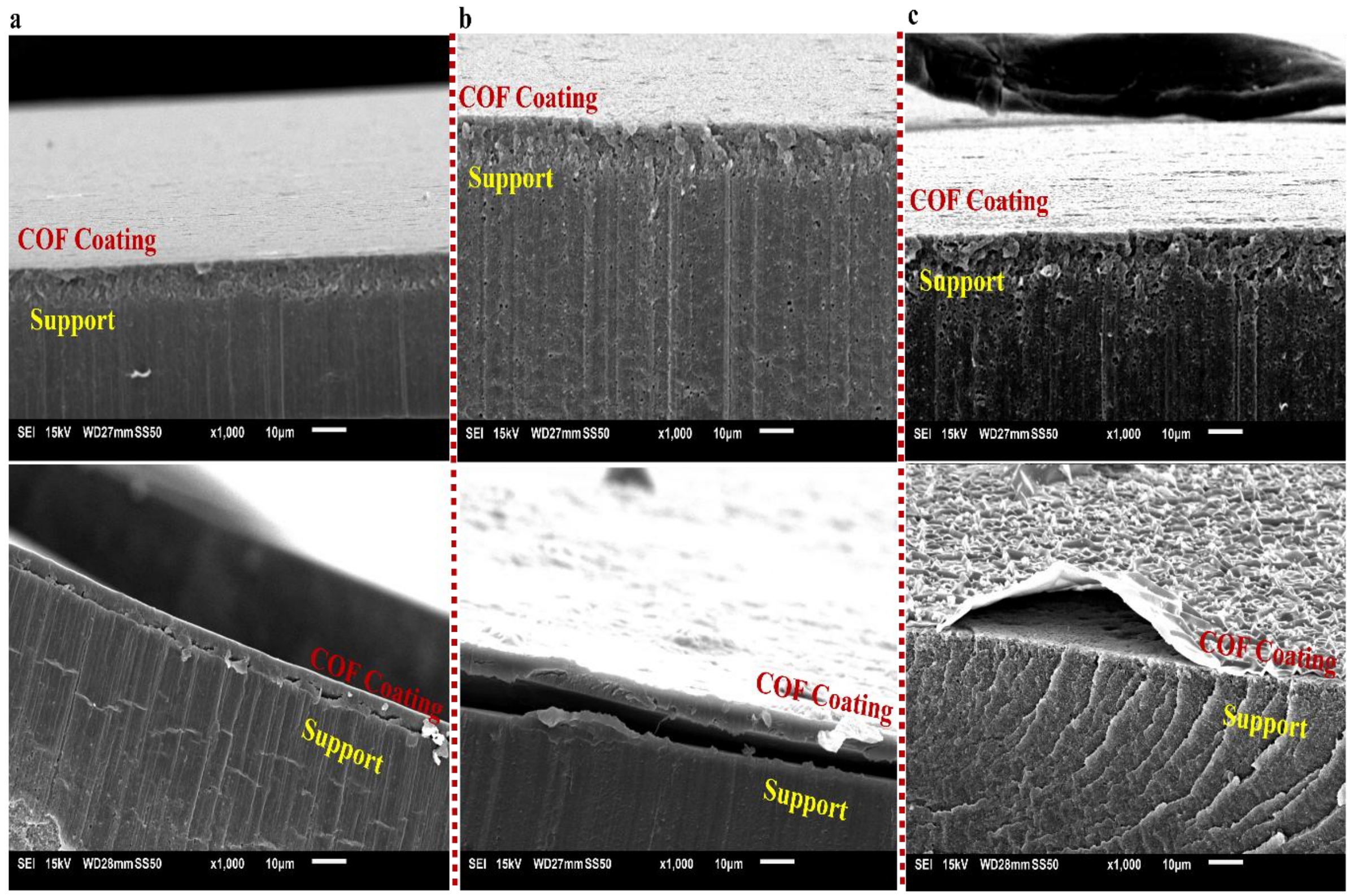

Figure S14. Effect of the type of electric field on the adhesion quality of COF films.
Cross-sectional SEM images comparing the adhesion quality and film integrity of COF-based TFC membranes synthesized under AC (top panels) and DC (bottom panels) electric fields. The membranes fabricated using AC voltage exhibit strong adhesion and integration of the COF layer with the CEM substrate, whereas delamination and poor interfacial bonding are evident in the membranes prepared under DC voltage. (a, TpEb) TFCs 2 and 15 , (b, TpPa) TFCs 13 and 16, (c, TpHz) TFCs 14 and 17.

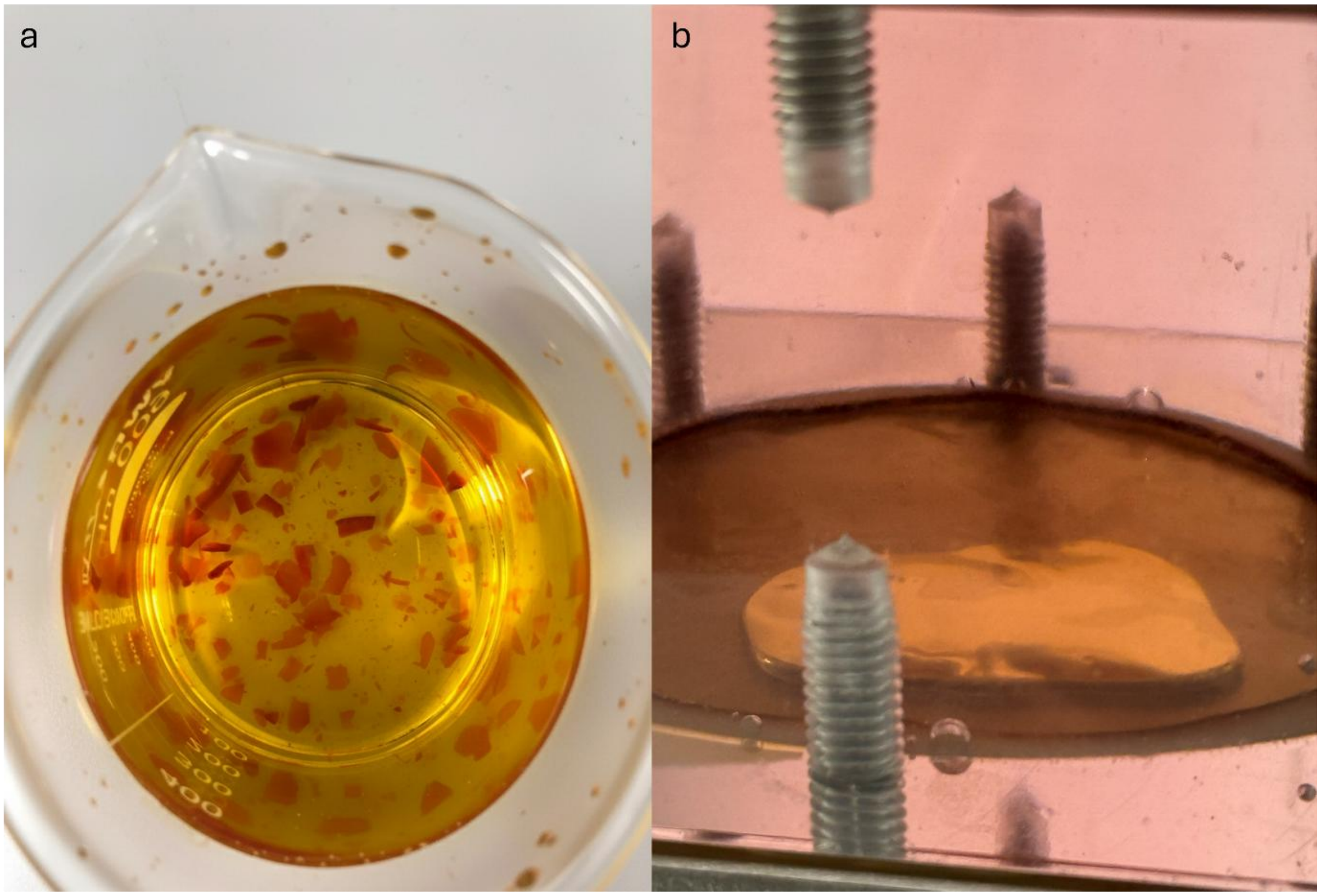

Figure S15. Effect of the type of electric field on the adhesion quality of COF films and bubble formation.

(a) Photograph showing the detachment of the TpHz COF layer synthesized under DC voltage after the washing step. The loss of film integrity demonstrates the weak adhesion and poor mechanical stability of COF layers deposited under a direct electric field, in contrast to the stable coatings obtained using alternating electric fields. (b) Photograph showing the formation and accumulation of hydrogen/oxygen bubbles between the electrode and the CEM support during DC-based deposition. The trapped gas bubbles not only impose mechanical stress on the membrane; thereby increasing the likelihood of crack initiation and propagation; but also disrupt the transport pathway of hydronium ions to the monomer-enriched region, further compromising film formation.

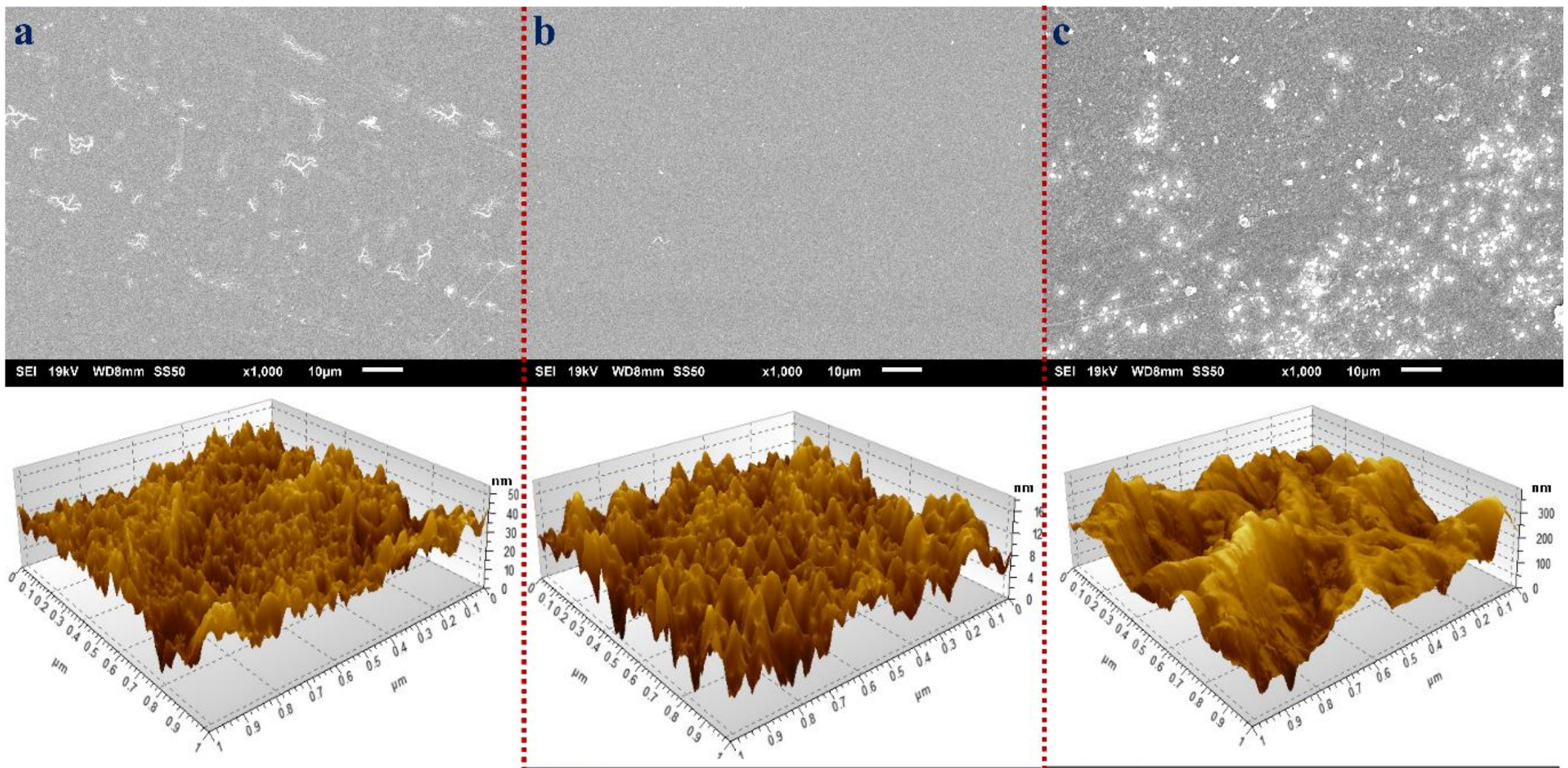

(a)

ISO 25178			
Height Parameters			
Sq	6.54	nm	Root mean square height
Ssk	-0.0155		Skewness
Sku	3.33		Kurtosis
Sp	24.4	nm	Maximum peak height
Sv	30.3	nm	Maximum pit height
Sz	54.7	nm	Maximum height
Sa	5.15	nm	Arithmetic mean height

(b)

ISO 25178			
Height Parameters			
Sq	2.54	nm	Root mean square height
Ssk	-0.0203		Skewness
Sku	2.81		Kurtosis
Sp	8.70	nm	Maximum peak height
Sv	9.75	nm	Maximum pit height
Sz	18.5	nm	Maximum height
Sa	2.04	nm	Arithmetic mean height

(c)

ISO 25178			
Height Parameters			
Sq	60.6	nm	Root mean square height
Ssk	0.103		Skewness
Sku	3.18		Kurtosis
Sp	202	nm	Maximum peak height
Sv	193	nm	Maximum pit height
Sz	395	nm	Maximum height
Sa	47.3	nm	Arithmetic mean height

Figure S16. Effect of the duration of deposition on the surface morphology of TFC membranes.

SEM and AFM images of COF-based TFC membranes synthesized under identical conditions but with different deposition times: (a, TFC 1) 2.0 hours, (b, TFC 2) 8.0 hours, and (c, TFC 3) 24.0 hours. The results demonstrate that extending the reaction time initially improves surface coverage and smoothness (up to 8.0 hours), while further prolongation to 24.0 hours leads to coarse particle deposition, increased surface roughness, and crack formation due to excessive bulk nucleation and growth.

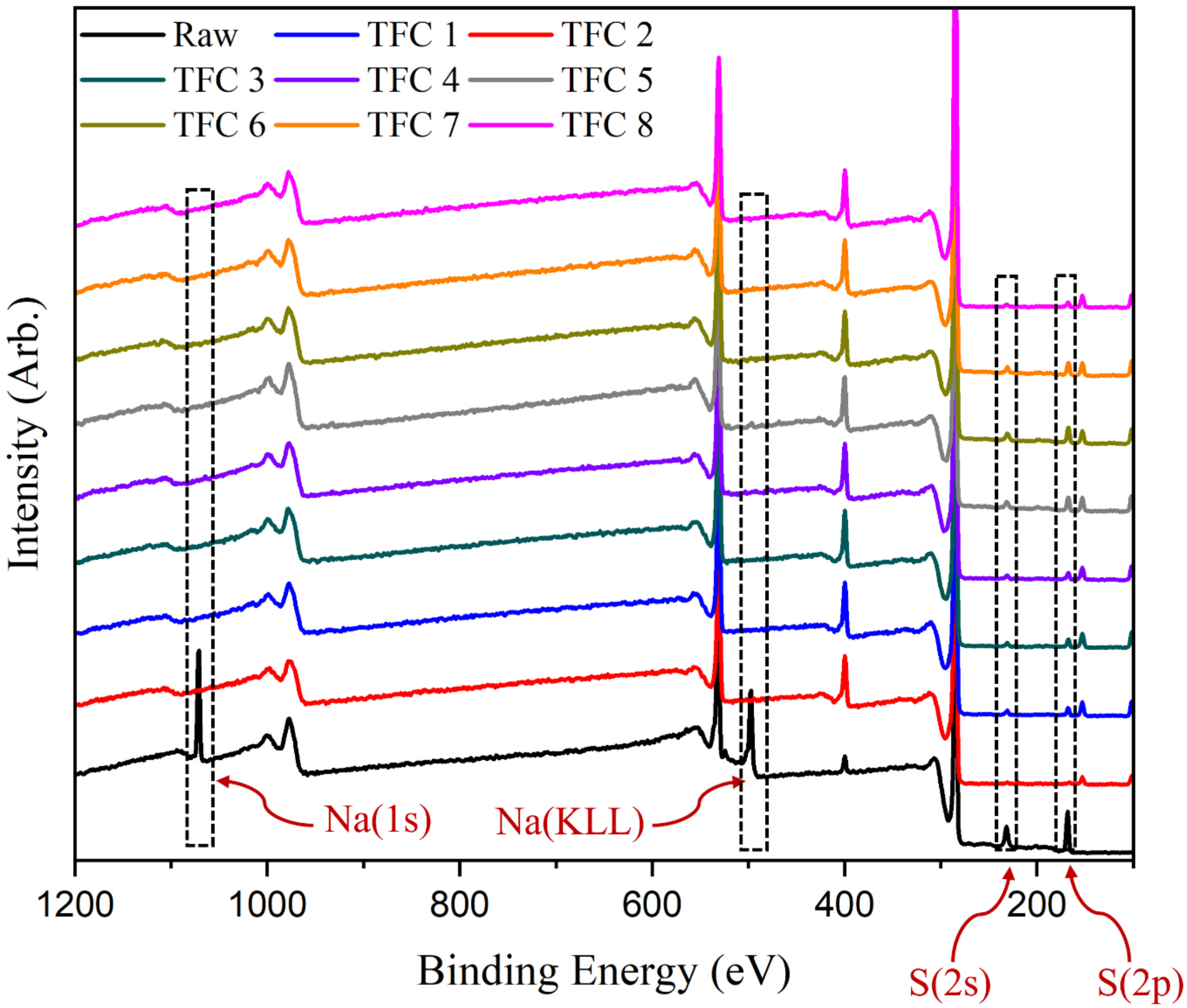

Figure S17. Effect of fabrication parameters on the structural integrity of the TFC membranes.

XPS survey spectra of the COF-based TFC membranes. The attenuation of the sulfur (S 2s, S 2p) and sodium (Na 1s, Na KLL) signals originating from the CEM support (pristine) indicates the formation of a continuous COF coating. Comparison of the spectra, together with the quantitative results summarized in Table S7, confirms that sample TFC 2, synthesized under AC conditions (8.0 h, 10.0 Vpp, Square waveform, 1.0 mHz), exhibits the highest surface coverage and best coating quality.

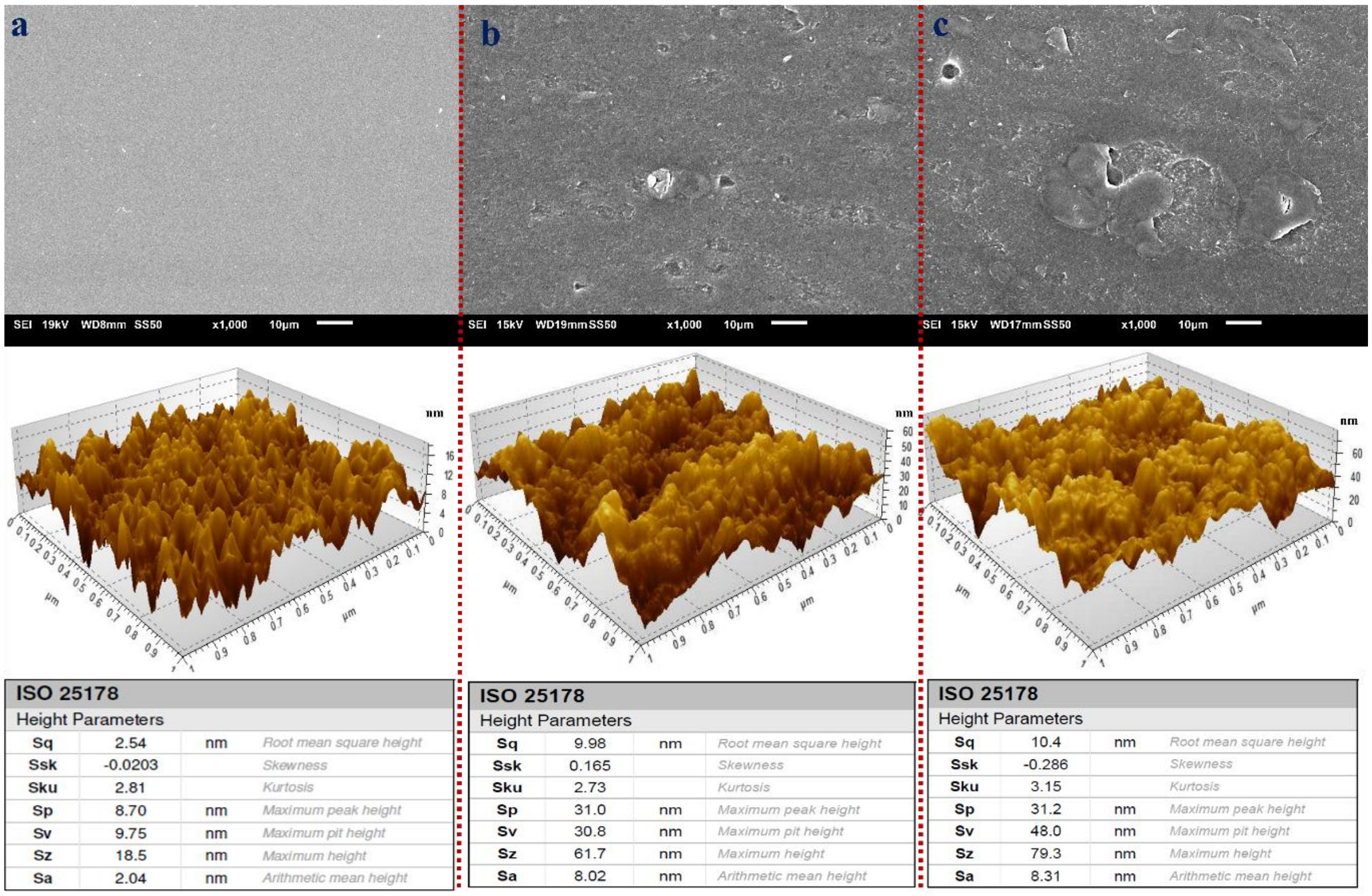

Figure S18. Effect of the waveform on the surface morphology of TFC membranes.

SEM and AFM images of COF-based TFC membranes synthesized under identical conditions using different types of applied AC voltages: (a, TFC 2) square waveform, (b, TFC 4) sinusoidal waveform, and (c, TFC 5) ramp waveform. The results demonstrate that the square waveform yields the most uniform and defect-free surface morphology, whereas sinusoidal and ramp waveforms lead to comparatively rougher and less homogeneous coatings.

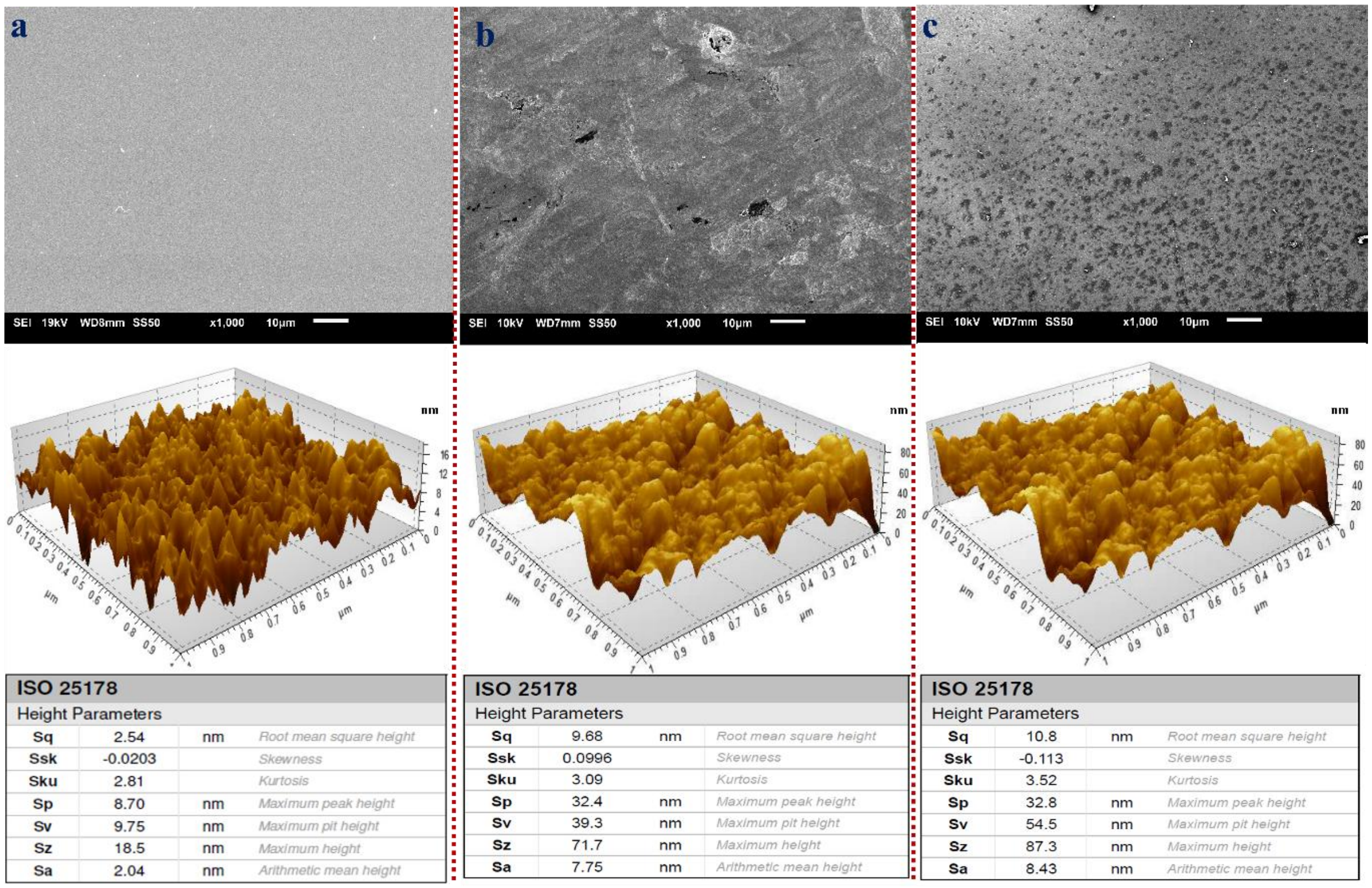

Figure S19. Effect of the electric-field frequency on the surface morphology of TFC membranes.

SEM and AFM images of COF-based TFC membranes synthesized under identical conditions but with different AC field frequencies: (a, TFC 2) 1.0 mHz, (b, TFC 9) 1.0 Hz, and (c, TFC 10) 1000.0 Hz. The results reveal that low frequencies (1.0 mHz) promote uniform and continuous COF film formation, whereas higher frequencies lead to non-uniform deposition and increased surface roughness. This trend reflects the timescale of interfacial hydronium delivery: at low frequency, the half-cycle is long enough for the hydronium ions generated by water splitting to migrate across the CEM and reach the monomer-enriched region, establishing the localized acidic environment required for interfacial condensation; at higher frequencies, the field polarity reverses before this transport is complete, so the interfacial region is never adequately acidified despite the unchanged time-averaged generation rate.

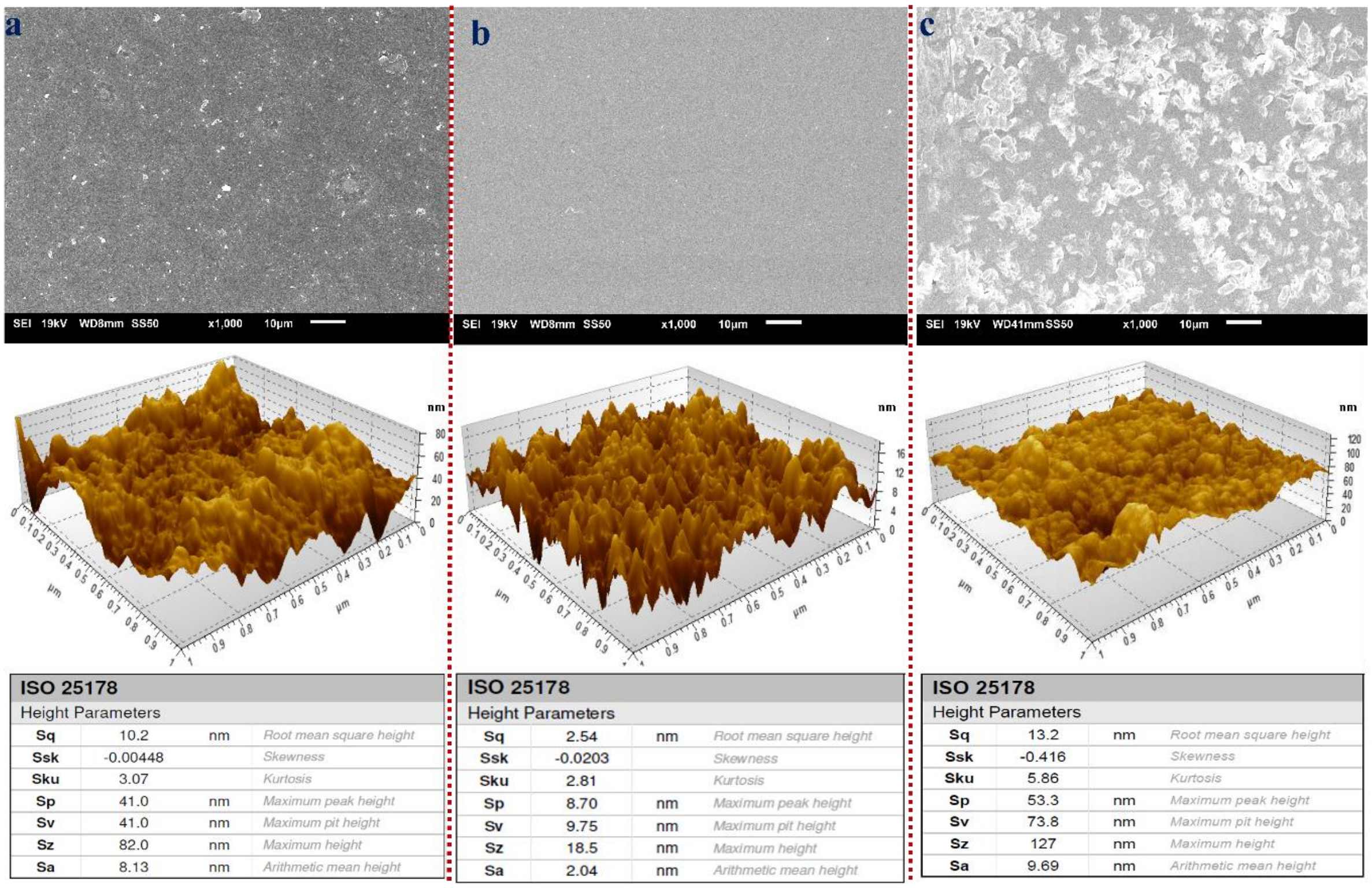

a

ISO 25178	Height Parameters		
Sq	10.2	nm	Root mean square height
Ssk	-0.00448		Skewness
Sku	3.07		Kurtosis
Sp	41.0	nm	Maximum peak height
Sv	41.0	nm	Maximum pit height
Sz	82.0	nm	Maximum height
Sa	8.13	nm	Arithmetic mean height

b

ISO 25178	Height Parameters		
Sq	2.54	nm	Root mean square height
Ssk	-0.0203		Skewness
Sku	2.81		Kurtosis
Sp	8.70	nm	Maximum peak height
Sv	9.75	nm	Maximum pit height
Sz	18.5	nm	Maximum height
Sa	2.04	nm	Arithmetic mean height

c

ISO 25178	Height Parameters		
Sq	13.2	nm	Root mean square height
Ssk	-0.416		Skewness
Sku	5.86		Kurtosis
Sp	53.3	nm	Maximum peak height
Sv	73.8	nm	Maximum pit height
Sz	127	nm	Maximum height
Sa	9.69	nm	Arithmetic mean height

Figure S20. Effect of the electric-field intensity on the surface morphology of TFC membranes.

SEM and AFM images of COF-based TFC membranes synthesized under identical conditions but with different applied AC voltages: (a, TFC 6) 2.0 Vpp, (b, TFC 2) 10.0 Vpp, and (c, TFC 7) 20.0 Vpp. Moderate amplitudes (10.0 Vpp) yield the most uniform and compact COF layers. At low amplitude (2.0 Vpp), the potential does not exceed the water-splitting threshold, so little hydronium is generated and the interfacial acidity required for condensation is not established, resulting in incomplete surface coverage despite the electrophoretic driving force on the monomers remaining operative. At high amplitude (20.0 Vpp), excessive water splitting generates an overly acidic interfacial microenvironment and enhanced hydrogen/oxygen gas evolution, which together promote coarse particle aggregation, surface roughening, and crack formation. These results indicate that the optimal amplitude lies just above the water-splitting threshold, providing steady hydronium generation without inducing harsh reaction conditions or gas accumulation.

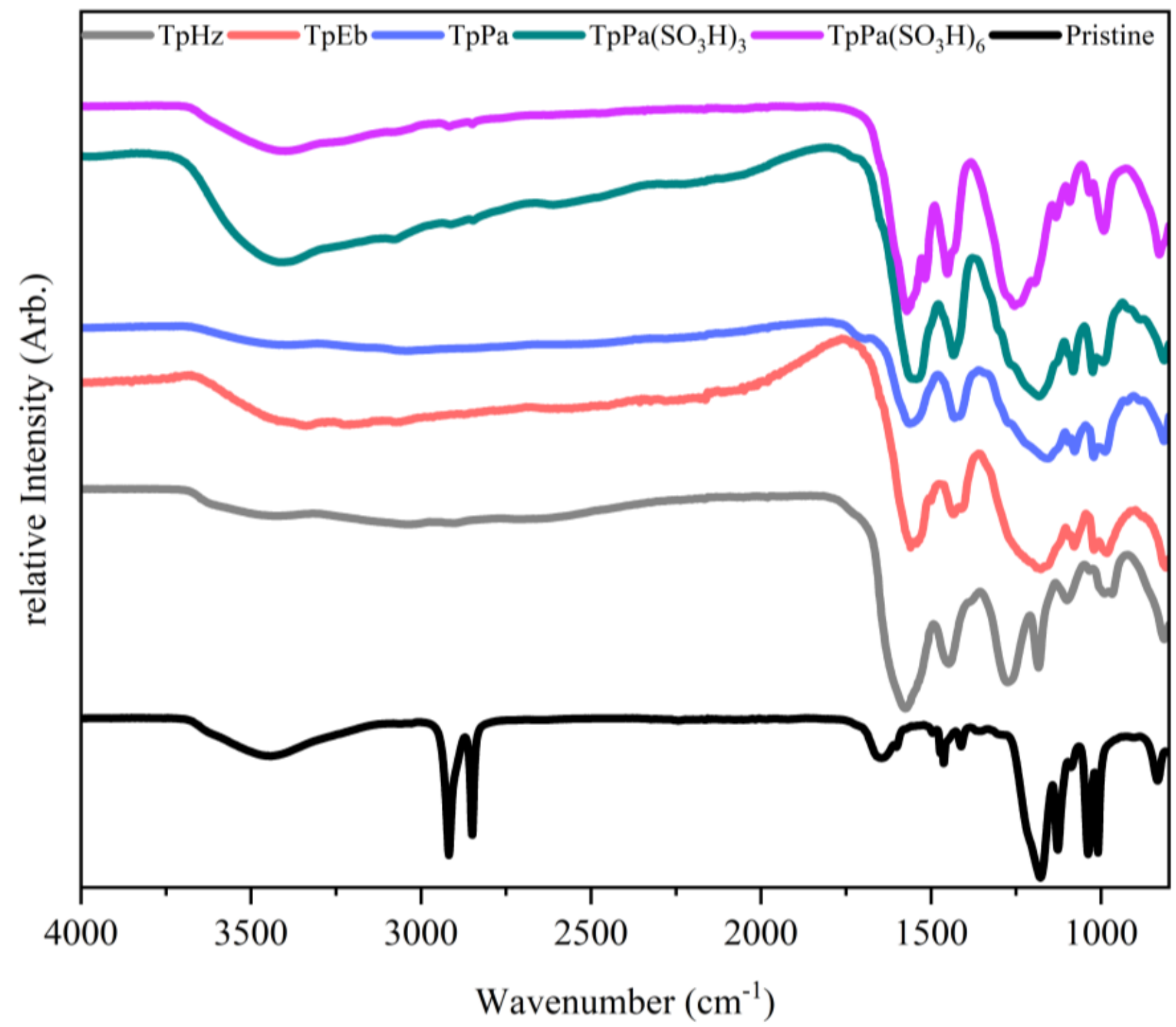

Figure S21. FTIR evidence for COF formation and for complete surface coverage of the optimized membrane.

FTIR spectra of the different COF-based TFC membranes. The characteristic C-N stretching bands observed between 1200 and 1250 cm^{-1} and the C=C stretching band near 1590 cm^{-1}, together with the disappearance of the $-NH_2$ double peaks around 3400 cm^{-1} (originating from amine-containing monomers) and the C=O band near 1640 cm^{-1} (from aldehyde-containing monomers), confirm the successful formation of various ionic and neutral COF structures.

The aliphatic C-H stretching bands at 2850-2950 cm^{-1} arise from the polyolefin backbone of the sulfonated support and are absent from the aromatic COF frameworks; they therefore serve as a marker for exposed (uncovered) support. For the optimized TpEb membrane (TFC 2) these bands are eliminated entirely, indicating that the coating attenuates the underlying support signal completely over the sampled region. In contrast, weak residual C-H bands persist for the anionic frameworks $TpPa(SO_3H)_3$ and $TpPa(SO_3H)_6$, in which coating and support carry like charge. The complete elimination of the support signal specifically for the cationic TpEb layer; electrostatically attracted to the sulfonated support; provides an internal control confirming that the technique retains sensitivity to incomplete coverage and reports its absence only for the optimized system.

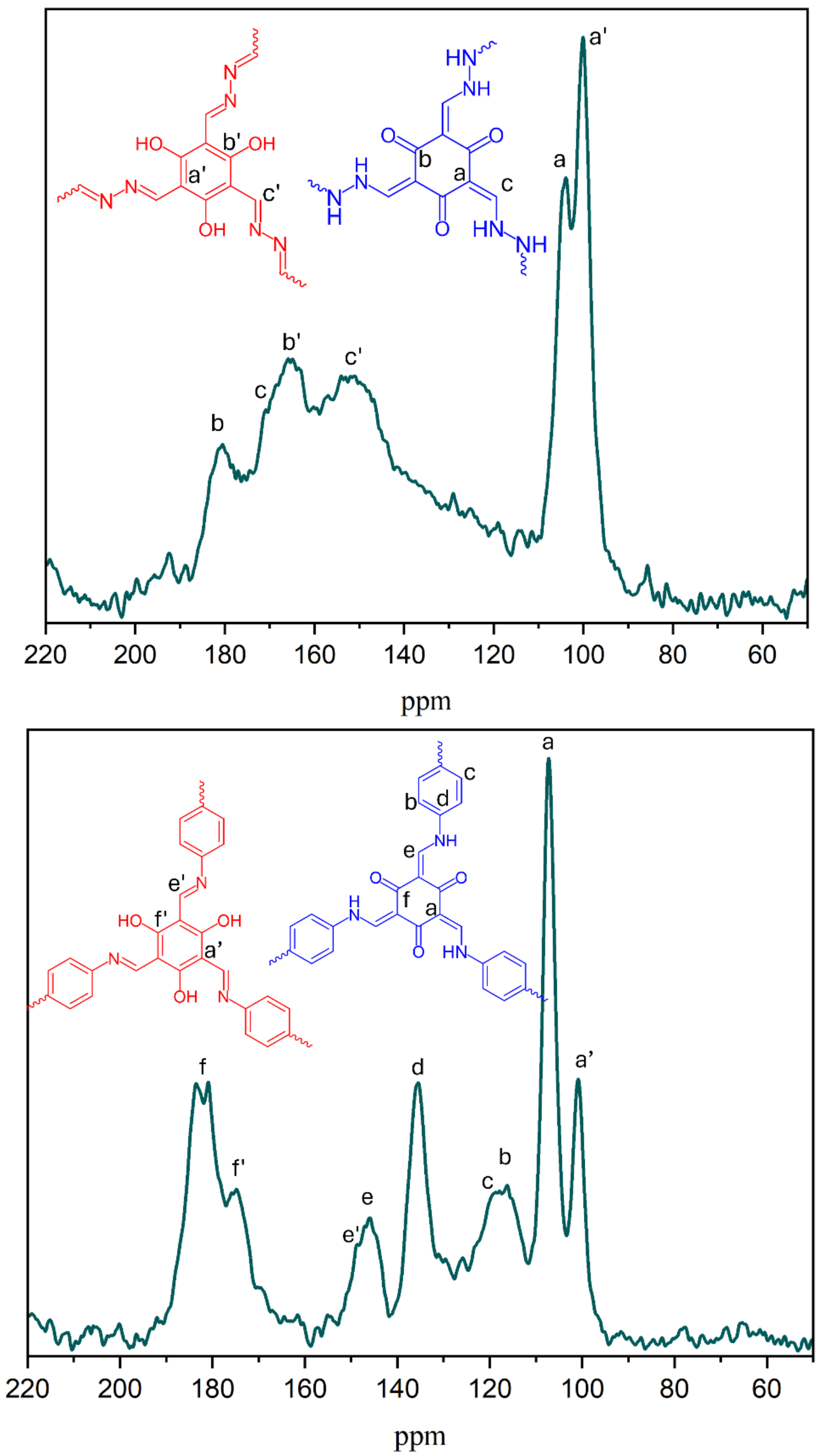

Figure S22. Presence of tautomeric equilibrium in the synthesized COFs.

Solid-state ^{13}CNMR spectra of TpHz (TFC 17) and TpPa (TFC 16) COF layers deposited on CEM supports. Peak assignments confirm the coexistence of both enol and keto tautomeric forms within the resulting COF networks, indicating that the imine/enamine equilibrium persists under the applied fabrication conditions[33].

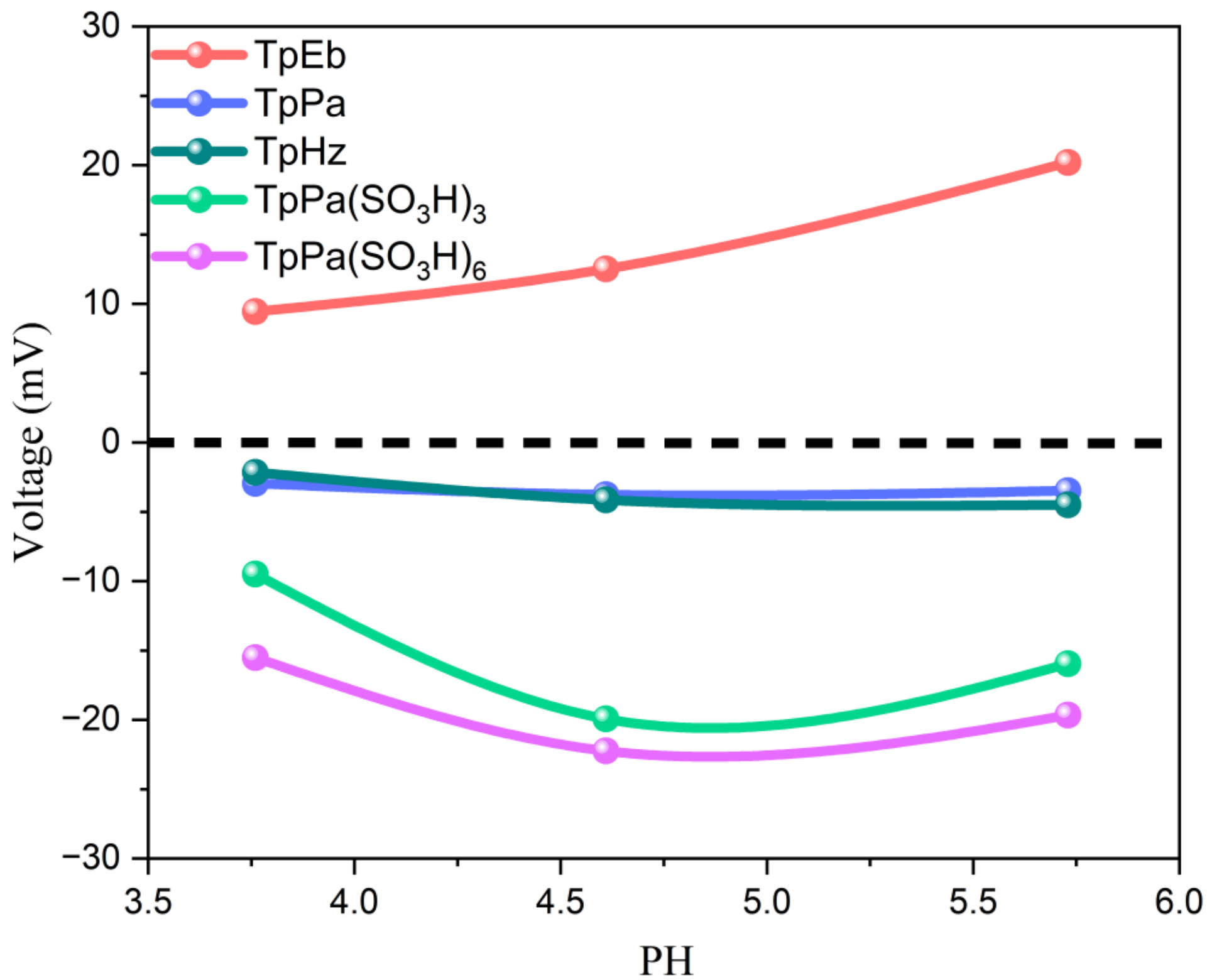

Figure S23. Surface charge of synthesized COFs.

Zeta-potential measurements of different COFs synthesized in the work. While the positive surface potential of TpEb and the negative potential of sulfonated TpPa COFs were anticipated, a slightly negative zeta potential was unexpectedly observed for the TpPa and TpHz COFs.

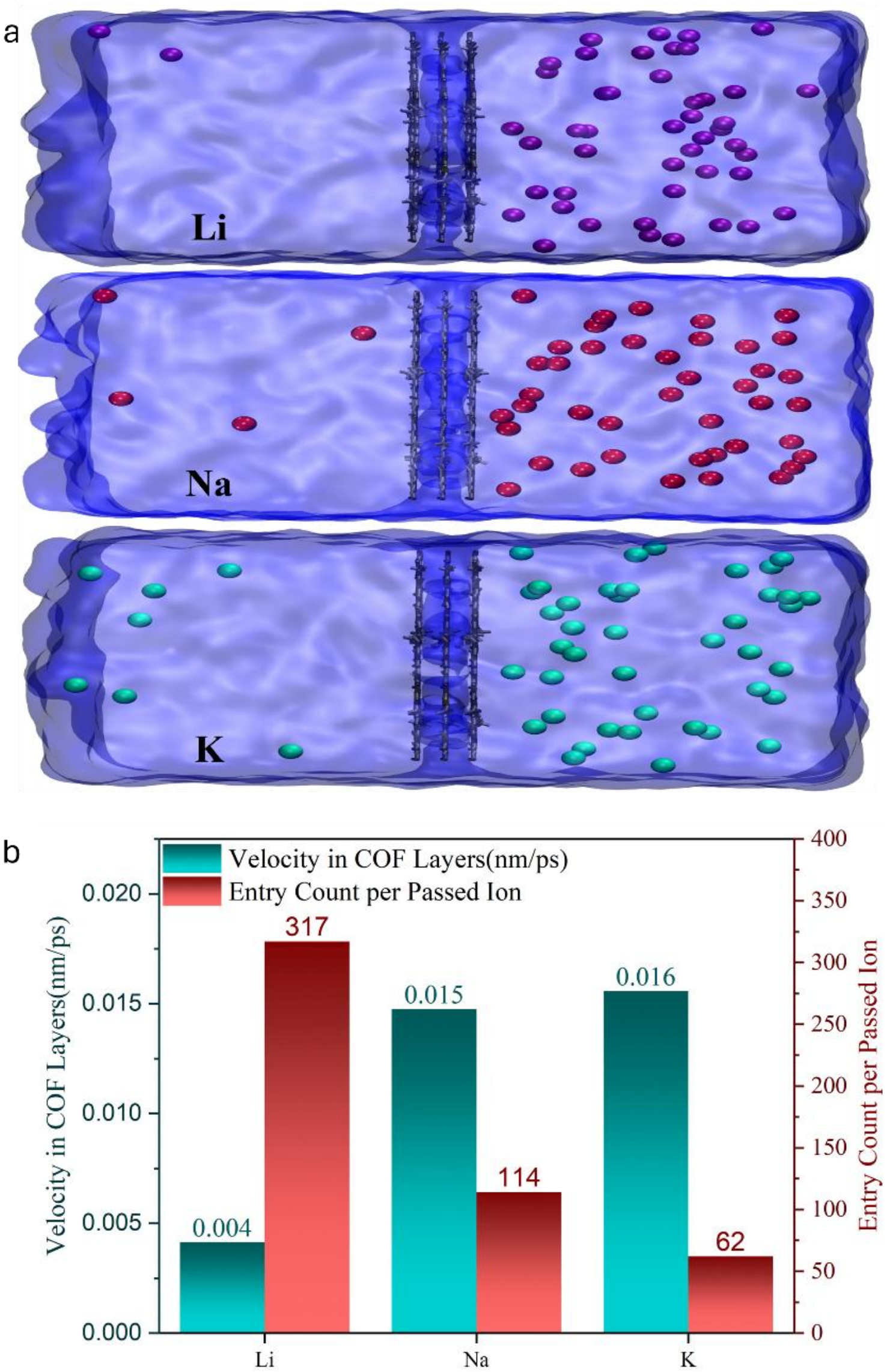

Figure S24. Feasibility of monovalent cation transport in the TpEb COF layers.

Molecular dynamics (MD) simulation results illustrating ion-specific transport behavior through the COF layer. (a) Representative snapshots of the final simulation frames for different monovalent ions, highlighting the number of ions that successfully permeate the COF pore, consistent with the ion discrimination trends observed experimentally (Figure 4). (b) Ion velocities within the COF region and the frequency of pore-entry “attempts” per successful permeation event. Li^+ requires approximately 317 entry attempts for each successful translocation, compared with 114 attempts for Na^+ and 62 attempts for K^+, indicating a very low commitment probability for Li^+ at the pore entrance. Furthermore, even after entering the pore, the average transit velocity of Li^+ is approximately four times lower than that of Na^+ and K^+, consistent with the higher energetic cost associated with dehydration and the transport of its tightly bound, structurally rigid hydration shell.

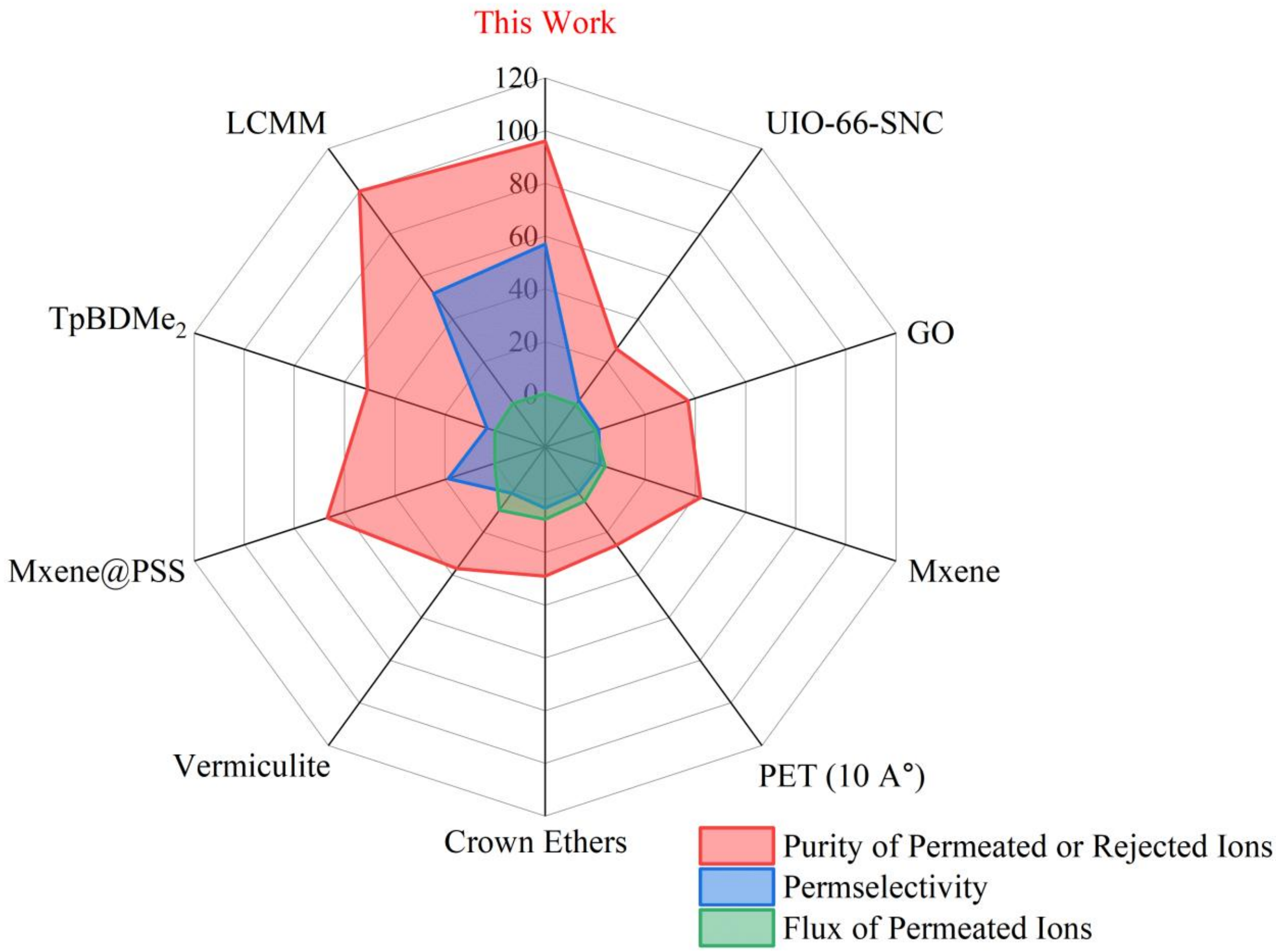

Figure S25. Comparison of separation performance.

Spider plot comparing permselectivity, ion flux, and the purity of permeated/rejected ions for binary mixtures of Li, Na, and K ions (LCMM: liquid-crystalline MXene membranes; GO: graphene oxide; PSS: poly(sodium 4-styrene sulfonate); PET: polyethylene terephthalate; SNC: sub-nanochannel) [34–45]. The defect-free TpEb-coated CEM (TFC 2) developed in this work exhibits remarkably high permselectivity (65 for K/Li, 61 for K/Na, and 43 for Li/Na) and a high ion flux (0.13 mol m^{-2} h^{-1}), enabling highly efficient separation of K/Li and K/Na (near-complete) and Li/Na (92%).

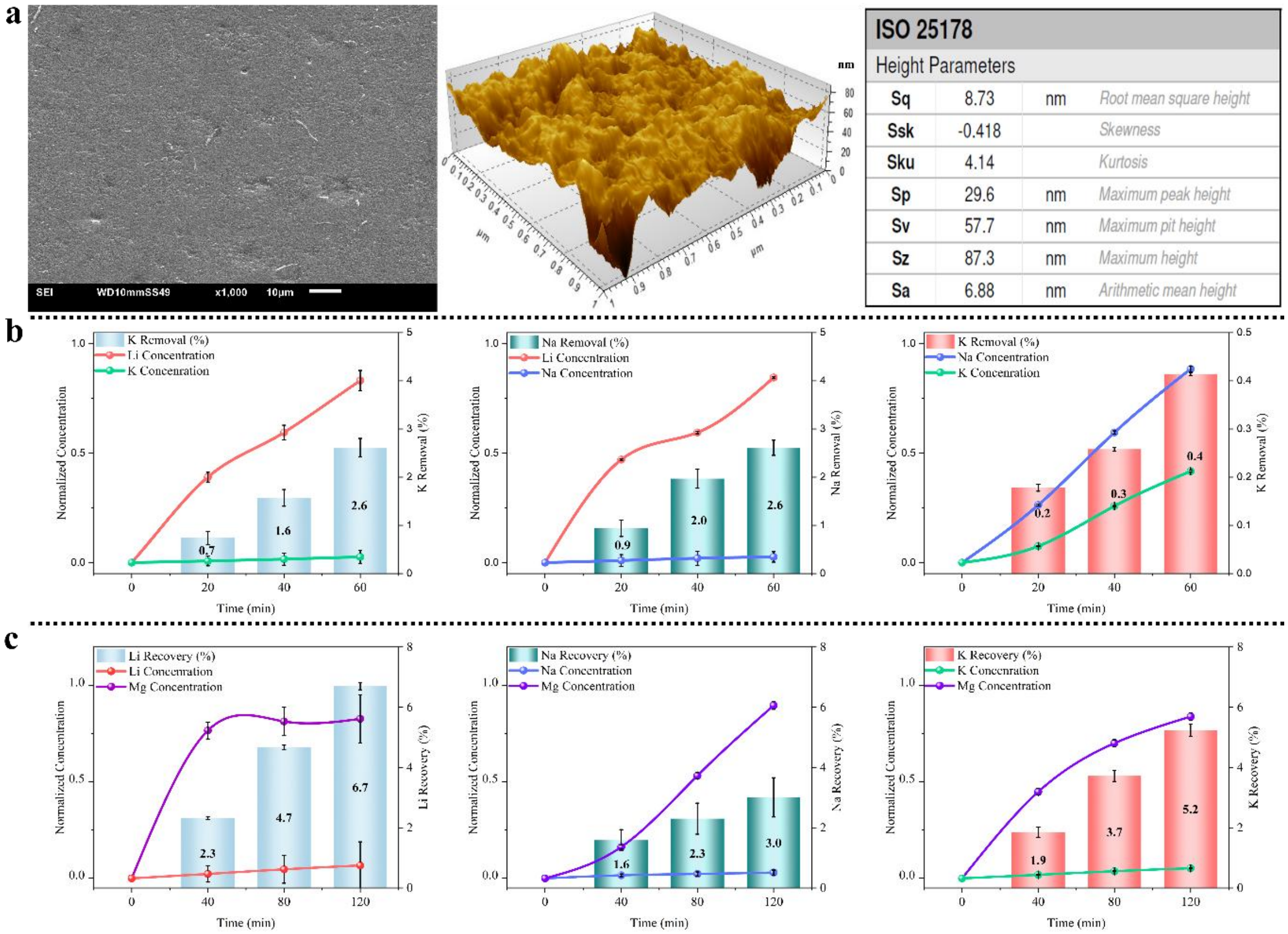

Figure S26. Surface morphology and ED performance evaluation of the pristine CEM used as the support in this study.

(a) SEM and AFM images of the pristine CEM surface. (b) ED separation performance of the pristine membrane for mono/divalent ion mixtures with feed compositions of $MgCl_2$ (45.0 mM)/MCl (5.0 mM), where M = Li^+, Na^+, or K^+. ED operating conditions: constant applied voltage of 1.5 V, initial current of approximately 2.0 mA, and operation time of 120.0 min. (c) ED separation performance of the pristine membrane for mono/monovalent ion mixtures with feed compositions of M_1Cl (45.0 mM)/M_2Cl (5.0 mM), where M_1= Li^+ or Na^+ and M_2 = Na^+ or K^+. ED operating conditions: constant applied voltage of 1.0 V, initial current of approximately 1.0 mA, and operation time of 60.0 min.

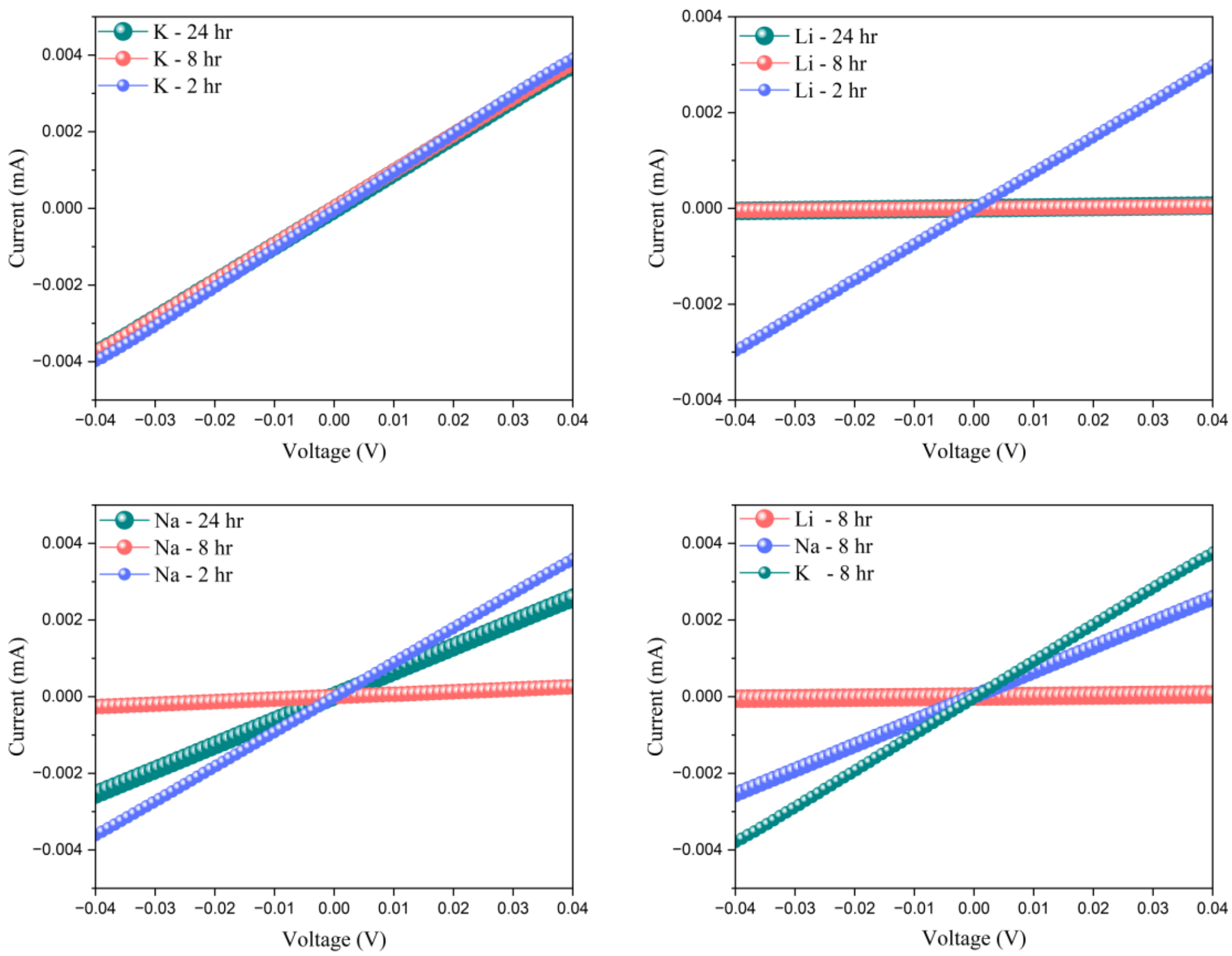

Figure S27. Effect of the duration of deposition on the ionic conductance.

Current-voltage (I-V) curves showing the effect of deposition time on the ionic conductance of different alkali metal ions through the TpEb-based TFC membranes (24.0 hours: TFC 3, 8.0 hours: TFC 2 and 2.0 hours: TFC 1).

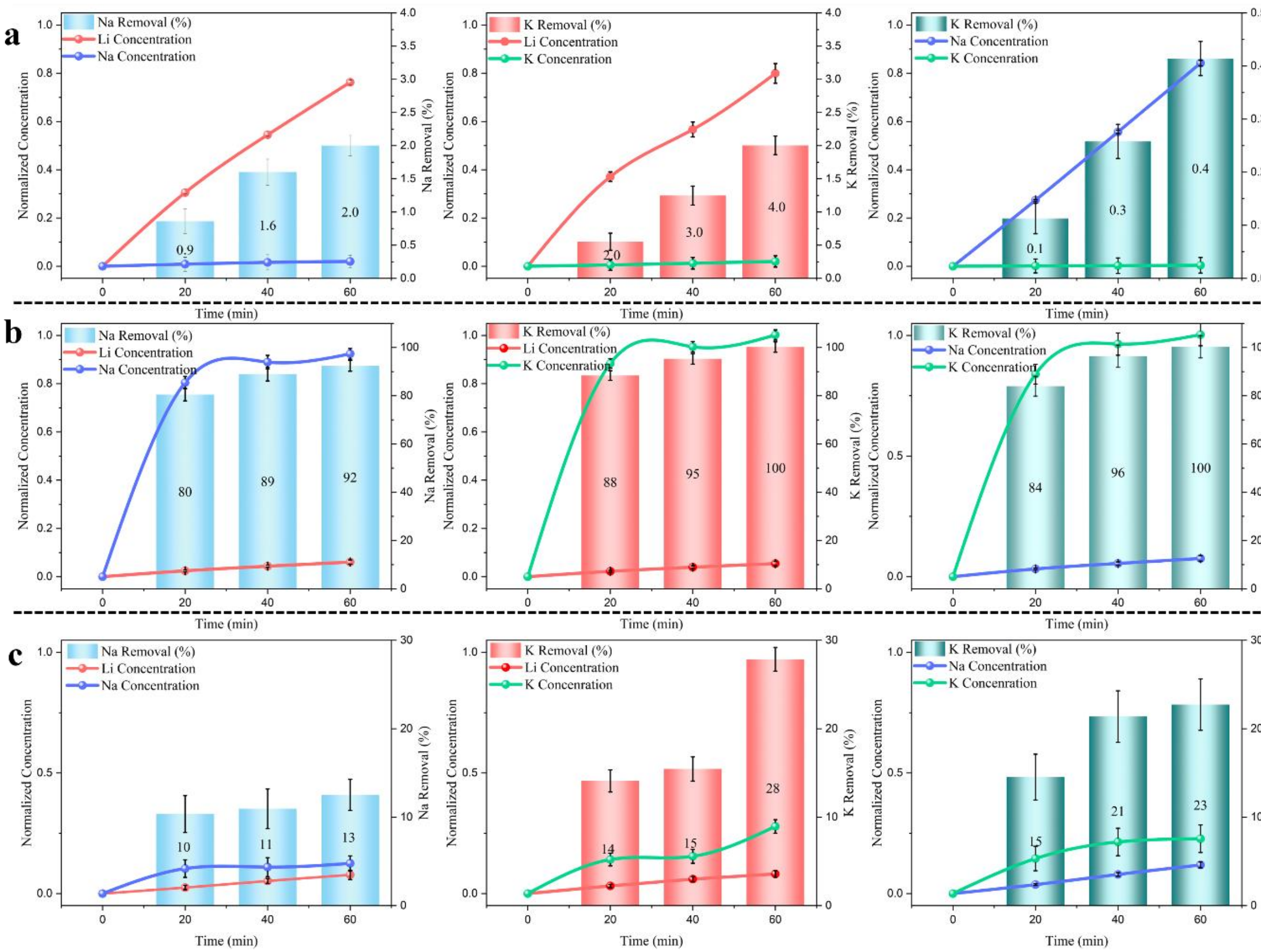

Figure S28. Effect of the duration of deposition on the separation performance.

Full-scan electrodialysis results (for mono/monovalent ion mixtures with feed compositions of M_1Cl (45.0 mM)/M_2Cl (5.0 mM), where M_1= Li^+ or Na^+ and M_2 = Na^+ or K^+. ED operating conditions: constant applied voltage of 1.0 V, initial current of approximately 1.0 mA, and operation time of 60.0 min.) showing the effect of deposition time on the separation performance of TpEb-based TFC membranes for Li/Na, Li/K, and Na/K systems: (a, TFC 1) 2.0 h, (b, TFC 2) 8.0 h, and (c, TFC 3) 24.0 h. These results clearly demonstrate that deposition time plays a crucial role in determining membrane performance. At very short deposition times (2 h; a), the self-healing and self-regulating growth mechanism does not have sufficient time to bridge the gaps between primary COF particles, resulting in poor surface coverage. Conversely, at very long deposition times (24.0 h; c), excessive film thickening and the incorporation of coarse COF aggregates can introduce stress concentration points within the membrane. These defects increase the likelihood of crack initiation and propagation during post-fabrication handling. By selecting an appropriate intermediate deposition time (8.0 h; b), the formation of a uniform, crack-free COF layer is favored, enabling near-complete ion-separation efficiency.

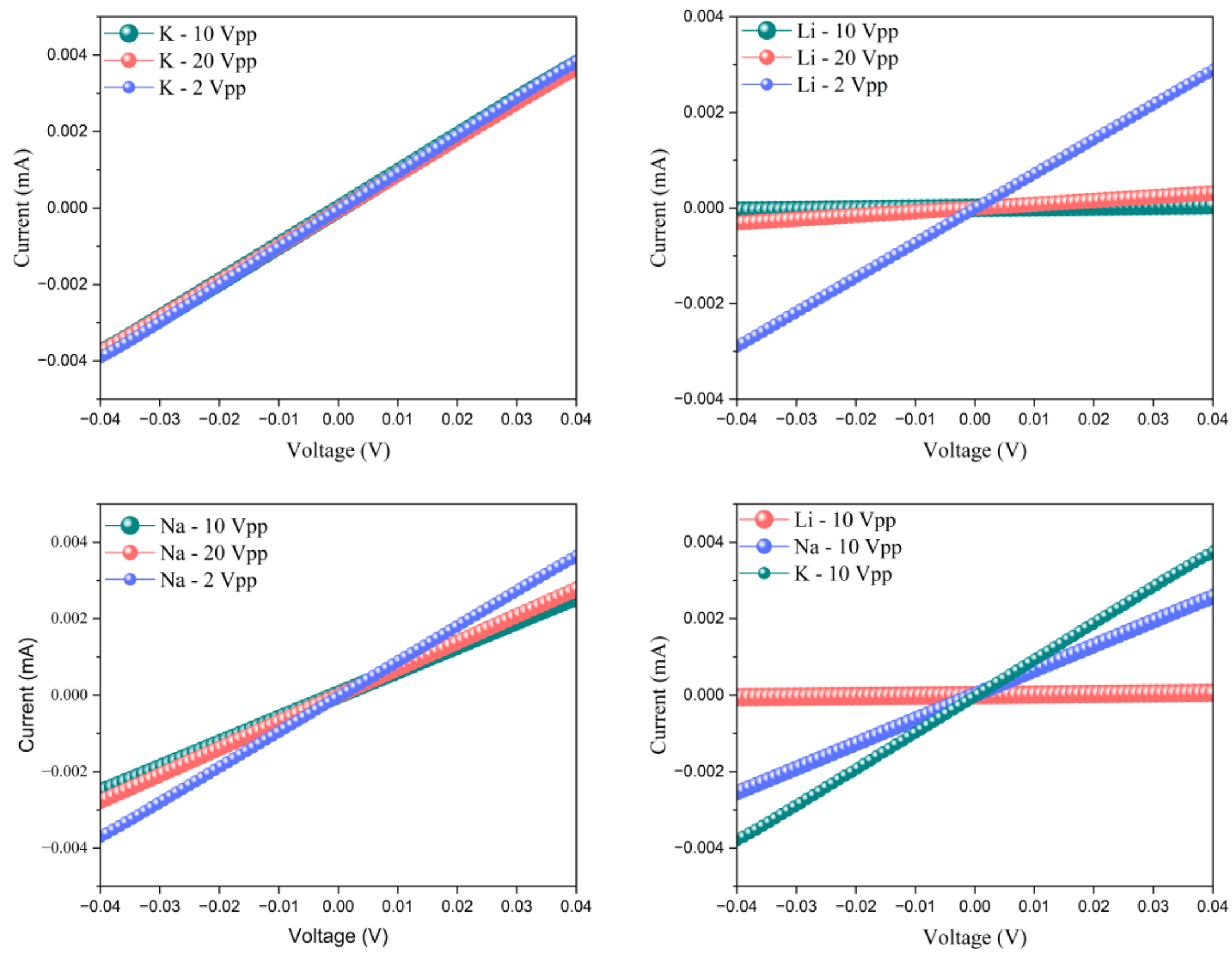

Figure S29. Effect of the voltage intensity on the ionic conductance.

Current-voltage (I-V) curves showing the effect of deposition voltage on the ionic conductance of different alkali metal ions through the TpEb-based TFC membranes (10.0 Vpp: TFC 2, 20.0 Vpp: TFC 7, and 2.0 Vpp: TFC 6).

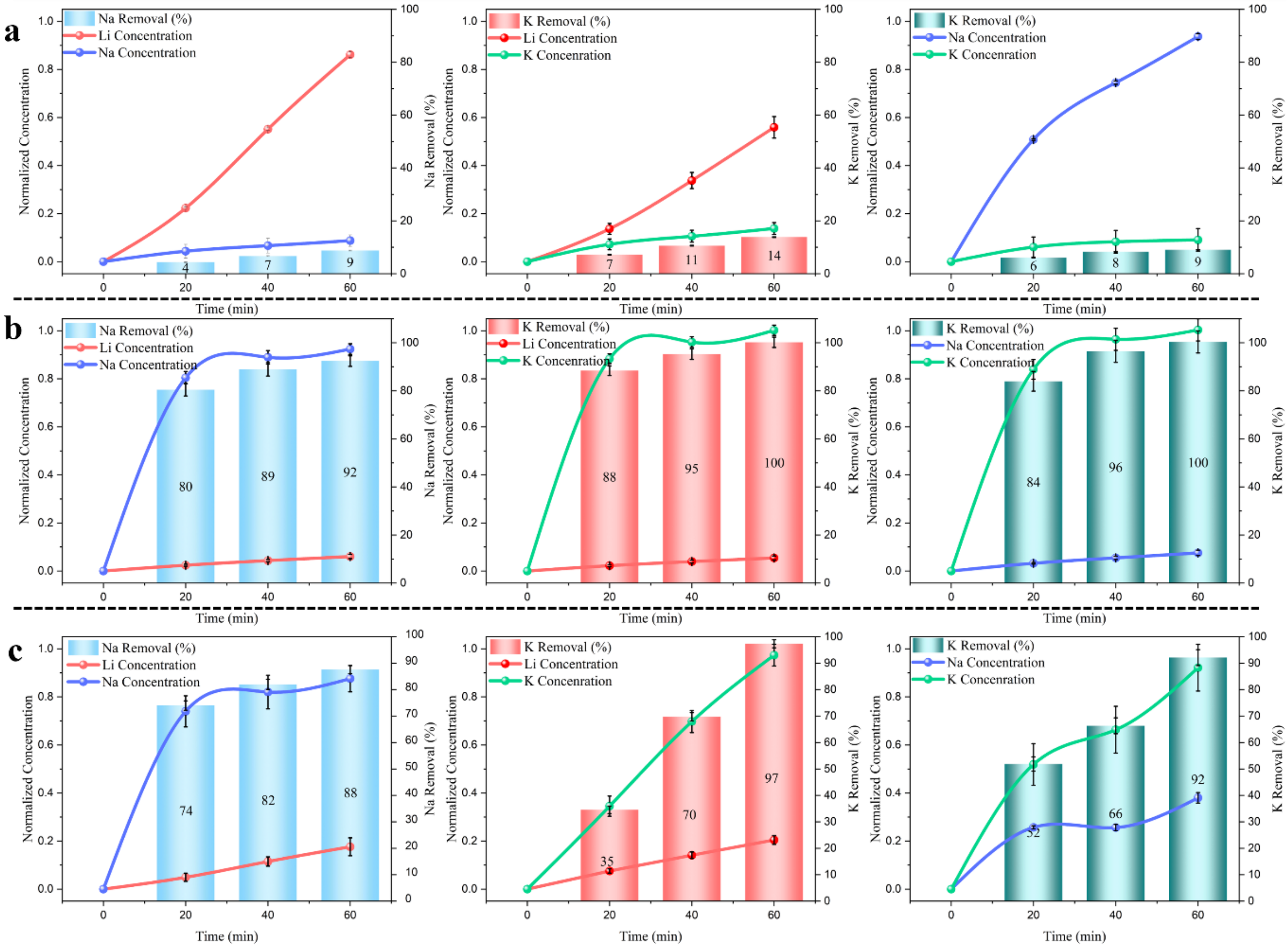

Figure S30. Effect of the voltage intensity on the separation performance.

Full-scan electrodialysis results (for mono/monovalent ion mixtures with feed compositions of M_1Cl (45.0 mM)/M_2Cl (5.0 mM), where M_1= Li^+ or Na^+ and M_2 = Na^+ or K^+. ED operating conditions: constant applied voltage of 1.0 V, initial current of approximately 1.0 mA, and operation time of 60.0 min.) showing the effect of deposition voltage on the separation performance of TpEb-based TFC membranes for Li/Na, Li/K, and Na/K systems: (a, TFC 6) 2.0 Vpp, (b, TFC 2) 10.0 Vpp, and (c, TFC 7) 20.0 Vpp. These results clearly demonstrate that the deposition voltage plays a crucial role in determining the performance of the TFC membranes. At very low voltages (a, 2.0 Vpp), which are below the threshold for water splitting, hydronium production is insufficient and the electrophoretic migration of the COF-forming monomers is weak. This results in poor surface coverage. Conversely, at excessively high voltages (c, 20.0 Vpp), the enhanced reaction rate is accompanied by significant gas generation and accumulation, which induces mechanical stress and can damage the TFC membrane. By selecting an appropriate intermediate voltage (b, 10.0 Vpp), the likelihood of forming a homogeneous, crack-free COF layer is greatly increased, enabling near-complete ion-separation efficiency.

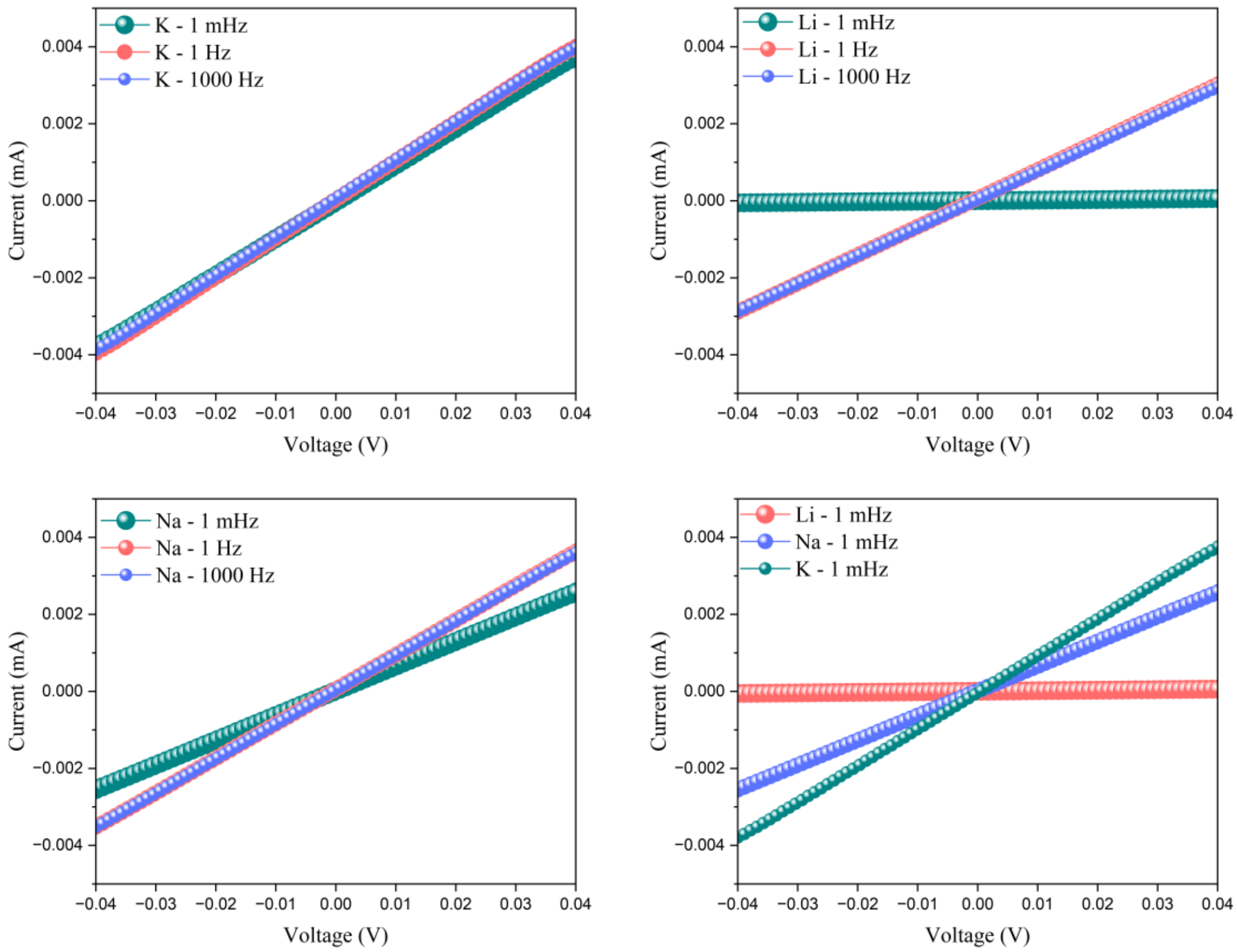

Figure S31. Effect of the voltage frequency on the ionic conductance.

Current-voltage (I-V) curves showing the effect of deposition frequency on the ionic conductance of different alkali metal ions through the TpEb-based TFC membranes (1.0 mHz: TFC 2, 1.0 Hz: TFC 9, and 1000.0 Hz: TFC 10).

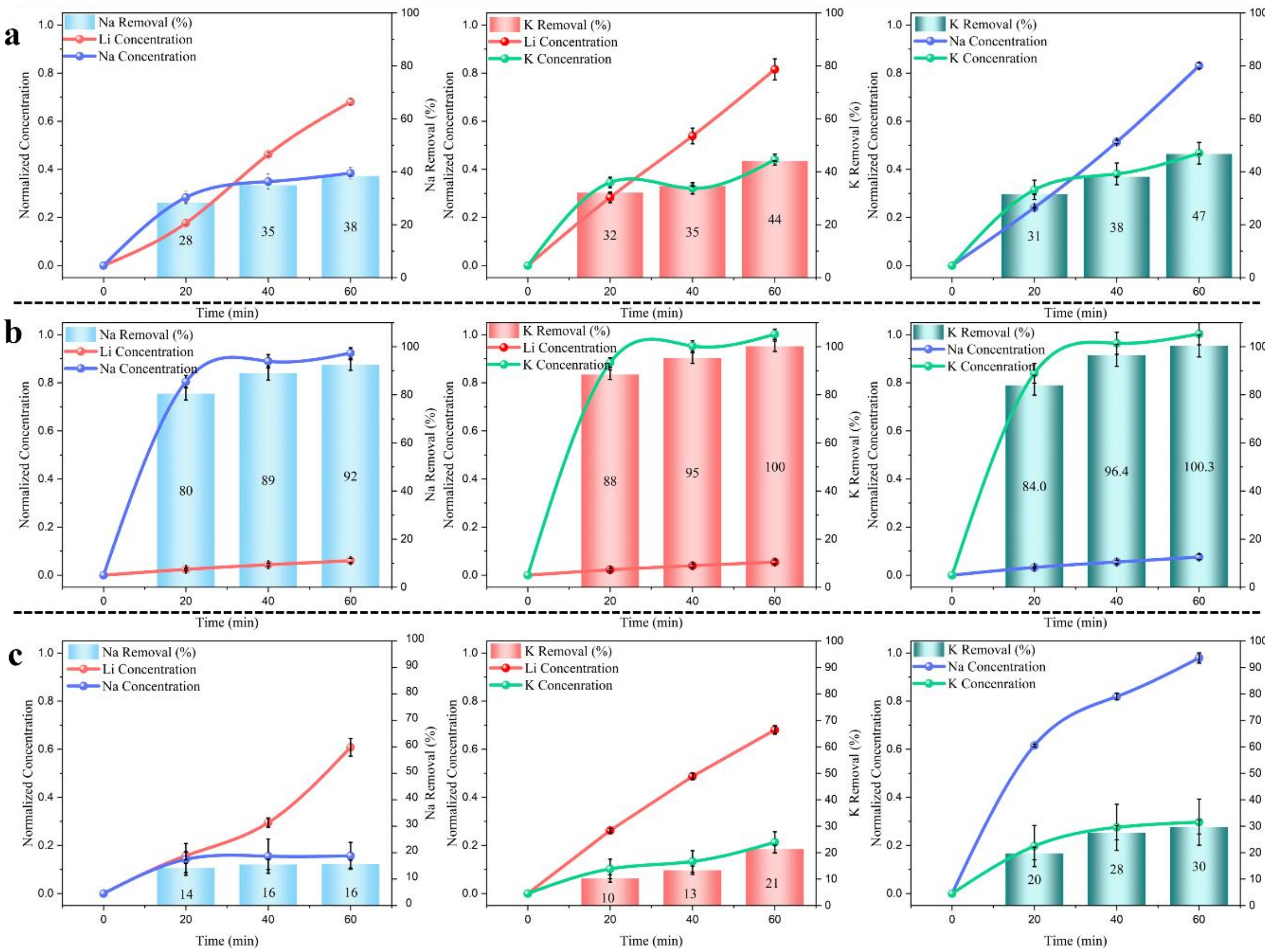

Figure S32. Effect of the voltage frequency on the separation performance.

Full-scan electrodialysis results (for mono/monovalent ion mixtures with feed compositions of M_1Cl (45.0 mM)/M_2Cl (5.0 mM), where $M_1 = Li^+$ or Na^+ and $M_2 = Na^+$ or K^+. ED operating conditions: constant applied voltage of 1.0 V, initial current of approximately 1.0 mA, and operation time of 60.0 min.) showing the effect of deposition voltage frequency on the separation performance of TpEb-based TFC membranes for Li/Na, Li/K, and Na/K systems: (a, TFC 9) 1.0 Hz, (b, TFC 2) 1.0 mHz, and (c, TFC 10) 1000.0 Hz. These results clearly demonstrate that the deposition voltage frequency plays a crucial role in determining membrane performance. At high frequencies (1.0 and 1000.0 Hz; a and c), the half-cycle is too short for the hydronium ions generated by water splitting to migrate across the CEM and reach the monomer-enriched region before the polarity reverses; the interfacial region is therefore never adequately acidified, resulting in poor coverage and incomplete film formation, despite the unchanged time-averaged generation rate. In contrast, at very low frequencies (1.0 mHz; b), the longer half-cycles allow adequate hydronium transport and sustained monomer enrichment at the interface. This promotes a more uniform polymerization environment, increasing the likelihood of forming a homogeneous, crack-free COF layer and enabling near-complete ion-separation efficiency.

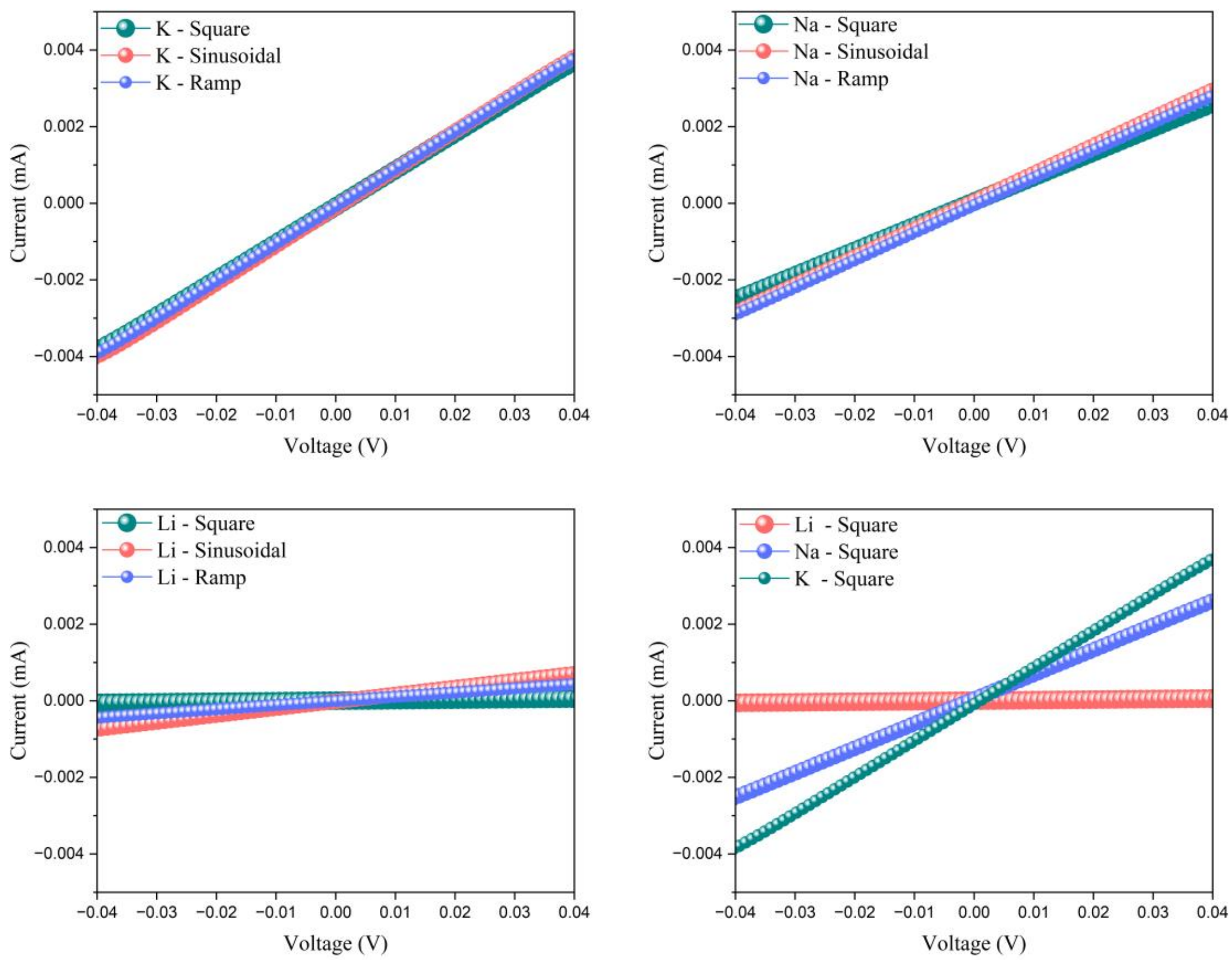

Figure S33. Effect of the waveform on the ionic conductance.

Current-voltage (I-V) curves showing the effect of the shape of applied AC voltage on the ionic conductance of different alkali metal ions through the TpEb-based TFC membranes(Square: TFC 2, Sinusoidal: TFC 4, and Ramp: TFC 5).

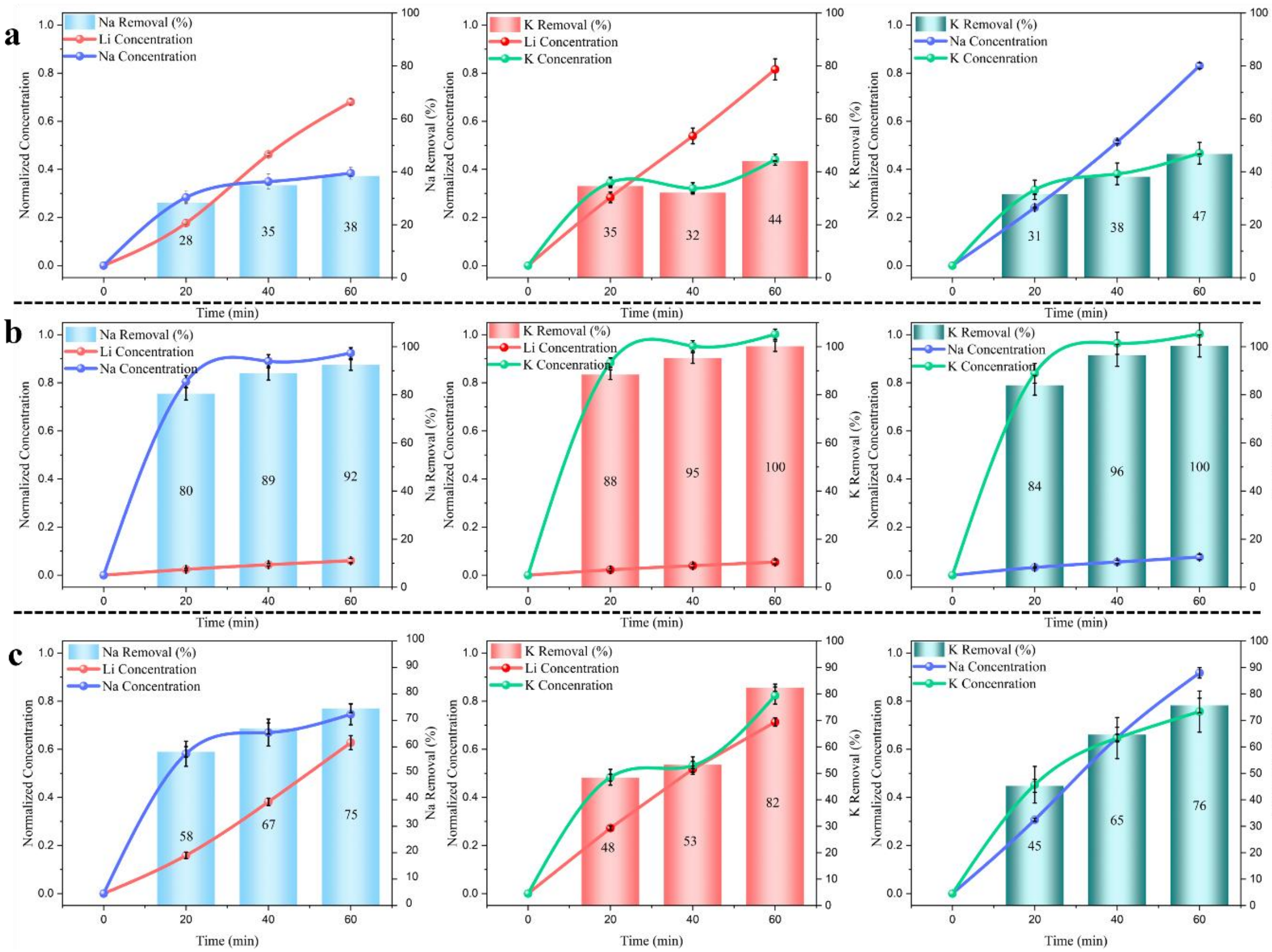

Figure S34. Effect of the waveform on separation performance.

Full-scan electrodialysis results (for mono/monovalent ion mixtures with feed compositions of M_1Cl (45.0 mM)/M_2Cl (5.0 mM), where M_1= Li^+ or Na^+ and M_2 = Na^+ or K^+. ED operating conditions: constant applied voltage of 1.0 V, initial current of approximately 1.0 mA, and operation time of 60.0 min.) showing the effect of the voltage waveform on the separation performance of TpEb-based TFC membranes for Li/Na, Li/K, and Na/K systems: (a, TFC 5) ramp, (b, TFC 2) square, and (c, TFC 4) sinusoidal. These results clearly demonstrate that the deposition waveform plays a crucial role in determining membrane performance. As shown in Figure S13 and the Supplementary Movies 1-3, the pH change at the CEM interface is most pronounced when a square waveform is applied. The stronger and more immediate supply of hydronium ions, together with a more concentrated monomer-enriched region, promotes a uniform polymerization environment. This increases the likelihood of forming a homogeneous, crack-free COF layer and achieving near-complete ion-separation efficiency. In contrast, for sinusoidal and ramp waveforms, hydronium production is delayed because the voltage rises gradually from zero to the water-splitting threshold. This time lag creates a mismatch between monomer enrichment and hydronium availability, leading to weaker film coverage and, consequently, less effective ion discrimination.

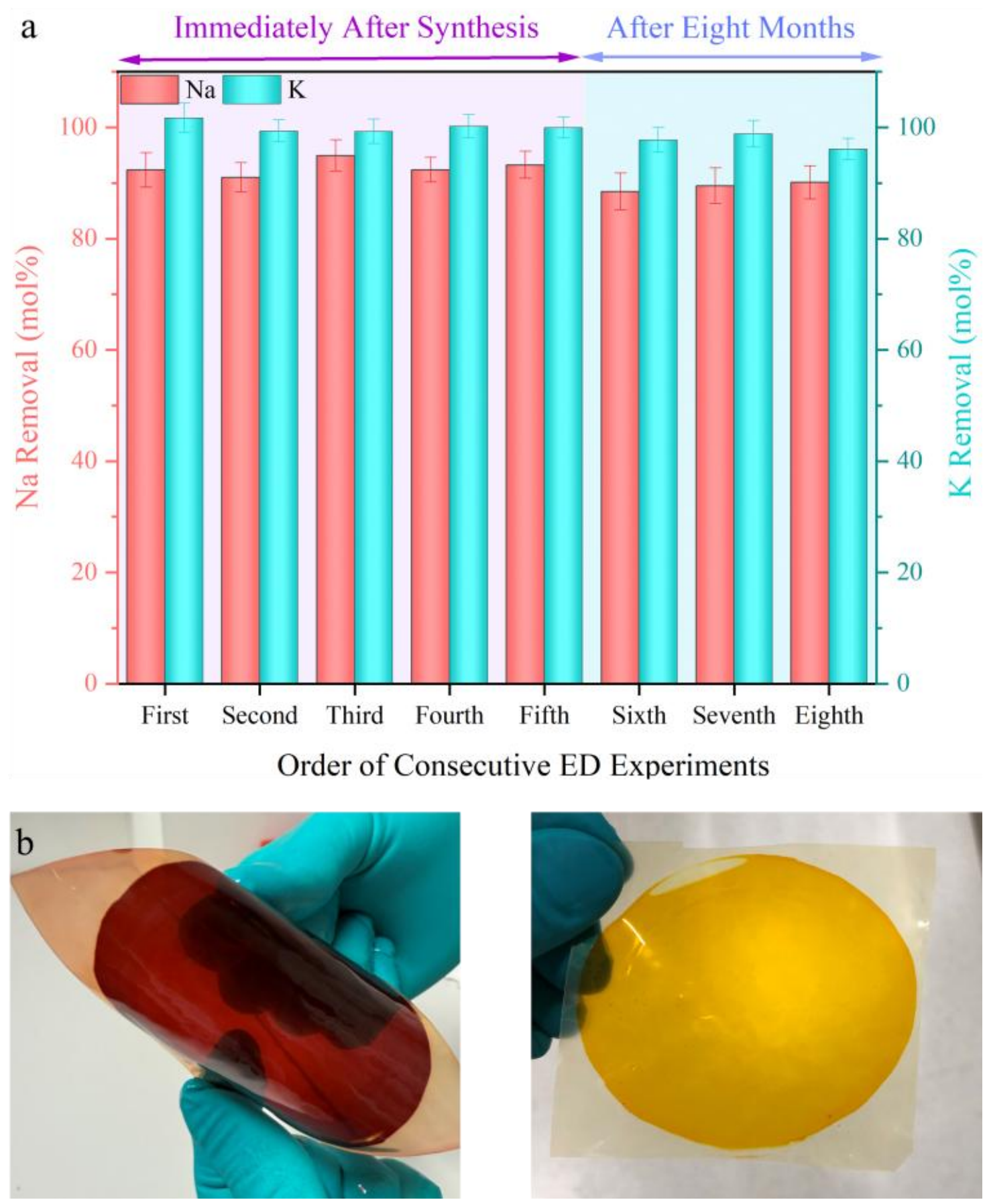

Figure S35. Stability assessment of the prepared membranes.

a) Repeated evaluation of Li/Na and Li/K separation performance for TpEb-coated CEMs (TFC 2) over eight electrodialysis cycles, demonstrating both the short- and long-term stability of the fabricated membrane. The first five cycles were performed immediately after fabrication; the remaining three were performed on the same membrane after eight months of storage in 0.1 M NaCl solution. Removal efficiencies remained consistent across all cycles, at 92 ± 2.1% for the Li/Na mixture and ~100% (below the detection limit of ICP-OES) for the Li/K mixture, indicating stable performance of the TpEb coating. b) Mechanical flexibility and structural integrity of the COF-coated CEMs after five electrodialysis experiments. Left: TpEb-coated CEMs retain their flexibility and uniform surface coverage. Right: $TpPa(SO_3H)_6$-coated CEMs.

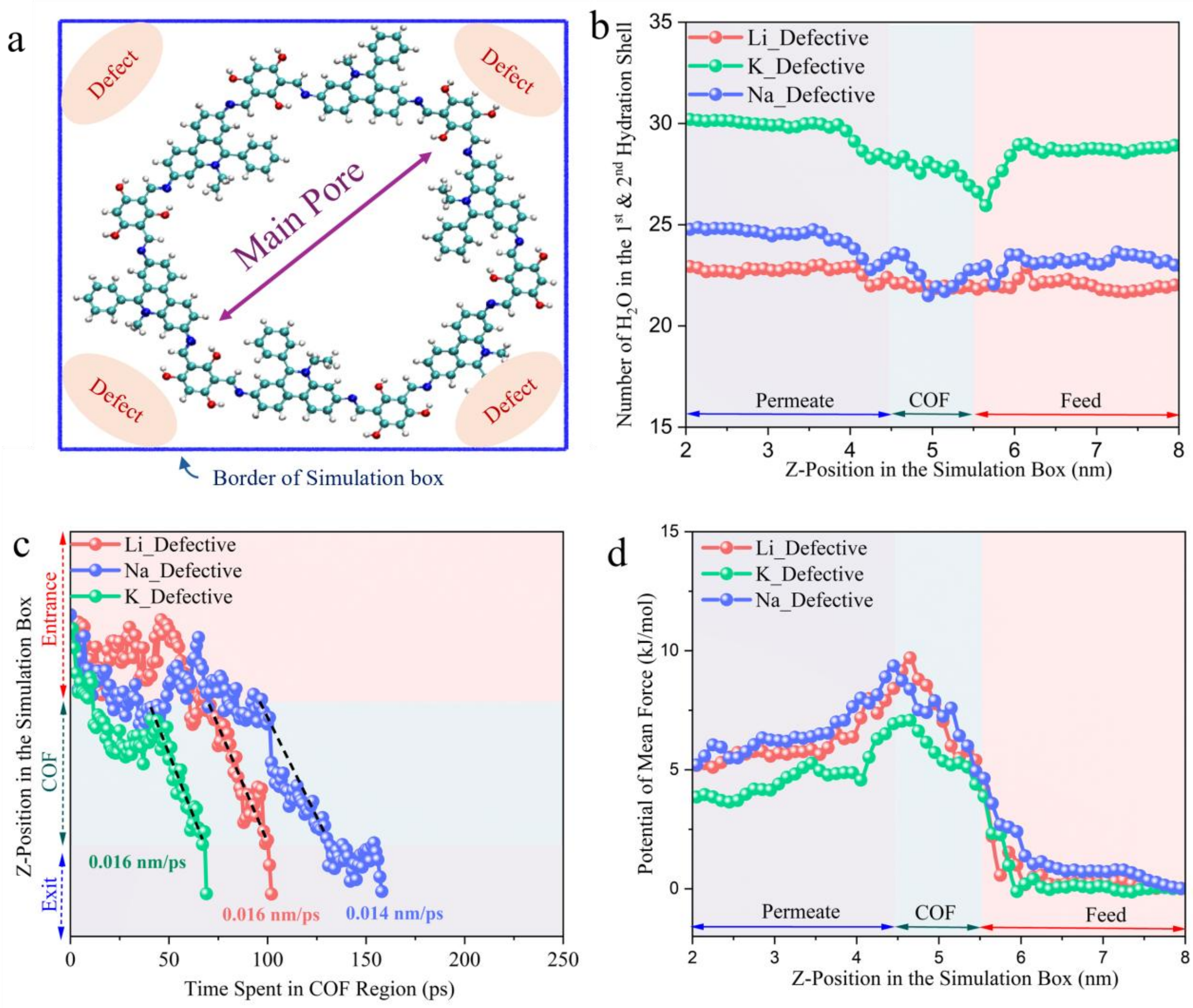

Figure S36. Molecular dynamics (MD) simulation results for the defective COF membrane. (a) Schematic illustration of the defective COF structure used in the MD simulations, highlighting the main pore and defect regions. (b) Number of water molecules in the hydration shells of the ions as a function of their position along the simulation box. (c) Ion trajectories along the z-direction as a function of simulation time, where the slopes correspond to ion velocities along the z-axis. (d) Potentials of mean force (PMFs) of the ions as a function of their position along the z-direction of the simulation box. See Supplementary Discussion 2.5 for more details.

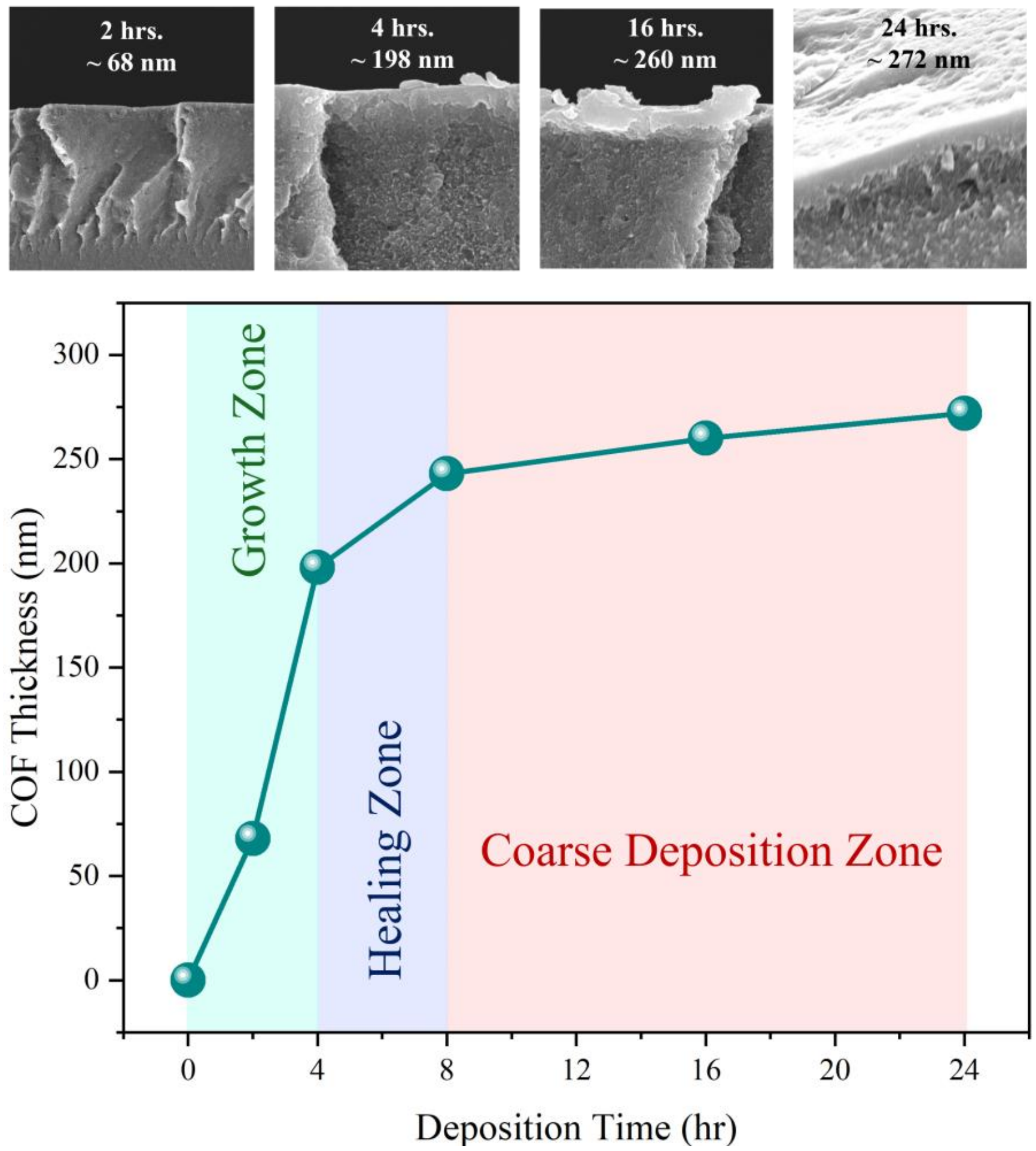

Figure S37. Evolution of COF coating thickness with deposition time.

Cross-sectional SEM images (top) and the corresponding thickness measurements (bottom) reveal three distinct kinetic regimes. During the first four hours (growth zone), the coating thickness increases rapidly, from approximately 68.0 nm at 2.0 h to 198.0 nm at 4.0 h, as COF nuclei form and spread laterally across the CEM surface, giving a discontinuous, island-like morphology. Between approximately 4.0 and 8.0 hours (healing zone), the growth rate falls sharply, with the thickness increasing by only about 50.0 nm; in this regime deposition is redirected toward the cracks, defects, and gaps between the isolated islands formed during the growth stage, progressively infilling them to yield a continuous, defect-free COF layer. Beyond 8.0 hours (coarse-deposition zone), once continuous coverage is established, the electrically resistive, positively charged coating attenuates both the interfacial field and the flux of hydronium ions that catalyze interfacial condensation; further growth therefore proceeds only through the slower, uncatalyzed condensation of COF-forming monomers from the bulk solution, thickening the film to approximately 272.0 nm at 24.0 h at a substantially reduced rate without improving coverage or separation performance(Figure S28).

4. Supplementary Tables

Table S1. Specifications of the IEMs used in this work.

Characteristics	Cation Exchange Membrane (CEM)	Anion Exchange Membrane (AEM)
Name	ASTOM® (CMX-fg)	ASTOM® (AMX-fg)
Type	Strong acid (Na Type)	Strong base (Cl Type)
Characteristics	High mechanical strength	High mechanical strength
Burst strength (MPa)	≥ 0.35	≥ 0.35
Thickness (mm)	0.16	0.15

Table S2. Synthesized COF structures and the corresponding monomers.

COF Structure	First Monomer	Amount (mmol)	Second Monomer	Amount (mmol)
$TpPa(SO_3H)_6$	2,5-Diaminobenzene-1,4-disulfonic acid	0.15	2,4,6-trihydroxy-1,3,5-benzenetricarbaldehyde	0.10
$TpPa(SO_3H)_3$	2,5-Diaminobenzenesulfonic acid	0.15	2,4,6-trihydroxy-1,3,5-benzenetricarbaldehyde	0.10
TpPa	p-Phenylenediamine	0.15	2,4,6-trihydroxy-1,3,5-benzenetricarbaldehyde	0.10
TpHz	Hydrazine	0.15	2,4,6-trihydroxy-1,3,5-benzenetricarbaldehyde	0.10
TpEb	Ethidium bromide	0.15	2,4,6-trihydroxy-1,3,5-benzenetricarbaldehyde	0.10

Table S3. Synthesis parameters for the COF-deposited TFC membranes.

Sample Code	Type of the COF	Reaction Period (hr)	Type of Voltage	Applied Voltage (Vpp)	Applied Frequency (Hz)
TFC 1	TpEb	2.0	Square	10.0	0.001
TFC 2	TpEb	8.0	Square	10.0	0.001
TFC 3	TpEb	24.0	Square	10.0	0.001
TFC 4	TpEb	8.0	Sin	10.0	0.001
TFC 5	TpEb	8.0	Ramp	10.0	0.001
TFC 6	TpEb	8.0	Square	2.0	0.001
TFC 7	TpEb	8.0	Square	20.0	0.001
TFC 9	TpEb	8.0	Square	10.0	1.0
TFC 10	TpEb	8.0	Square	10.0	1000.0
TFC 11	$TpPa(SO_3H)_6$	8.0	Square	10.0	0.001
TFC 12	$TpPa(SO_3H)_3$	8.0	Square	10.0	0.001
TFC 13	TpPa	8.0	Square	10.0	0.001
TFC 14	TpHz	8.0	Square	10.0	0.001
TFC 15	TpEb	4.0	DC	10.0	-
TFC 16	TpPa	4.0	DC	10.0	-
TFC 17	TpHz	4.0	DC	10.0	-
TFC 18*	TpHz	8.0	Square	10.0	0.001

* 300 µl of octanoic acid was added as the external catalyst (see Figure S12).

Table S4. Initial and boundary conditions used in CFD simulations.

Parameter	Description	Value	Unit
C_{H+}	Concentration of H^+ in chamber	1.0×10^{-4}	mol m^{-3}
C_{OH-}	Concentration of OH^- in chamber	1.0×10^{-4}	mol m^{-3}
pH	Initial pH value in chamber	7.0	-
C_{Cl-}	Concentration of Cl^- in chamber	1.0	M
C_{Na+}	From electroneutrality	-	M
C_{Pa-}	Concentration of Pa^- in chamber	0.75	mmol L^{-1}
C_{TP}	Concentration of Tp in chamber	0.5	mmol L^{-1}
C_{Cof}	Concentration of COF in chamber	0.01	mmol L^{-1}
V	Applied voltage (DC)	20.0	V

Table S5. Dimensional and material properties used in CFD simulations.

Parameter	Description	Value	Unit	Reference
W_{ch}	Width of the chamber	5.0	mm	Measured
L_{ch}	Length of the chamber	50.0	mm	Measured
Th_{IEM}	Thickness of the CEM	150.0	µm	Measured
D_{H+}	Diffusion coefficient of H^+	9.5×10^{-9}	$m^2\ s^{-1}$	[46]
D_{OH-}	Diffusion coefficient of OH^-	5.4×10^{-9}	$m^2\ s^{-1}$	[46]
D_{Pa-}	Diffusion coefficient of Pa^-	30.2×10^{-9}	$m^2\ s^{-1}$	MD simulation
D_{Tp}	Diffusion coefficient of Tp	14.3×10^{-9}	$m^2\ s^{-1}$	MD simulation
D_{Cof}	Diffusion coefficient of COF	2.0×10^{-12}	$m^2\ s^{-1}$	Stokes-Einstein [11]
ω	Rotating speed	1500*2*π	rad min^{-1}	Measured
Re	Reynolds number	1983.0	-	Calculated
F	Faraday's constant	96485.0	C mol^{-1}	Constant
R	Universal gas constant	8.314	J mol^{-1} K^{-1}	Constant
T	Temperature	298.15	K	Measured
Wu	Water uptake	0.29	-	[47]
ε	Porosity of membrane	0.07	-	[48,49]
IEC	Ion exchange capacity	1.66	(meq g^{-1})	[50]
Th_{CEM}	Thickness of the CEM	160.0	µm	[51]

Table S6. Applied electrical waveforms on positive and negative electrodes for AC CFD simulations (20 V case).

Electrode	Square wave	Sinusoidal wave	Ramp wave
Positive	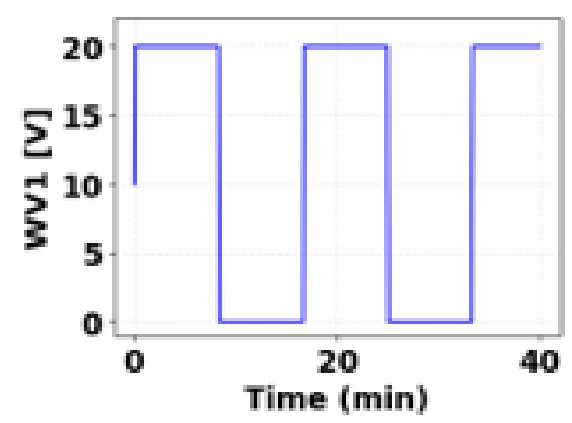	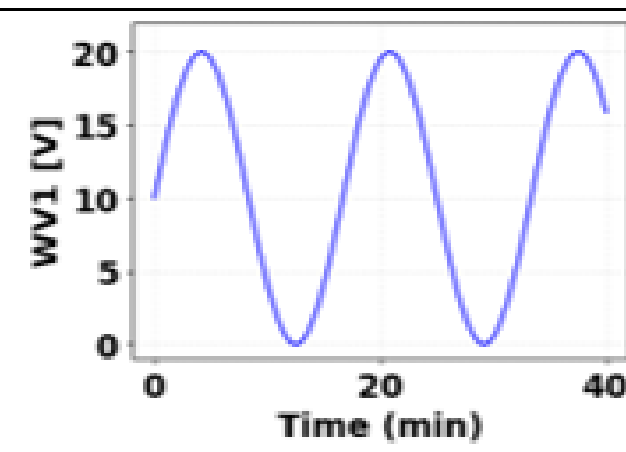	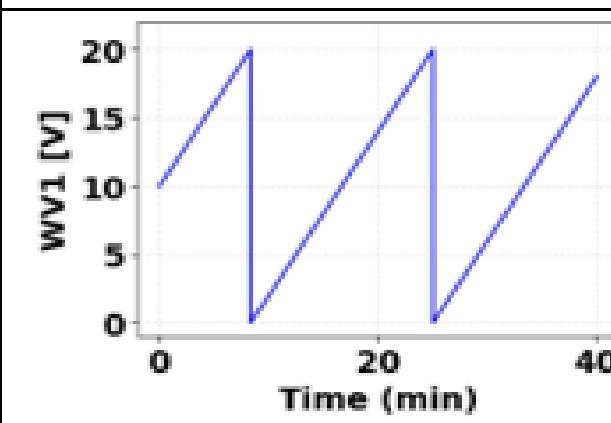
Negative	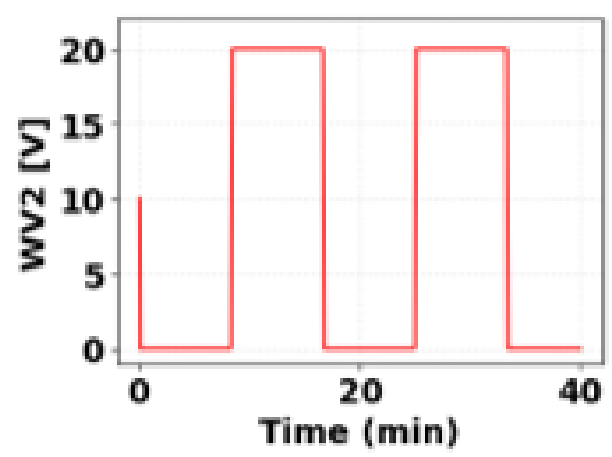	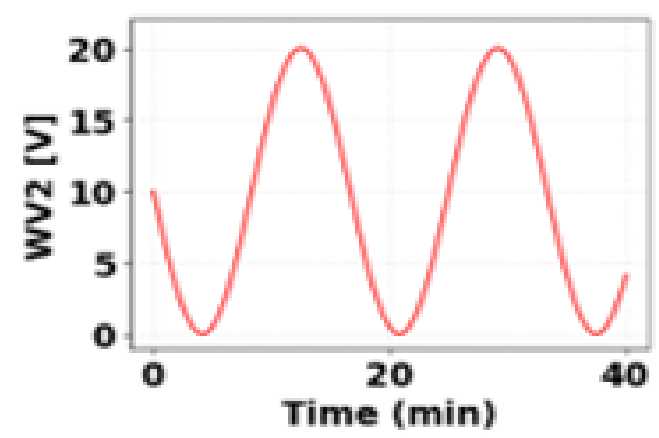	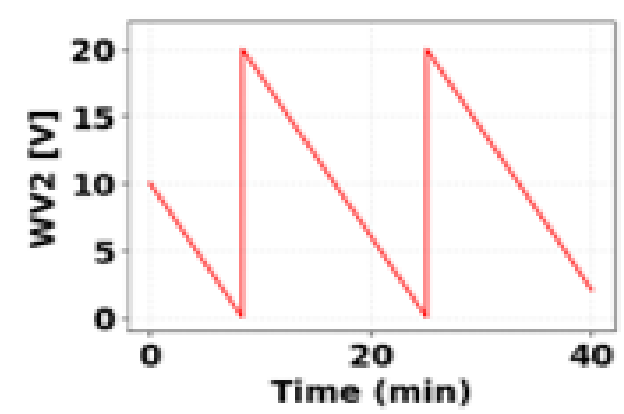

Table S7. Quantitative XPS analysis showing the surface sulfur signal attenuation of COF-based TFC membranes.

The decreasing intensity of the sulfur signal (greater sulfur-signal attenuation) reflects improved COF coverage. Comparison of the results confirms that sample TFC 2, synthesized under AC conditions (8.0 h, 10.0 Vpp, square waveform, 1.0 mHz), demonstrates the lowest detectable sulfur content and therefore the highest surface coverage and coating quality.

Sample Code	Surface sulfur signal attenuation (%)*
TFC 1	81.5 ± 0.3
TFC 2	99.2 ± 0.1
TFC 3	83.1 ± 0.2
TFC 4	84.1 ± 0.8
TFC 5	70.3 ± 0.4
TFC 6	63.6 ± 0.4
TFC 7	72.7 ± 0.5
TFC 8	87.8 ± 0.2

*Surface sulfur signal attenuation = (1- (detected sulfur (wt%)/detected sulfur in the CEM used as support (wt%)))*100

Table S8. The results of ion separation performance of all COF-based TFC membranes synthesized in this work.

Fabrication condition: voltage = 10.0 Vpp, duration: 8.0 hr, waveform: square, frequency: 1.0 mHz. For mono/divalent ion separation, the ED operating conditions were constant applied voltage of 1.5 V, initial current of approximately 2.0 mA, and operation time of 120.0 min. For mono/monovalent ion separation the ED operating conditions were constant applied voltage of 1.0 V, initial current of approximately 1.0 mA, and operation time of 60.0 min. Note that the membranes shown here are only to demonstrate the feasibility of the method, and the membrane fabrication needs to be optimized to achieve optimal structure for each chemistry.

Coated COF	Code	Feed	Permselectivity			Flux
			Li/Na	Li/K	Li/Mg	(mol m^{-2} h^{-1})
TpPa	TFC 13	LiCl (45.0 mM) / NaCl (5.0 mM)	3.84±0.03	-	-	0.08±0.01
		LiCl (45.0 mM) / KCl (5.0 mM)	-	1.03±0.10	-	0.17±0.04
		$MgCl_2$ (45.0 mM) / LiCl (5.0 mM)	-	-	116±18.0	0.01±0.005
$TpPa(SO_3H)_3$	TFC 12	LiCl (45.0 mM) / NaCl (5.0 mM)	2.63±0.02	-	-	0.29±0.07
		LiCl (45.0 mM) / KCl (5.0 mM)	-	1.08±0.09	-	0.42±0.09
		$MgCl_2$ (45.0 mM) / LiCl (5.0 mM)	-	-	15.6±2.40	0.06±0.01
$TpPa(SO_3H)_6$	TFC 11	LiCl (45.0 mM) / NaCl (5.0 mM)	1.72±0.06	-	-	0.29±0.06
		LiCl (45.0 mM) / KCl 5.0 mM)	-	1.26±0.07	-	0.48±0.09
		$MgCl_2$ (45.0 mM) / LiCl (5.0 mM)	-	-	0.81±0.11	0.11±0.03
TpHz	TFC 14	LiCl (45.0 mM) / NaCl (5.0 mM)	1.78±0.08	-	-	0.08±0.02
		LiCl (45.0 mM) / KCl (5.0 mM)	-	1.66±0.03	-	0.14±0.04
		$MgCl_2$ (45.0 mM) / LiCl(5.0 mM)	-	-	108±12.4	0.07±0.01

5. Supplementary Movies

Movie 1. CFD results for ramp potential

Simulated transient evolution of hydronium, charged monomer (Pa), non-charged monomer (Tp) and COF concentrations next to the surface of the CEMs while a ramp potential has been applied to the electrodes.

Movie 2. CFD results for sinusoidal potential

Simulated transient evolution of hydronium, charged monomer (Pa), non-charged monomer (Tp) and COF concentrations next to the surface of the CEMs while a sinusoidal potential has been applied to the electrodes.

Movie 3. CFD results for square potential

Simulated transient evolution of hydronium, charged monomer (Pa), non-charged monomer (Tp) and COF concentrations next to the surface of the CEMs while a square potential has been applied to the electrodes.

6. References

[1] M. P. Seah, "Simple Universal Curve for the Energy-Dependent Electron Attenuation Length for All Materials," *Surface and Interface Analysis 44*, no. 10 (**2012**): 1353–1359. https://doi.org/10.1002/sia.5033

[2] M. P. Seah, "A System for the Intensity Calibration of Electron Spectrometers," *Journal of Electron Spectroscopy and Related Phenomena 71*, no. 3 (**1995**): 191–204. https://doi.org/10.1016/0368-2048(94)02275-5

[3] W. Zhang, J. Ma, P. Wang, et al., "Investigations on the Interfacial Capacitance and the Diffusion Boundary Layer Thickness of Ion Exchange Membrane Using Electrochemical Impedance Spectroscopy," *Journal of Membrane Science 502* (**2016**): 37–47. https://doi.org/10.1016/j.memsci.2015.12.007

[4] J.-S. Park, J.-H. Choi, J.-J. Woo, S.-H. Moon, "An Electrical Impedance Spectroscopic (EIS) Study on Transport Characteristics of Ion-Exchange Membrane Systems," *Journal of Colloid and Interface Science 300*, no. 2 (**2006**): 655–662. https://doi.org/10.1016/j.jcis.2006.04.040

[5] W. Zhang, P. Wang, J. Ma, et al., "Investigations on Electrochemical Properties of Membrane Systems in Ion-Exchange Membrane Transport Processes by Electrochemical Impedance Spectroscopy and Direct Current Measurements," *Electrochimica Acta 216* (**2016**): 110–119. https://doi.org/10.1016/j.electacta.2016.09.018

[6] J. Wang, J. Zhang, P. Jin, et al., "A Covalent Organic Framework Membrane with Highly Selective and Permeable Artificial Sodium Channels via Ion Recognition," *Nature Communications 16*, no. 1 (**2025**): 7346. https://doi.org/10.1038/s41467-025-62329-1

[7] J. P. Perdew, K. Burke, M. Ernzerhof, "Generalized Gradient Approximation Made Simple," *Physical Review Letters 77*, no. 18 (**1996**): 3865–3868. https://doi.org/10.1103/PhysRevLett.77.3865

[8] S. Grimme, S. Ehrlich, L. Goerigk, "Effect of the Damping Function in Dispersion Corrected Density Functional Theory," *Journal of Computational Chemistry 32*, no. 7 (**2011**): 1456–1465. https://doi.org/10.1002/jcc.21759

[9] A. Bergner, M. Dolg, W. Küchle, et al., "Ab Initio Energy-Adjusted Pseudopotentials for Elements of Groups 13–17," *Molecular Physics 80*, no. 6 (**1993**): 1431–1441. https://doi.org/10.1080/00268979300103121

[10] M. Dolg, U. Wedig, H. Stoll, et al., "Energy-Adjusted Ab Initio Pseudopotentials for the First Row Transition Elements," *The Journal of Chemical Physics 86*, no. 2 (**1987**): 866–872. https://doi.org/10.1063/1.452288

[11] F. Yang, J. Guo, C. Han, et al., "Turing Covalent Organic Framework Membranes via Heterogeneous Nucleation Synthesis for Organic Solvent Nanofiltration," *Science Advances 10*, no. 50 (**2024**): eadr9260. https://doi.org/10.1126/sciadv.adr9260

[12] Y. Zhang, J. Guo, G. Han, et al., "Molecularly Soldered Covalent Organic Frameworks for Ultrafast Precision Sieving," *Science Advances 7*, no. 13 (**2021**): eabe8706. https://doi.org/10.1126/sciadv.abe8706

[13] S. Grimme, J. G. Brandenburg, C. Bannwarth, et al., "Consistent Structures and Interactions by Density Functional Theory with Small Atomic Orbital Basis Sets," *The Journal of Chemical Physics 143*, no. 5 (**2015**): 054107. https://doi.org/10.1063/1.4927476

[14] N. Mardirossian, M. Head-Gordon, "Exploring the Limit of Accuracy for Density Functionals Based on the Generalized Gradient Approximation: Local, Global Hybrid, and Range-Separated Hybrid Functionals with and without Dispersion Corrections," *The Journal of Chemical Physics 140*, no. 18 (**2014**): 18A527. https://doi.org/10.1063/1.4868117

[15] T. Lu, "A Comprehensive Electron Wavefunction Analysis Toolbox for Chemists, Multiwfn," *The Journal of Chemical Physics 161*, no. 8 (**2024**): 082503. https://doi.org/10.1063/5.0216272

[16] T. Lu, F. Chen, "Multiwfn: A Multifunctional Wavefunction Analyzer," *Journal of Computational Chemistry 33*, no. 5 (**2012**): 580–592. https://doi.org/10.1002/jcc.22885

[17] G. Bussi, D. Donadio, M. Parrinello, "Canonical Sampling through Velocity Rescaling," *The Journal of Chemical Physics 126*, no. 1 (**2007**): 014101. https://doi.org/10.1063/1.2408420

[18] M. Bernetti, G. Bussi, "Pressure Control Using Stochastic Cell Rescaling," *The Journal of Chemical Physics 153*, no. 11 (**2020**): 114107. https://doi.org/10.1063/5.0020514

[19] E. J. Maginn, R. A. Messerly, D. J. Carlson, et al., "Best Practices for Computing Transport Properties 1. Self-Diffusivity and Viscosity from Equilibrium Molecular Dynamics [Article v1.0]," *Living Journal of Computational Molecular Science 1*, no. 1 (**2019**): 6324. https://doi.org/10.33011/livecoms.1.1.6324

[20] P. Li, K. M. Merz, "Taking into Account the Ion-Induced Dipole Interaction in the Nonbonded Model of Ions," *Journal of Chemical Theory and Computation 10*, no. 1 (**2014**): 289–297. https://doi.org/10.1021/ct400751u

[21] P. Li, L. F. Song, K. M. Merz, “Systematic Parameterization of Monovalent Ions Employing the Nonbonded Model,” *Journal of Chemical Theory and Computation 11*, no. 4 (**2015**): 1645–1657. https://doi.org/10.1021/ct500918t

[22] P. Loche, P. Steinbrunner, S. Friedowitz, et al., “Transferable Ion Force Fields in Water from a Simultaneous Optimization of Ion Solvation and Ion–Ion Interaction,” *The Journal of Physical Chemistry B 125*, no. 30 (**2021**): 8581–8587. https://doi.org/10.1021/acs.jpcb.1c05303

[23] M. Wang, Y. Wang, J. Zhao, et al., “Electrochemical Interfacial Polymerization toward Ultrathin COF Membranes for Brine Desalination,” *Angewandte Chemie International Edition 62*, no. 13 (**2023**): e202219084. https://doi.org/10.1002/anie.202219084

[24] A. M. Gado, R. J. Ouimet, L. Bonville, et al., “Recombination Layers for Effective Hydrogen Crossover Mitigation in Proton Exchange Membrane Water Electrolyzers: Fabrication, Characterization, and Fundamental Principles of Operation,” *Electrochimica Acta 525* (**2025**): 146092. https://doi.org/10.1016/j.electacta.2025.146092

[25] C. Klose, P. Trinke, T. Böhm, et al., “Membrane Interlayer with Pt Recombination Particles for Reduction of the Anodic Hydrogen Content in PEM Water Electrolysis,” *Journal of The Electrochemical Society 165*, no. 16 (**2018**): F1271–F1277. https://doi.org/10.1149/2.1241814jes

[26] H. Liu, J. T. Lang, F. Babbe, et al., “Mitigation of Hydrogen Crossover in Liquid Alkaline Water Electrolysers Using Gas Recombination Catalysts,” *Nature Energy* (**2026**), advance online publication. https://doi.org/10.1038/s41560-026-02094-7

[27] A. T. S. Freiberg, S. Thiele, “Closing the Hydrogen and Oxygen Mass Balance for PEM Water Electrolysis,” *Journal of The Electrochemical Society 172*, no. 3 (**2025**): 034506. https://doi.org/10.1149/1945-7111/adb7c8

[28] D. L. Martinho, M. Abdollahzadehsangroudi, S. S. Araya, et al., “Eulerian-Eulerian CFD Modeling of Multiphase Flow and Heat Transfer in Alkaline Electrolysis Cells,” *Journal of The Electrochemical Society 172*, no. 6 (**2025**): 064501. https://doi.org/10.1149/1945-7111/addb7f

[29] A. Zarghami, N. G. Deen, A. W. Vreman, “CFD Modeling of Multiphase Flow in an Alkaline Water Electrolyzer,” *Chemical Engineering Science 227* (**2020**): 115926. https://doi.org/10.1016/j.ces.2020.115926

[30] G. Vázquez, E. Álvarez, J. M. Navaza, “Surface Tension of Alcohol Water + Water from 20 to 50 °C,” *Journal of Chemical & Engineering Data 40*, no. 3 (**1995**): 611–614. https://doi.org/10.1021/je00019a016

[31] M. Ponomar, E. Krasnyuk, D. Butylskii, et al., "Sessile Drop Method: Critical Analysis and Optimization for Measuring the Contact Angle of an Ion-Exchange Membrane Surface," *Membranes 12*, no. 8 (**2022**): 765. https://doi.org/10.3390/membranes12080765

[32] A. Angulo, P. van der Linde, H. Gardeniers, et al., "Influence of Bubbles on the Energy Conversion Efficiency of Electrochemical Reactors," *Joule 4*, no. 3 (**2020**): 555–579. https://doi.org/10.1016/j.joule.2020.01.005

[33] S. Wu, J. Qiu, J. Wang, et al., "Covalent Organic Framework Membranes Modified by End-Capping Monomers for Organic Solvent Nanofiltration," *Journal of Membrane Science 703* (**2024**): 122854. https://doi.org/10.1016/j.memsci.2024.122854

[34] R. Tan, A. Wang, R. Malpass-Evans, et al., "Hydrophilic Microporous Membranes for Selective Ion Separation and Flow-Battery Energy Storage," *Nature Materials 19*, no. 2 (**2020**): 195–202. https://doi.org/10.1038/s41563-019-0536-8

[35] W. Xin, C. Lin, L. Fu, et al., "Nacre-like Mechanically Robust Heterojunction for Lithium-Ion Extraction," *Matter 4*, no. 2 (**2021**): 737–754. https://doi.org/10.1016/j.matt.2020.12.003

[36] X. Shi, Z. Zhang, S. Fang, et al., "Flexible and Robust Three-Dimensional Covalent Organic Framework Membranes for Precise Separations under Extreme Conditions," *Nano Letters 21*, no. 19 (**2021**): 8355–8362. https://doi.org/10.1021/acs.nanolett.1c02919

[37] J. Lu, H. Zhang, J. Hou, et al., "Efficient Metal Ion Sieving in Rectifying Subnanochannels Enabled by Metal–Organic Frameworks," *Nature Materials 19*, no. 7 (**2020**): 767–774. https://doi.org/10.1038/s41563-020-0634-7

[38] C. E. Ren, K. B. Hatzell, M. Alhabeb, et al., "Charge- and Size-Selective Ion Sieving Through Ti3C2Tx MXene Membranes," *The Journal of Physical Chemistry Letters 6*, no. 20 (**2015**): 4026–4031. https://doi.org/10.1021/acs.jpclett.5b01895

[39] Q. Wen, D. Yan, F. Liu, et al., "Highly Selective Ionic Transport through Subnanometer Pores in Polymer Films," *Advanced Functional Materials 26*, no. 32 (**2016**): 5796–5803. https://doi.org/10.1002/adfm.201601689

[40] J. Abraham, K. S. Vasu, C. D. Williams, et al., "Tunable Sieving of Ions Using Graphene Oxide Membranes," *Nature Nanotechnology 12*, no. 6 (**2017**): 546–550. https://doi.org/10.1038/nnano.2017.21

[41] T. Xu, B. Wu, L. Hou, et al., "Highly Ion-Permselective Porous Organic Cage Membranes with Hierarchical Channels," *Journal of the American Chemical Society 144*, no. 23 (**2022**): 10220–10229. https://doi.org/10.1021/jacs.2c00318

[42] Y.-H. Xi, Z. Liu, J. Ji, et al., "Graphene-Based Membranes with Uniform 2D Nanochannels for Precise Sieving of Mono-/Multi-Valent Metal Ions," *Journal of Membrane Science 550* (**2018**): 208–218. https://doi.org/10.1016/j.memsci.2017.12.057

[43] F. Sheng, B. Wu, X. Li, et al., "Efficient Ion Sieving in Covalent Organic Framework Membranes with Sub-2-Nanometer Channels," *Advanced Materials 33*, no. 44 (**2021**): 2104404. https://doi.org/10.1002/adma.202104404

[44] Z. Lu, Y. Wu, L. Ding, et al., "A Lamellar MXene (Ti3C2Tx)/PSS Composite Membrane for Fast and Selective Lithium-Ion Separation," *Angewandte Chemie International Edition 60*, no. 41 (**2021**): 22265–22269. https://doi.org/10.1002/anie.202108801

[45] L. Huang, H. Wu, L. Ding, et al., "Shearing Liquid-Crystalline MXene into Lamellar Membranes with Super-Aligned Nanochannels for Ion Sieving," *Angewandte Chemie International Edition 63*, no. 6 (**2024**): e202314638. https://doi.org/10.1002/anie.202314638

[46] Y. Litman, A. Michaelides, "Entropy Governs the Structure and Reactivity of Water Dissociation Under Electric Fields," *Journal of the American Chemical Society 147*, no. 49 (**2025**): 44885–44894. https://doi.org/10.1021/jacs.5c12397

[47] H. Asgari, F. Ghavipanjeh, M. R. Sabour, et al., "Fabrication of Pore-Filling Cation-Exchange Membrane from Waste Polystyrene and Spunbond Meltblown Spunbond (SMS) Non-Woven Polypropylene Fabric as the Substrate," *Scientific Reports 14*, no. 1 (**2024**): 6399. https://doi.org/10.1038/s41598-024-56961-y

[48] N. Kononenko, V. Nikonenko, D. Grande, et al., "Porous Structure of Ion Exchange Membranes Investigated by Various Techniques," *Advances in Colloid and Interface Science 246* (**2017**): 196–216. https://doi.org/10.1016/j.cis.2017.05.007

[49] Q. Lang, Y. Liu, G. Guo, et al., "Electrochemical Characterization and Simulation of Ion Transport in Anion Exchange Membranes for Water Treatment Applications," *Membranes 15*, no. 4 (**2025**): 123. https://doi.org/10.3390/membranes15040123

[50] S. Sahin, J. E. Dykstra, H. Zuilhof, et al., "Modification of Cation-Exchange Membranes with Polyelectrolyte Multilayers to Tune Ion Selectivity in Capacitive Deionization," *ACS Applied Materials & Interfaces 12*, no. 31 (**2020**): 34746–34754. https://doi.org/10.1021/acsami.0c05664

[51] C. Chan, N. Zamel, X. Li, et al., "Experimental Measurement of Effective Diffusion Coefficient of Gas Diffusion Layer/Microporous Layer in PEM Fuel Cells," *Electrochimica Acta 65* (**2012**): 13–21. https://doi.org/10.1016/j.electacta.2011.12.110
